# Clinical diagnosis of genetic disorders at both single-nucleotide and chromosomal levels based on BGISEQ-500 platform

**DOI:** 10.1038/s41439-023-00238-9

**Published:** 2023-05-22

**Authors:** Yanqiu Liu, Liangwei Mao, Hui Huang, Wei Li, Jianfen Man, Wenqian Zhang, Lina Wang, Long Li, Yan Sun, Teng Zhai, Xueqin Guo, Lique Du, Jin Huang, Hao Li, Yang Wan, Xiaoming Wei

**Affiliations:** 1 Department of Genetics, Jiangxi Maternal and Child Health Hospital, 330006 Nanchang, China; 2 BGI-Anhui Clinical Laboratory, BGI-Shenzhen, 236000 Fuyang, China; 3 The State Key Laboratory of Biocatalysis and Enzyme Engineering, College of Life Sciences, Hubei University, 430062 Wuhan, China; 4 BGI Genomics, BGI-Shenzhen, 518083 Shenzhen, China; 5 BGI-Wuhan Clinical Laboratory, BGI-Shenzhen, 430074 Wuhan, China; 6 Department of Biology, University of Copenhagen, Copenhagen, DK-2200 Denmark; 7 Department of Obstetrics and Gynecology, Fuyang People’s Hospital, 236000 Fuyang, China

**Keywords:** Medical genetics, Genetic testing

## Abstract

Most variations in the human genome refer to single-nucleotide variation (SNV), small fragment insertions and deletions, and genomic copy number variation (CNV). Many human diseases including genetic disorders are associated with variations in the genome. These disorders are often difficult to be diagnosed because of their complex clinical conditions, therefore, an effective detection method is needed to facilitate clinical diagnosis and prevent birth defects. With the development of high-throughput sequencing technology, the method of targeted sequence capture chip has been extensively used owing to its high throughput, high accuracy, fast speed, and low cost. In this study, we designed a chip that potentially captured the coding region of 3043 genes associated with 4013 monogenic diseases, with an addition of 148 chromosomal abnormalities that can be identified by targeting specific regions. To assess the efficiency, a strategy of combining the BGISEQ500 sequencing platform with the designed chip was utilized to screen variants in 63 patients. Eventually, 67 disease-associated variants were found, 31 of which were novel. The results of the evaluation test also show that this combined strategy complies with the requirements of clinical testing and has proper clinical application value.

## Genetic testing: One-step targeted sequencing helps diagnose genetic disorders

A new DNA screening method potentially offers a one-step solution to the challenge of diagnosing genetic diseases. A team from China led by Xiaoming Wei from BGI Genomics in Shenzhen and Yang Wan from the Fuyang People’s Hospital has designed a DNA chip that captures the coding regions of 4,013 disease-causing genes plus another 148 common chromosomal abnormalities. The targeted next-generation sequencing strategy can detect a variety of genomic alterations including single-letter mutations as well as chromosomal abnormalities including insertions, deletions and duplications of larger pieces of DNA. The researchers ran the test on blood samples from 63 patients with genetic disorders. In 52 of these individuals, they found a total of 67 different disease-related variants, including 31 novel variants, demonstrating the clinical utility of the approach.

## Introduction

Monogenic inherited diseases usually involve multiple disciplines and complex clinical symptoms. They are difficult to be precisely diagnosed by conventional clinical tests due to the underlying molecular mechanisms, and most of them are usually fatal, disabling, or teratogenic^1^. Traditional testing techniques may have a greater risk of bringing in false negative diagnosis and misdiagnosis, as a result, the clinicians may miss the critical points to provide treatment for the patients. In comparison, genetic testing can achieve better performance including early detection, early intervention, and early treatment for single-gene genetic diseases. Large-scale discovery of novel genes and validation of monogenic diseases can be quickly implemented and widely applied clinically. People with a family history of genetic disorders can be screened by pre-marital, pre-pregnancy, and prenatal genetic screening^2,3^ and avoid birth defects. Therefore, genetic testing is important for clinical diagnosis and prevention of birth defects.

Next-generation sequencing technology has been widely used in detecting genetic disease. The major sequencing technologies are targeted region sequencing, whole exome sequencing, whole genome sequencing, and mitochondrial DNA sequencing. However, whole genome and exome sequencing are not only costly and time consuming, but also challenging to screen for specific disease-causing variantsacross a large span of genomic region^4^. The combination of regional capture and high-throughput sequencing technology can effectively capture disease-associated regions and quickly locate disease-causing variants. With the characteristics of high throughput, low cost^5^, high speed, and high accuracy, high-throughput sequencing technology is widely used in clinical practice^6,7^ for genetic disease detection and carrier screening^8^. However, most of the currently available products for genetic testing detect limited types of diseases and have a compromised detection rate^9^. Moreover, besides monogenic variants, recent studies have found that chromosome microdeletions or microduplications are important causes of developmental delay and intellectual disability^10^. Therefore, we urgently need a highly efficient and sensitive screening method that can detect all types of variants to meet the need of one-step detection of a variety of monogenic genetic diseases and common chromosomal abnormalities.

Therefore, this study used BGISEQ-500 as a sequencing platform to develop a chip that focuses on coding regions with known associations with genetic diseases. Variants that affect gene function are detected more cost-effectively than whole genome sequencing or whole exome sequencing. Currently, 4013 known single genetic diseases can be detected (Table 1). In addition, we can detect 148 common chromosomal abnormalities by targeting specific regions (Table 2). Compared with traditional gene detection methods, the combined strategy integrates known single-gene diseases with common chromosomal abnormalities, and therefore achieves “one-step” solution to detecting genetic variants. The improved detection rate of diseases, along with the benefit of high throughput, high accuracy, fast speed, and low cost proves that this combined strategy is a powerful tool for clinical diagnosis and prenatal prevention of birth defects.

**Table 1:** List of 4013 diseases that can be detected by the designed chip.

OMIM	Disease	Gene
606864	Paraganglioma and Gastric Stromal Sarcoma	SDHB, SDHC, SDHD
616415	Familial adenomatous polyposis 3	NTHL1
617100	Familial adenomatous polyposis 4	MSH3
158350	Cowden syndrome 1	PTEN
612359	Cowden syndrome 2	SDHB
615106	Cowden syndrome 3	SDHD
615107	Cowden syndrome 4	KLLN
615108	Cowden syndrome 5	PIK3CA
615109	Cowden syndrome 6	AKT1
616858	Cowden syndrome 7	SEC23B
193300	Von Hippel-Lindau Disease	VHL
135150	Birt-Hogg-Dubé Syndrome	FLCN
160980	Carney Complex	PRKAR1A
109400	Nevoid Basal Cell Carcinoma Syndrome	PTCH1
194070	Wilms Tumor 1	WT1
150800	Hereditary Leiomyomatosis and Renal Cell Cancer	FH
601606	Multiple Familial Trichoepithelioma 1	CYLD
211900	Hyperphosphatemic Familial Tumoral Calcinosis	FGF23, GALNT3, KL
608266	Parathyroid Carcinoma	CDC73
112250	Diaphyseal Medullary Stenosis with Malignant Fibrous Histiocytoma	MTAP
151623	Li-Fraumeni Syndrome 1	TP53
609265	Li-Fraumeni Syndrome 2	CHEK2
608615	Oligodontia-Colorectal Cancer Syndrome	AXIN2
180200	Retinoblastoma	RB1
609322	Rhabdoid Tumor Predisposition Syndrome 1	SMARCB1
613325	Rhabdoid Tumor Predisposition Syndrome 2	SMARCA4
614327	Tumor Predisposition Syndrome	BAP1
148500	Tylosis with Esophageal Cancer	RHBDF2
608837	Carney Complex Variant	MYH8
155255	Medulloblastoma	SUFU, PTCH2, BRCA2
155755	Melanoma-Astrocytoma Syndrome	CDKN2A
614165	Familial Paragangliomas 5	SDHA
155240	Familial Medullary Thyroid Carcinoma	NTRK1, RET
202300	Adrenocortical Carcinoma, Hereditary	TP53
114900	Intestinal Carcinoid Tumors	SDHD
215300	Chondrosarcoma	EXT1
135290	Desmoid Disease, Hereditary	APC
615554	Multiple Fibroadenomas Of The Breast	PRLR
255960	Intracardiac Myxoma	PRKAR1A
259500	Osteogenic Sarcoma	TP53
260500	Papilloma Of Choroid Plexus	TP53
601518	Prostate Cancer, Hereditary, 1	RNASEL
268220	Rhabdomyosarcoma 2	PAX3
180295	Rhabdomyosarcoma, Embryonal, 2	DICER1
181030	Pleomorphic Salivary Gland Adenoma	PLAG1
275355	Head And Neck Squamous Cell Carcinoma	TNFRSF10B
610455	Familial Normophosphatemic Tumoral Calcinosis	SAMD9
158320	Muir-Torre syndrome	MLH1, MSH2
212065	Congenital Disorders of Glycosylation Ia	PMM2
602579	Congenital Disorders of Glycosylation Ib	MPI
603147	Congenital Disorders of Glycosylation Ic	ALG6
601110	Congenital Disorders of Glycosylation Id	ALG3
608799	Congenital Disorders of Glycosylation Ie	DPM1
609180	Congenital Disorders of Glycosylation If	MPDU1
607143	Congenital Disorders of Glycosylation Ig	ALG12
608104	Congenital Disorders of Glycosylation Ih	ALG8
607906	Congenital Disorders of Glycosylation Ii	ALG2
608093	Congenital Disorders of Glycosylation Ij	DPAGT1
608540	Congenital Disorders of Glycosylation Ik	ALG1
608776	Congenital Disorders of Glycosylation Il	ALG9
610768	Congenital Disorders of Glycosylation Im	DOLK
612015	Congenital Disorders of Glycosylation In	RFT1
612937	Congenital Disorders of Glycosylation Io	DPM3
613661	Congenital Disorders of Glycosylation Ip	ALG11
612379	Congenital Disorders of Glycosylation Iq	SRD5A3
614507	Congenital Disorders of Glycosylation Ir	DDOST
300884	Congenital Disorders of Glycosylation Is	ALG13
614921	Glycogen Storage Disease type XIV	PGM1
615042	Congenital Disorders of Glycosylation Iu	DPM2
615273	Congenital Disorders of Glycosylation Iv	NGLY1
615596	Congenital Disorders of Glycosylation Iw	STT3A
615597	Congenital Disorders of Glycosylation Ix	STT3B
300934	Congenital Disorders of Glycosylation Iy	SSR4
616457	Congenital Disorders of Glycosylation Iz	CAD
212066	Congenital Disorders of Glycosylation IIa	MGAT2
606056	Congenital Disorders of Glycosylation IIb	MOGS
266265	Congenital Disorders of Glycosylation IIc	SLC35C1
607091	Congenital Disorders of Glycosylation IId	B4GALT1
608779	Congenital Disorders of Glycosylation IIe	COG7
603585	Congenital Disorders of Glycosylation IIf	SLC35A1
611209	Congenital Disorders of Glycosylation IIg	COG1
611182	Congenital Disorders of Glycosylation IIh	COG8
613612	Congenital Disorders of Glycosylation IIi	COG5
613489	Congenital Disorders of Glycosylation IIj	COG4
614727	Congenital Disorders of Glycosylation IIk	TMEM165
614576	Congenital Disorders of Glycosylation IIl	COG6
300896	Congenital Disorders of Glycosylation IIm	SLC35A2
616721	Congenital Disorders of Glycosylation IIN	SLC39A8
616828	Congenital Disorders of Glycosylation IIO	CCDC115
616829	Congenital Disorders of Glycosylation IIP	TMEM199
250950	3-Methylglutaconic Aciduria type 1	AUH
302060	Barth Syndrome	TAZ
258501	Optic Atrophy plus Syndrome	OPA3
610198	3-Methylglutaconic Aciduria type 5	DNAJC19
617248	3-methylglutaconic aciduria, type VIII	HTRA2
614739	3-Methylglutaconic aciduria with deafness, encephalopathy, and Leigh-like syndrome	SERAC1
607015	Hurler-Scheie Syndrome	IDUA
309900	Mucopolysaccharidosis II	IDS
252900	Mucopolysaccharidosis type IIIA	SGSH
252920	Mucopolysaccharidisis type IIIB	NAGLU
252940	Mucopolysaccharidosis type IIID	GNS
253000	Mucopolysaccharidosis type IVA	GALNS
253010	Mucopolysaccharidosis type IVB	GLB1
607016	Scheie Syndrome	IDUA
253200	Mucopolysaccharidosis type VI	ARSB
253220	Mucopolysaccharidosis type VII	GUSB
601492	Mucopolysaccharidosis type IX	HYAL1
257200	Niemann-Pick Disease A	SMPD1
607616	Niemann-Pick Disease B	SMPD1
257220	Niemann-Pick Disease type C1	NPC1
607625	Niemann-Pick Disease type C2	NPC2
214100	Peroxisome biogenesis disorder 1A	PEX1
601539	Peroxisome biogenesis disorder 1B	PEX1
214110	Peroxisome biogenesis disorder 2A	PEX5
202370	Peroxisome biogenesis disorder 2B	PEX5
614859	Peroxisome biogenesis disorder 3A	PEX12
266510	Peroxisome biogenesis disorder 3B	PEX12
614862	Peroxisome biogenesis disorder 4A	PEX6
614863	Peroxisome biogenesis disorder 4B	PEX6
614866	Peroxisome biogenesis disorder 5A	PEX2
614867	Peroxisome biogenesis disorder 5B	PEX2
614872	Peroxisome biogenesis disorder 7A	PEX26
614873	Peroxisome biogenesis disorder 7B	PEX26
614876	Peroxisome biogenesis disorder 8A	PEX16
614877	Peroxisome biogenesis disorder 8B	PEX16
614879	Peroxisome biogenesis disorder 9B	PEX7
614882	Peroxisome biogenesis disorder 10A	PEX3
617370	Peroxisome biogenesis disorder 10B	PEX3
614883	Peroxisome biogenesis disorder 11A	PEX13
614885	Peroxisome biogenesis disorder 11B	PEX13
614886	Peroxisome biogenesis disorder 12A	PEX19
614887	Peroxisome biogenesis disorder 13A	PEX14
614920	Peroxisome Biogenesis Disorder 14B	PEX11B
232200	Glycogen Storage Disease type Ia	G6PC
232220	Glycogen Storage Disease type Ib	SLC37A4
232240	Glycogen Storage Disease Ic	SLC37A4
232300	Glycogen storage disease II	GAA
232400	Glycogen Storage Disease type III	AGL
232500	Glycogen Storage Disease type IV	GBE1
232600	Glycogen Storage Disease type V	PYGM
232700	Glycogen Storage Disease type VI	PYGL
232800	Glycogen Storage Disease type VII	PFKM
306000	Glycogen storage disease type IXa1	PHKA2
261750	Glycogen storage disease type IXb	PHKB
613027	Glycogen storage disease type IXc	PHKG2
300559	Glycogen storage disease type IXd	PHKA1
261670	Glycogen Storage Disease type X	PGAM2
612933	Glycogen Storage Disease type XI	LDHA
611881	Glycogen Storage Disorder type XII	ALDOA
612932	Glycogen Storage Disease type XIII	ENO3
613507	Glycogen Storage Disease type XV	GYG1
611556	Glycogen Storage Disease 0, Muscle	GYS1
240600	Glycogen Storage Disease 0, Liver	GYS2
261740	Glycogen storage disease of heart, lethal congenital	PRKAG2
300257	Danon disease	LAMP2
227810	Fanconi-Bickel Syndrome	SLC2A2
236200	Homocystinuria Caused by Cystathionine Beta-Synthase Deficiency	CBS
612740	Acute Hepatic Porphyria	ALAD
176200	Porphyria Variegata	PPOX
176100	Hepatoerythropoietic Porphyria	UROD
300752	X-Linked Protoporphyria	ALAS2
121300	Hereditary Coproporphyria	CPOX
176000	Acute Intermittent Porphyria	HMBS
263700	Congenital Erythropoietic Porphyria	UROS
237300	Carbamoylphosphate Synthetase I Deficiency	CPS1
311250	Ornithine Transcarbamylase Deficiency	OTC
215700	Citrullinemia type I	ASS1
603471	Citrullinemia, adult-onset type II	SLC25A13
207900	Argininosuccinic Aciduria	ASL
207800	Arginase Deficiency	ARG1
238970	Hyperornithinemia-Hyperammonemia-Homocitrullinuria Syndrome	SLC25A15
237310	N-Acetylglutamate Synthase Deficiency	NAGS
235200	Hemochromatosis, Type 1	HFE
602390	Hemochromatosis, Type 2A	HFE2
613313	Hemochromatosis, Type 2B	HAMP
604250	Hemochromatosis, Type 3	TFR2
606069	Hemochromatosis, Type 4	SLC40A1
248600	Maple syrup urine disease, type II/Ia/Ib	BCKDHA, BCKDHB, DBT
246900	Maple syrup urine disease, type III	DLD
615135	Mild Variant Maple Syrup Urine Disease	PPM1K
276700	Tyrosinemia Type I	FAH
276600	Tyrosinemia Type II	TAT
276710	Tyrosinemia Type III	HPD
256550	Sialidosis	NEU1
252500	Mucolipidosis II Alpha&Beta	GNPTAB
252600	Mucolipidosis III Alpha&Beta	GNPTAB
252605	Mucolipidosis III Gamma	GNPTG
252650	Mucolipidosis IV	MCOLN1
255120	Carnitine Palmitoyltransferase I Deficiency	CPT1A
608836	Carnitine Palmitoyltransferase II Deficiency	CPT2
600649	Carnitine palmitoyltransferase II deficiency, severe infantile form	CPT2
255110	Carnitine palmitoyltransferase II deficiency, myopathic form	CPT2
259900	Primary Hyperoxaluria Type I	AGXT
260000	Primary Hyperoxaluria Type II	GRHPR
613616	Primary Hyperoxaluria Type III	HOGA1
263570	Adult Polyglucosan Body Disease	GBE1
604369	Free Sialic Acid Storage Disorders	SLC17A5
269920	Free sialic acid storage disease, infantile form	SLC17A5
230000	Fucosidosis	FUCA1
245200	Krabbe Disease	GALC
611722	Krabbe Disease, Atypical, due to Saposin A Deficiency	PSAP
272200	Multiple Sulfatase Deficiency	SUMF1
261515	Peroxisomal Bifunctional Enzyme Deficiency	HSD17B4
264470	Peroxisomal Acyl-CoA oxidase deficiency	ACOX1
177735	Pseudohypoaldosteronism Type IA	NR3C2
264350	Pseudohypoaldosteronism Type IB	SCNN1A, SCNN1B, SCNN1G
614491	Pseudohypoaldosteronism Type IIB	WNK4
614492	Pseudohypoaldosteronism Type IIC	WNK1
614495	Pseudohypoaldosteronism Type IID	KLHL3
614496	Pseudohypoaldosteronism Type IIE	CUL3
307800	X-Linked Hypophosphatemia	PHEX
146300	Hypophosphatasia, adult	ALPL
241510	Hypophosphatasia, childhood	ALPL
241500	Hypophosphatasia, infantile	ALPL
609060	Combined Oxidative Phosphorylation Deficiency 1	GFM1
610498	Combined Oxidative Phosphorylation Deficiency 2	MRPS16
610505	Combined Oxidative Phosphorylation Deficiency 3	TSFM
610678	Combined Oxidative Phosphorylation Deficiency 4	TUFM
611719	Combined Oxidative Phosphorylation Deficiency 5	MRPS22
300816	Combined Oxidative Phosphorylation Deficiency 6	AIFM1
613559	Combined Oxidative Phosphorylation Deficiency 7	C12orf65
614096	Combined Oxidative Phosphorylation Deficiency 8	AARS2
614582	Combined Oxidative Phosphorylation Deficiency 9	MRPL3
614702	Combined Oxidative Phosphorylation Deficiency 10	MTO1
614922	Combined Oxidative Phosphorylation Deficiency 11	RMND1
614932	Combined Oxidative Phosphorylation Deficiency 13	PNPT1
614946	Combined Oxidative Phosphorylation Deficiency 14	FARS2
614947	Combined Oxidative Phosphorylation Deficiency 15	MTFMT
615395	Combined Oxidative Phosphorylation Deficiency 16	MRPL44
615440	Combined Oxidative Phosphorylation Deficiency 17	ELAC2
615578	Combined Oxidative Phosphorylation Deficiency 18	SFXN4
615917	Combined Oxidative Phosphorylation Deficiency 20	VARS2
615918	Combined Oxidative Phosphorylation Deficiency 21	TARS2
616045	Combined Oxidative Phosphorylation Deficiency 22	ATP5A1
616198	Combined Oxidative Phosphorylation Deficiency 23	GTPBP3
616239	Combined Oxidative Phosphorylation Deficiency 24	NARS2
616430	Combined Oxidative Phosphorylation Deficiency 25	MARS2
616539	Combined Oxidative Phosphorylation Deficiency 26	TRMT5
616672	Combined Oxidative Phosphorylation Deficiency 27	CARS2
616794	Combined Oxidative Phosphorylation Deficiency 28	SLC25A26
616811	Combined Oxidative Phosphorylation Deficiency 29	TXN2
616974	Combined Oxidative Phosphorylation Deficiency 30	TRMT10C
239510	Hyperprolinemia type II	ALDH4A1
251000	Methylmalonic Aciduria Due To Methylmalonyl-Coa Mutase Deficiency	MUT
612073	Mitochondrial DNA depletion syndrome 5	SUCLA2
245400	Mitochondrial DNA depletion syndrome 9	SUCLG1
617184	Mitochondrial DNA depletion syndrome 12A (cardiomyopathic type)	SLC25A4
614265	Combined Malonic and Methylmalonic Aciduria	ACSF3
277400	Methylmalonic aciduria and homocystinuria CblC type	MMACHC
277410	Methylmalonic aciduria and homocystinuria CblD type	MMADHC
277380	Methylmalonic aciduria and homocystinuria CblF type	LMBRD1
614857	Methylmalonic aciduria and homocystinuria CblJ type	ABCD4
309541	Methylmalonic acidemia with homocystinuria CblX type	HCFC1
251100	Methylmalonic Acidemia, CblA Type	MMAA
251110	Methylmalonic Acidemia, CblB Type	MMAB
251120	Methylmalonyl-Coa Epimerase Deficiency	MCEE
613646	Methylmalonic Aciduria due to Transcobalamin Receptor Defect	CD320
612949	Early Infantile Epileptic Encephalopathy 39	SLC25A12
617106	Early Infantile Epileptic Encephalopathy 42	CACNA1A
617113	Early Infantile Epileptic Encephalopathy 43	GABRB3
617389	Early Infantile Epileptic Encephalopathy 53	SYNJ1
617391	Early Infantile Epileptic Encephalopathy 54	HNRNPU
616834	Microcephaly, Congenital Cataract, And Psoriasiform Dermatitis	MSMO1
253270	Multiple carboxylase deficiency	HLCS
256730	Neuronal Ceroid-Lipofuscinoses 1	PPT1
204500	Neuronal Ceroid-Lipofuscinoses 2	TPP1
204200	Neuronal Ceroid-Lipofuscinoses 3	CLN3
162350	Neuronal Ceroid-Lipofuscinoses 4B	DNAJC5
610951	Neuronal Ceroid-Lipofuscinoses 7	MFSD8
610003	Neuronal Ceroid-Lipofuscinoses 8,Northern epilepsy variant	CLN8
600143	Neuronal Ceroid Lipofuscinosis 8	CLN8
610127	Neuronal Ceroid-Lipofuscinoses 10	CTSD
614706	Neuronal Ceroid-Lipofuscinoses 11	GRN
606693	Kufor-Rakeb syndrome	ATP13A2
615362	Neuronal Ceroid-Lipofuscinoses 13	CTSF
230400	Galactosemia	GALT
256540	Galactosialidosis	CTSA
231670	Glutaric Acidemia I	GCDH
231680	Glutaric Acidemia II	ETFA, ETFB, ETFDH
231690	Glutaric Aciduria III	SUGCT
612736	Guanidinoaceteate Methyltransferase Deficiency	GAMT
300352	SLC6A8-Related Creatine Transporter Deficiency	SLC6A8
265120	Pulmonary Surfactant Metabolism Dysfunction 1	SFTPB
610913	Pulmonary Surfactant Metabolism Dysfunction 2	SFTPC
610921	Pulmonary Surfactant Metabolism Dysfunction 3	ABCA3
614370	Pulmonary Surfactant Metabolism Dysfunction 5	CSF2RB
236250	Homocystinuria due to MTHFR deficiency	MTHFR
250940	Homocystinuria-Megaloblastic Anemia CblG type	MTR
236270	Homocystinuria-megaloblastic anemia CblE type	MTRR
230500	GM1-gangliosidosis	GLB1
272750	GM2-gangliosidosis, AB variant	GM2A
230600	GM1-Gangliosidosis, Type II	GLB1
230650	GM1-Gangliosidosis, Type III	GLB1
272800	Tay-Sachs Disease	HEXA
268800	Sandhoff Disease	HEXB
309400	Menkes Disease	ATP7A
614723	Adenine Phosphoribosyltransferase Deficiency	APRT
203500	Alkaptonuria	HGD
248500	Alpha-Mannosidosis	MAN2B1
613490	Alpha1-Antitrypsin Deficiency	SERPINA1
261600	Phenylketonuria	PAH
300661	Phosphoribosylpyrophosphate Synthetase Superactivity	PRPS1
606054	Propionic Acidemia	PCCA, PCCB
266150	Pyruvate Carboxylase Deficiency	PC
201470	Short Chain Acyl-CoA Dehydrogenase Deficiency	ACADS
269921	Sialuria	GNE
212140	Primary Carnitine Deficiency	SLC22A5
602079	Trimethylaminuria	FMO3
201475	Very Long-Chain Acyl-Coenzyme A Dehydrogenase Deficiency	ACADVL
277900	Wilson Disease	ATP7B
219800	Nephropathic Cystinosis	CTNS
613571	Cytochrome P450 Oxidoreductase Deficiency	POR
223360	Dopamine Beta-Hydroxylase Deficiency	DBH
230350	Epimerase Deficiency Galactosemia	GALE
301500	Fabry Disease	GLA
606812	Fumarase Deficiency	FH
201450	Medium-Chain Acyl-Coenzyme A Dehydrogenase Deficiency	ACADM
312170	Pyruvate dehydrogenase E1-alpha deficiency	PDHA1
210200	3-Methylcrotonyl-CoA carboxylase 1 deficiency	MCCC1
210210	3-Methylcrotonyl-CoA carboxylase 2 deficiency	MCCC2
611283	Isobutyryl-CoA dehydrogenase deficiency	ACAD8
610006	2-Methylbutyryl Glycinuria	ACADSB
203750	Beta-Ketothiolase Deficiency	ACAT1
208400	Aspartylglucosaminuria	AGA
229600	Hereditary Fructose Intolerance	ALDOB
274270	Dihydropyrimidine Dehydrogenase Deficiency	DPYD
243500	Isovaleric Acidemia	IVD
250850	Hypermethioninemia	MAT1A
609015	Trifunctional Protein Deficiency	HADHA, HADHB
609016	Long-Chain 3-Hydroxyacyl-Coa Dehydrogenase Deficiency	HADHA
248360	Malonyl-Coa Decarboxylase Deficiency	MLYCD
266130	Glutathione synthetase deficiency	GSS
217090	Congenital plasminogen deficiency	PLG
266200	Pyruvate kinase deficiency	PKLR
612718	Arginine:Glycine Amidinotransferase Deficiency	GATM
610377	Mevalonic Aciduria	MVK
609734	Proopiomelanocortin Deficiency	POMC
278000	Cholesteryl Ester Storage Disease	LIPA
610984	Complement Factor I Deficiency	CFI
218800	Crigler-Najjar syndrome type 1	UGT1A1
606785	Crigler-Najjar syndrome type 2	UGT1A1
237900	Transient Familial Neonatal Hyperbilirubinemia	UGT1A1
143500	Gilbert Syndrome	UGT1A1
608782	Pyruvate Dehydrogenase Phosphatase Deficiency	PDP1
608643	Aromatic L-Amino Acid Decarboxylase Deficiency	DDC
124000	Mitochondrial Complex III Deficiency Nuclear type 1	BCS1L
603358	Gracile Syndrome	BCS1L
235800	Histidinemia	HAL
245349	Pyruvate Dehydrogenase E3-Binding Protein Deficiency	PDHX
245348	Pyruvate Dehydrogenase E2 Deficiency	DLAT
300908	Glucose-6-Phosphate Dehydrogenase Deficiency	G6PD
212138	Carnitine-Acylcarnitine Translocase Deficiency	SLC25A20
261640	BH4-Deficient Hyperphenylalaninemia A	PTS
233910	BH4-Deficient Hyperphenylalaninemia B	GCH1
261630	BH4-Deficient Hyperphenylalaninemia C	QDPR
264070	BH4-Deficient Hyperphenylalaninemia D	PCBD1
600721	D-2-hydroxyglutaric aciduria 1	D2HGDH
236792	L-2-hydroxyglutaric aciduria	L2HGDH
615182	D,L-2-hydroxyglutaric aciduria	SLC25A1
264600	Steroid 5-Alpha-Reductase Deficiency	SRD5A2
615511	Adenosine Monophosphate Deaminase Deficiency	AMPD1
248510	Beta-Mannosidosis	MANBA
275630	Chanarin-Dorfman syndrome	ABHD5
245900	Familial Lecithin cholesterol acyltransferase deficiency	LCAT
222900	Congenital Sucrase-Isomaltase Deficiency	SI
237500	Dubin-Johnson syndrome	ABCC2
604091	Familial HDL Deficiency	ABCA1, APOA1
270400	Smith-Lemli-Opitz syndrome	DHCR7
263800	Gitelman syndrome	SLC12A3
229100	Glutamate Formiminotransferase Deficiency	FTCD
264300	17-beta Hydroxysteroid Dehydrogenase 3 Deficiency	HSD17B3
201810	3-beta-Hydroxysteroid Dehydrogenase Deficiency	HSD3B2
238700	Hyperlysinemia	AASS
614128	Lactate Dehydrogenase B Deficiency	LDHB
300653	Phosphoglycerate Kinase Deficiency	PGK1
170100	Prolidase deficiency	PEPD
210250	Sitosterolemia	ABCG5, ABCG8
245050	Succinyl-CoA:3-ketoacid CoA Transferase Deficiency	OXCT1
205400	Tangier Disease	ABCA1
613118	Hereditary Antithrombin-III Deficiency	SERPINC1
607426	Primary Coenzyme Q10 deficiency 1	COQ2
614651	Primary Coenzyme Q10 deficiency 2	PDSS1
614652	Primary Coenzyme Q10 deficiency 3	PDSS2
612016	Primary Coenzyme Q10 deficiency 4	ADCK3
614654	Primary Coenzyme Q10 deficiency 5	COQ9
614650	Primary Coenzyme Q10 deficiency 6	COQ6
616276	Primary Coenzyme Q10 deficiency 7	COQ4
616733	Primary Coenzyme Q10 deficiency 8	COQ7
256731	Neuronal Ceroid-Lipofuscinoses 5	CLN5
253260	Biotinidase Deficiency	BTD
266500	Refsum disease	PHYH, PEX7
610539	Gaucher Disease, Atypical, due to Saposin C Deficiency	PSAP
608013	Gaucher Disease, Perinatal Lethal	GBA
230800	Gaucher disease type 1	GBA
230900	Gaucher Disease, Type II	GBA
231000	Gaucher Disease, Type III	GBA
231005	Gaucher Disease, Type IIIC	GBA
246450	3-hydroxy-3-methylglutaryl-CoA lyase deficiency	HMGCL
605911	3-Hydroxy-3-Methylglutaryl-CoA Synthase 2 Deficiency	HMGCS2
614097	Acatalasemia	CAT
613933	Acetyl-CoA Carboxylase Deficiency	ACACA
614055	Acetyl-CoA Carboxylase-Beta Deficiency	ACAT2
615961	Acid-Labile Subunit Deficiency	IGFALS
611126	Acyl-CoA Dehydrogenase 9 Deficiency	ACAD9
105200	Familial Visceral Amyloidosis	APOA1, LYZ, FGA
105210	Familial Transthyretin Amyloidosis	TTR
105120	Finnish type Amyloidosis	GSN
615558	Hypobetalipoproteinemia, Familial, 1	APOB
603813	Autosomal Recessive Familial Hypercholesterolemia	LDLRAP1
603776	Familial Hypercholesterolemia 3	PCSK9
144010	Autosomal Dominant Hypercholesterolemia type B	APOB
207750	Apolipoprotein C-II Deficiency	APOC2
614028	Apolipoprotein C-III Deficiency	APOC3
615501	Molybdenum cofactor deficiency C	GPHN
614200	Glycoprotein 1a Deficiency	ITGA2
614923	Branched-chain Ketoacid Dehydrogenase Kinase Deficiency	BCKDK
613021	Bronchiectasis with or without Elevated Sweat Chloride 2	SCNN1A
613071	Bronchiectasis with or without Elevated Sweat Chloride 3	SCNN1G
143470	Hyperalphalipoproteinemia 1	CETP
614122	Chitotriosidase Deficiency	CHIT1
613546	Aromatase Deficiency	CYP19A1
611721	Combined Saposin Deficiency	PSAP
604931	Cortisone Reductase Deficiency 1	H6PD
614662	Cortisone Reductase Deficiency 2	HSD11B1
219500	Cystathioninuria	CTH
220100	Cystinuria	SLC3A1, SLC7A9
609153	Familial Pseudohyperkalemia	ABCB6
222730	Dicarboxylic aminoaciduria	SLC1A1
222748	Dihydropyrimidinase Deficiency	DPYS
612874	Erythrocyte AMP Deaminase Deficiency	AMPD3
145980	Familial Hypocalciuric Hypercalcemia, Type I	CASR
145981	Familial Hypocalciuric Hypercalcemia Type II	GNA11
600740	Familial Hypocalciuric Hypercalcemia Type III	AP2S1
143880	Infantile Hypercalcemia 1	CYP24A1
616963	Infantile Hypercalcemia 2	SLC34A1
151660	Familial Partial Lipodystrophy Type 2	LMNA
604367	Familial Partial Lipodystrophy Type 3	PPARG
613877	Familial Partial Lipodystrophy Type 4	PLIN1
615238	Familial Partial Lipodystrophy Type 5	CIDEC
615980	Familial Partial Lipodystrophy Type 6	LIPE
601399	Familial Platelet Disorder with associated Myeloid Malignancy	RUNX1
604377	Fatal Infantile Cardioencephalomyopathy due to Cytochrome c Oxidase Deficiency 1	SCO2
615119	Fatal Infantile Cardioencephalomyopathy due to Cytochrome c Oxidase Deficiency 2	COX15
616500	Fatal Infantile Cardioencephalomyopathy due to Cytochrome c Oxidase Deficiency 3	COA5
616501	Fatal Infantile Cardioencephalomyopathy due to Cytochrome c Oxidase Deficiency 4	COA6
229700	Fructose 1,6 Bisphosphatase Deficiency	FBP1
229800	Essential Fructosuria	KHK
613163	GABA-Transaminase Deficiency	ABAT
230200	Galactokinase Deficiency	GALK1
610015	Congenital Glutamine Deficiency	GLUL
307030	Glycerol Kinase Deficiency	GK
606664	Glycine N-Methyltransferase Deficiency	GNMT
300323	Kelley-Seegmiller syndrome	HPRT1
234500	Hartnup Disease	SLC6A19
140350	Hawkinsinuria	HPD
614025	Hepatic Lipase Deficiency	LIPC
229050	Hereditary Folate Malabsorption	SLC46A1
143860	Isolated Hyperchlorhidrosis	CA12
614300	Hypermethioninemia due to Adenosine Kinase Deficiency	ADK
613752	Hypermethioninemia with S-Adenosylhomocysteine Hydrolase Deficiency	AHCY
240900	Hypoinsulinemic Hypoglycemia with Hemihypertrophy	AKT2
154020	Hypomagnesemia 2, Renal	FXYD2
248250	Hypomagnesemia 3, Renal	CLDN16
611718	Hypomagnesemia 4, Renal	EGF
248190	Hypomagnesemia 5, Renal	CLDN19
613882	Hypomagnesemia 6, Renal	CNNM2
607330	Lathosterolosis	SC5D
614962	Leptin Deficiency	LEP
614963	Leptin Receptor Deficiency	LEPR
246650	Combined Lipase Deficiency	LMF1
614105	Methylmalonate Semialdehyde Dehydrogenase Deficiency	ALDH6A1
604290	Aceruloplasminemia	CP
610773	Mitochondrial phosphate carrier deficiency	SLC25A3
252150	Sulfite oxidase deficiency due to molybdenum cofactor deficiency type A	MOCS1
252160	Sulfite oxidase deficiency due to molybdenum cofactor deficiency type B	MOCS2
610717	Neutral Lipid Storage Disease with Myopathy	PNPLA2
258900	Orotic Aciduria	UMPS
614338	Pancreatic Lipase Deficiency	PNLIP
261680	Cytosolic Phosphoenolpyruvate Carboxykinase Deficiency	PCK1
601815	Phosphoglycerate Dehydrogenase Deficiency	PHGDH
610992	Phosphoserine Aminotransferase Deficiency	PSAT1
614023	Phosphoserine Phosphatase Deficiency	PSPH
614111	Pyruvate Dehydrogenase E1-Beta Deficiency	PDHB
614462	Pyruvate Dehydrogenase Lipoic Acid Synthetase Deficiency	LIAS
608611	Ribose 5-Phosphate Isomerase Deficiency	RPIA
268900	Sarcosinemia	SARDH
609241	Alpha-N-acetylgalactosaminidase deficiency	NAGA
138500	Hyperglycinuria	SLC36A2
272300	Sulfocysteinuria	SUOX
606003	Transaldolase Deficiency	TALDO1
275350	Transcobalamin II Deficiency	TCN2
615512	Triosephosphate Isomerase Deficiency	TPI1
278300	Xanthinuria, Type I	XDH
608118	Transient Neonatal Zinc Deficiency	SLC30A2
103050	Adenylosuccinase Deficiency	ADSL
614307	Alpha-Methylacyl-CoA Racemase Deficiency	AMACR
609924	Aminoacylase 1 Deficiency	ACY1
613161	Beta-Ureidopropionase Deficiency	UPB1
258870	Ornithine Aminotransferase Deficiency	OAT
238600	Hyperlipoproteinemia type I	LPL
615947	Hyperlipoproteinemia type ID	GPIHBP1
617347	Hyperlipoproteinemia type III	APOE
144650	Hyperlipoproteinemia type V	APOA5
177000	Erythropoietic Protoporphyria	FECH
615812	Abdominal Obesity-Metabolic Syndrome 3	DYRK1B
204750	2-Aminoadipic 2-Oxoadipic Aciduria	DHTKD1
616271	3-Methylglutaconic Aciduria With Cataracts, Neurologic Involvement,And Neutropenia	CLPB
231530	3-Hydroxyacyl-Coa Dehydrogenase Deficiency	HADH
250620	3-Hydroxyisobutyryl-Coa Hydrolase Deficiency	HIBCH
260005	5-Oxoprolinase Deficiency	OPLAH
608688	Aicar Transformylase/Imp Cyclohydrolase Deficiency	ATIC
615574	Asparagine Synthetase Deficiency	ASNS
222800	Bisphosphoglycerate Mutase Deficiency	BPGM
211180	Bowen-Conradi Syndrome	EMG1
615751	Hyperammonemia Due To Carbonic Anhydrase Va Deficiency	CA5A
212070	Carboxypeptidase N Deficiency	CPN1
605814	Citrullinemia, Type II, Neonatal-Onset	SLC25A13
123320	Creatine Phosphokinase, Elevated Serum	CAV3
220120	D-Glyceric Aciduria	GLYCTK
605850	Dimethylglycine Dehydrogenase Deficiency	DMGDH
261500	Eosinophil Peroxidase Deficiency	EPX
245340	Erythrocyte Lactate Transporter Defect	SLC16A1
136120	Fish-Eye Disease	LCAT
610293	Glycosylphosphatidylinositol Deficiency	PIGM
607014	Hurler Syndrome	IDUA
236800	Hydroxykynureninuria	KYNU
614156	Hyperbiliverdinemia	BLVRA
115300	Autosomal Dominant Hypercarotenemia And Vitamin A Deficiency	BCMO1
144250	Familial Combined Hyperlipidemia	LPL
616214	Hyperproinsulinemia	INS
615555	Hyperprolactinemia	PRLR
614480	Transient Infantile Hypertriglyceridemia	GPD1
605019	Familial Hypobetalipoproteinemia 2	ANGPTL3
607236	Hypoprebetalipoproteinemia, Acanthocytosis, Retinitis Pigmentosa,And Pallidal Degeneration	PANK2
613850	Inosine Triphosphatase Deficiency	ITPA
615604	L-Ferritin Deficiency	FTL
247100	Lipoid Proteinosis Of Urbach And Wiethe	ECM1
614741	Mitochondrial Pyruvate Carrier Deficiency	MPC1
616277	Mitochondrial Short-Chain Enoyl-Coa Hydratase 1 Deficiency	ECHS1
616095	Monocarboxylate Transporter 1 Deficiency	SLC16A1
613949	OKT4 Epitope Deficiency	CD4
260800	Pentosuria	DCXR
616154	Peroxisomal Fatty Acyl-Coa Reductase 1 Disorder	FAR1
615011	Phosphohydroxylysinuria	PHYKPL
615026	Riboflavin Deficiency	SLC52A1
613710	Thiamine Metabolism Dysfunction Syndrome 4 (Bilateral Striatal Degeneration and Progressive Polyneuropathy Type)	SLC25A19
614458	Thiamine Metabolism Dysfunction Syndrome 5 (Episodic Encephalopathy type)	TPK1
276880	Urocanase Deficiency	UROC1
616299	Lipoyltransferase 1 deficiency	LIPT1
610199	Neonatal Diabetes Mellitus with Congenital Hypothyroidism	GLIS3
234580	Heimler Syndrome 1	PEX1
616617	Heimler Syndrome 2	PEX6
617021	Hydrops, lactic acidosis, and sideroblastic anemia	LARS2
NA034	Pseudocholinesterase deficiency	BCHE
NA035	Succinate-CoA ligase deficiency	SUCLA2, SUCLG1
NA036	APTX-Related Coenzyme Q10 Deficiency	APTX
NA042	Hepatic Failure, Early-Onset, and Neurologic Disorder due to Cytochrome C Oxidase Deficiency	SCO1
NA044	Tryptophan Hydroxylase Deficiency	TPH2
301835	Arts Syndrome	PRPS1
311300	Otopalatodigital syndrome type 1	FLNA
304120	Otopalatodigital syndrome type 2	FLNA
222300	Wolfram Syndrome 1	WFS1
604928	Wolfram Syndrome 2	CISD2
222448	Donnai-Barrow syndrome	LRP2
610706	Congenital Deafness with Labyrinthine Aplasia, Microtia, and Microdontia	FGF3
124480	Congenital Deafness with Onychodystrophy	ATP6V1B2
218040	Costello Syndrome	HRAS
262000	Bjornstad Syndrome	BCS1L
191900	Muckle-Wells syndrome	NLRP3
149200	Bart-Pumphrey syndrome	GJB2
122880	Craniofacial-Deafness-Hand syndrome	PAX3
124900	Autosomal Dominant Deafness 1	DIAPH1
612644	Autosomal Dominant Deafness 2B	GJB3
601544	Autosomal Dominant Deafness 3A	GJB2
612643	Autosomal Dominant Deafness 3B	GJB6
600652	Autosomal Dominant Deafness 4A	MYH14
614614	Autosomal Dominant Deafness 4B	CEACAM16
600994	Autosomal Dominant Deafness 5	DFNA5
600965	Autosomal Dominant Deafness 6	WFS1
601543	Autosomal Dominant Deafness 8	TECTA
601369	Autosomal Dominant Deafness 9	COCH
601316	Autosomal Dominant Deafness 10	EYA4
601317	Autosomal Dominant Deafness 11	MYO7A
601868	Autosomal Dominant Deafness 13	COL11A2
602459	Autosomal Dominant Deafness 15	POU4F3
603622	Autosomal Dominant Deafness 17	MYH9
604717	Autosomal Dominant Deafness 20&26	ACTG1
606346	Autosomal Dominant Deafness 22	MYO6
605192	Autosomal Dominant Deafness 23	SIX1
605583	Autosomal Dominant Deafness 25	SLC17A8
608641	Autosomal Dominant Deafness 28	GRHL2
606705	Autosomal Dominant Deafness 36	TMC1
605594	Autosomal Dominant Deafness 39 with dentinogenesis Imperfecta 1	DSPP
616357	Autosomal Dominant Deafness 40	CRYM
608224	Autosomal Dominant Deafness 41	P2RX2
607453	Autosomal Dominant Deafness 44	CCDC50
607841	Autosomal Dominant Deafness 48	MYO1A
615629	Autosomal Dominant Deafness 56	TNC
614152	Autosomal Dominant Deafness 64	DIABLO
616044	Autosomal Dominant Deafness 65	TBC1D24
616969	Autosomal Dominant Deafness 66	CD164
616340	Autosomal Dominant Deafness 67	OSBPL2
616707	Autosomal Dominant Deafness 68	HOMER2
616697	Autosomal Dominant Deafness 69	KITLG
616968	Autosomal Dominant Deafness 70	MCM2
617605	Autosomal Dominant Deafness 71	DMXL2
220290	Autosomal Recessive Deafness 1A	GJB2, GJB3, GJB6
612645	Autosomal Recessive Deafness 1B	GJB6
600060	Autosomal Recessive Deafness 2	MYO7A
600316	Autosomal Recessive Deafness 3	MYO15A
600791	Autosomal Recessive Deafness 4 with enlarged vestibular aqueduct	SLC26A4, KCNJ10, FOXI1
600971	Autosomal Recessive Deafness 6	TMIE
600974	Autosomal Recessive Deafness 7	TMC1
601072	Autosomal Recessive Deafness 8	TMPRSS3
601071	Autosomal Recessive Deafness 9	OTOF
601386	Autosomal Recessive Deafness 12	CDH23
601869	Autosomal Recessive Deafness 15	GIPC3
602092	Autosomal Recessive Deafness 18A	USH1C
603629	Autosomal Recessive Deafness 21	TECTA
609533	Autosomal Recessive Deafness 23	PCDH15
611022	Autosomal Recessive Deafness 24	RDX
613285	Autosomal Recessive Deafness 25	GRXCR1
609823	Autosomal Recessive Deafness 28	TRIOBP
614035	Autosomal Recessive Deafness 29	CLDN14
607101	Autosomal Recessive Deafness 30	MYO3A
607084	Autosomal Recessive Deafness 31	DFNB31
608565	Autosomal Recessive Deafness 35	ESRRB
607821	Autosomal Recessive Deafness 37	MYO6
608265	Autosomal Recessive Deafness 39	HGF
609646	Autosomal Recessive Deafness 42	ILDR1
610154	Autosomal Recessive Deafness 44	ADCY1
609439	Autosomal Recessive Deafness 48	CIB2
610153	Autosomal Recessive Deafness 49	MARVELD2
609706	Autosomal Recessive Deafness 53	COL11A2
610220	Autosomal Recessive Deafness 59	DFNB59
613865	Autosomal Recessive Deafness 61	SLC26A5
611451	Autosomal Recessive Deafness 63	LRTOMT
610212	Autosomal Recessive Deafness 66	DCDC2
610265	Autosomal Recessive Deafness 67	LHFPL5
610419	Autosomal Recessive Deafness 68	S1PR2
614934	Autosomal Recessive Deafness 70	PNPT1
613718	Autosomal Recessive Deafness 74	MSRB3
615540	Autosomal Recessive Deafness 76	SYNE4
613079	Autosomal Recessive Deafness 77	LOXHD1
614944	Autosomal Recessive Deafness 84B	OTOGL
614617	Autosomal Recessive Deafness 86	TBC1D24
615429	Autosomal Recessive Deafness 88	ELMOD3
613916	Autosomal Recessive Deafness 89	KARS
613453	Autosomal Recessive Deafness 91	SERPINB6
614899	Autosomal Recessive Deafness 93	CABP2
616705	Autosomal Recessive Deafness 97	MET
614861	Autosomal Recessive Deafness 98	TSPEAR
615837	Autosomal Recessive Deafness 101	GRXCR2
615974	Autosomal Recessive Deafness 102	EPS8
616042	Autosomal Recessive Deafness 103	CLIC5
616515	Autosomal Recessive Deafness 104	FAM65B
616958	Autosomal Recessive Deafness 105	CDC14A
304500	X-linked Deafness 1	PRPS1
304400	X-linked Deafness 2	POU3F4
300066	X-linked Deafness 4	SMPX
300614	X-linked Deafness 5	AIFM1
300914	X-linked Deafness 6	COL4A6
274600	Pendred Syndrome	SLC26A4
221200	Deafness And Myopia	SLITRK6
300475	Deafness, Dystonia, And Cerebral Hypomyelination	BCAP31
220500	Deafness, Onychodystrophy, Osteodystrophy, Mental Retardation, And seizures Syndrome	TBC1D24
220400	Jervell and Lange-Nielsen syndrome 1	KCNQ1
612347	Jervell and Lange-Nielsen syndrome 2	KCNE1
193500	Waardenburg syndrome type 1	PAX3
193510	Waardenburg syndrome type 2A	MITF
608890	Waardenburg syndrome type 2D	SNAI2
611584	Waardenburg Syndrome Type 2E	SOX10
148820	Waardenburg syndrome type 3	PAX3
277580	Waardenburg syndrome type 4A	EDNRB
613265	Waardenburg syndrome type 4B	EDN3
613266	Waardenburg syndrome type 4C	SOX10
103470	Waardenburg syndrome&Digenic Albinism	TYR, MITF
276900	Usher Syndrome Type IB	MYO7A
276904	Usher Syndrome Type IC	USH1C
602083	Usher Syndrome Type IF	PCDH15
601067	Usher syndrome Type ID/F, Digenic	PCDH15, CDH23
606943	Usher Syndrome Type IG	USH1G
614869	Usher Syndrome Type IJ	CIB2
276901	Usher Syndrome Type IIA	USH2A
605472	Usher syndrome Type IIC	PDZD7, ADGRV1
611383	Usher Syndrome Type IID	DFNB31
276902	Usher Syndrome Type IIIA	CLRN1
614504	Usher Syndrome Type IIIB	HARS
113650	Branchiootorenal syndrome 1	EYA1
610896	Branchiootorenal syndrome 2	SIX5
166780	Otofaciocervical Syndrome 1	EYA1
615560	Otofaciocervical Syndrome 2	PAX1
602588	Branchiootic syndrome 1	EYA1
608389	Branchiootic syndrome 3	SIX1
108300	Stickler Syndrome 1	COL2A1
604841	Stickler Syndrome 2	COL11A1
184840	Stickler Syndrome 3	COL11A2
614134	Stickler Syndrome 4	COL9A1
614284	Stickler Syndrome 5	COL9A2
613076	Mitochondrial Progressive Myopathy with Congenital Cataract, Hearing Loss, and Developmental Delay	GFER
612290	Microtia, Hearing Impairment, and Cleft Palate	HOXA2
153650	Epstein Syndrome	MYH9
154780	Marshall Syndrome	COL11A1
220600	Split-Hand/Foot Malformation 1 with Sensorineural Hearing Loss	DLX5
605289	Split-Hand/Foot Malformation 4	TP63
225300	Split-Hand/Foot Malformation 6	WNT10B
103500	Tietz Syndrome	MITF
612394	Bone Fragility with Contractures, Arterial Rupture, and Deafness	PLOD3
610474	Camptodactyly, Tall Stature, and Hearing Loss Syndrome	FGFR3
600501	Abcd Syndrome	EDNRB
616007	Cataracts, Growth Hormone Deficiency, Sensory Neuropathy, sensorineural hearing loss, and skeletal dysplasia	IARS2
614482	Congenital Cataracts, Hearing Loss, And Neurodegeneration	SLC33A1
147750	Oculo-oto-radial syndrome	SALL4
600208	Macrothrombocytopenia And Progressive Sensorineural Deafness	MYH9
615381	Mandibular Hypoplasia, Deafness, Progeroid Features, And Lipodystrophy syndrome	POLD1
309350	Melnick-Needles Syndrome	FLNA
311150	Opticoacoustic Nerve Atrophy With Dementia	TIMM8A
614296	Autosomal Dominant Wolfram-Like Syndrome	WFS1
610965	Xfe Progeroid Syndrome	ERCC4
600002	Eiken Skeletal Dysplasia	PTH1R
300244	Terminal Osseous Dysplasia	FLNA
305620	Frontometaphyseal Dysplasia 1	FLNA
231095	Ghosal Hematodiaphyseal Dysplasia	TBXAS1
613330	Spondylo-Megaepiphyseal-Metaphyseal Dysplasia	NKX3-2
250400	Metaphyseal Dysplasia, Spahr type	MMP13
271665	Spondylometaepiphyseal Dysplasia, Short Limb-Hand type	DDR2
611263	Short-rib thoracic dysplasia 2 with or without polydactyly	IFT80
613091	Short-rib thoracic dysplasia 3 with or without polydactyly	DYNC2H1
613819	Short-rib thoracic dysplasia 4 with or without polydactyly	TTC21B
614376	Short-rib thoracic dysplasia 5 with or without polydactyly	WDR19
263520	Short-rib thoracic dysplasia 6 with or without polydactyly	NEK1
614091	Short-rib thoracic dysplasia 7 with or without polydactyly	WDR35
615503	Short-rib thoracic dysplasia 8 with or without polydactyly	WDR60
266920	Short-rib thoracic dysplasia 9 with or without polydactyly	IFT140
615630	Short-rib thoracic dysplasia 10 with or without polydactyly	IFT172
615633	Short-rib thoracic dysplasia 11 with or without polydactyly	WDR34
616300	Short-rib thoracic dysplasia 13 with or without polydactyly	CEP120
616546	Short-Rib Thoracic Dysplasia 14 With Polydactyly	KIAA0586
300582	SHOX-Related Short Stature	SHOX
224410	Dyssegmental Dysplasia, Silverman-Handmaker type	HSPG2
258315	Omodysplasia 1	GPC6
258480	Opsismodysplasia	INPPL1
186500	Multiple Synostoses Syndrome 1	NOG
610017	Multiple Synostoses Syndrome 2	GDF5
612961	Multiple Synostoses Syndrome 3	FGF9
166300	Multicentric Carpotarsal Osteolysis Syndrome	MAFB
187600	Thanatophoric Dysplasia, type I	FGFR3
259600	Multicentric Osteolysis, Nodulosis, and Arthropathy	MMP2
147891	Small Patella Syndrome	TBX4
166260	Gnathodiaphyseal Dysplasia	ANO5
602361	Gracile Bone Dysplasia	FAM111A
150250	Autosomal Dominant Larsen Syndrome	FLNB
245600	Autosomal Recessive Larsen Syndrome	B3GAT3
114290	Campomelic Dysplasia	SOX9
131300	Camurati-Engelmann Disease	TGFB1
166350	Progressive Osseous Heteroplasia	GNAS
123000	Autosomal Dominant Craniometaphyseal Dysplasia	ANKH
118600	Chondrocalcinosis 2	ANKH
302950	X-linked chondrodysplasia punctata 1	ARSE
302960	X-linked chondrodysplasia punctata 2	EBP
242900	Schimke Immunoosseous Dysplasia	SMARCAL1
122860	Autosomal Dominant Craniodiaphyseal dysplasia	SOST
177170	Pseudoachondroplasia	COMP
265800	Pycnodysostosis	CTSK
215100	Rhizomelic Chondrodysplasia Punctata type 1	PEX7
222765	Rhizomelic Chondrodysplasia Punctata type 2	GNPAT
600121	Rhizomelic Chondrodysplasia Punctata type 3	AGPS
616716	Rhizomelic Chondrodysplasia Punctata type 5	PEX5
119600	Cleidocranial Dysplasia	RUNX2
193530	Weyers Acrofacial Dysostosis	EVC, EVC2
100800	Achondroplasia	FGFR3
146000	Hypochondroplasia	FGFR3
269250	Schneckenbecken Dysplasia	SLC35D1
248370	Mandibuloacral dysplasia with type A lipodystrophy	LMNA
608612	Mandibuloacral dysplasia with type B lipodystrophy	ZMPSTE24
167250	Paget disease of bone 3	SQSTM1
239000	Juvenile Paget Disease	TNFRSF11B
616833	Paget Disease Of Bone 6	ZNF687
276820	Limb pelvis hypoplasia aplasia syndrome	WNT7A
156500	Schmid Metaphyseal Chondrodysplasia	COL10A1
156400	Jansen metaphyseal chondrodysplasia	PTH1R
607944	Spondyloenchondrodysplasia with Immune Dysregulation	ACP5
239850	Hypertrichotic Osteochondrodysplasia	ABCC9
614078	Chondrodysplasia with Joint Dislocations, GRAPP type	IMPAD1
200700	Chondrodysplasia, Grebe type	GDF5
108720	Atelosteogenesis type I	FLNB
256050	Atelosteogenesis type II	SLC26A2
108721	Atelosteogenesis type III	FLNB
200600	Achondrogenesis type 1A	TRIP11
600972	Achondrogenesis type 1B	SLC26A2
200610	Achondrogenesis type 2	COL2A1
222600	Diastrophic Dysplasia	SLC26A2
132400	Multiple Epiphyseal Dysplasia 1	COMP
600204	Multiple Epiphyseal Dysplasia 2	COL9A2
600969	Multiple Epiphyseal Dysplasia 3	COL9A3
226900	Recessive Multiple Epiphyseal Dysplasia	SLC26A2
607078	Multiple Epiphyseal Dysplasia 5	MATN3
614135	Multiple Epiphyseal Dysplasia 6	COL9A1
226980	Multiple Epiphyseal Dysplasia with Early-Onset Diabetes Mellitus	EIF2AK3
608681	Spondylocostal dysostosis 2	MESP2
613686	Spondylocostal dysostosis 4	HES7
122600	Spondylocostal dysostosis 5	TBX6
616566	Spondylocostal dysostosis 6	RIPPLY2
183900	Spondyloepiphyseal Dysplasia Congenita	COL2A1
313400	X-Linked Spondyloepiphyseal Dysplasia Tarda	TRAPPC2
143095	Spondyloepiphyseal Dysplasia, Omani type	CHST3
616583	Spondyloepiphyseal Dysplasia, Stanescu Type	COL2A1
184095	Spondyloepiphyseal Dysplasia, Maroteaux type	TRPV4
184250	Spondyloepimetaphyseal Dysplasia, Strudwick type	COL2A1
603546	Spondyloepimetaphyseal Dysplasia with Joint Laxity type 2	KIF22
602111	Spondyloepimetaphyseal Dysplasia, Missouri type	MMP13
616723	Spondyloepimetaphyseal Dysplasia, Faden-Alkuraya Type	RSPRY1
610442	Spondyloepimetaphyseal Dysplasia, Genevieve Type	NANS
156250	Metachondromatosis	PTPN11
613073	Metaphyseal Anadysplasia 2	MMP9
156530	Metatropic Dysplasia	TRPV4
604864	Osteoarthritis with Mild Chondrodysplasia	COL2A1
608805	Primary Avascular Necrosis of Femoral Head 1	COL2A1
617383	Primary Avascular Necrosis of Femoral Head 2	TRPV4
101200	Apert syndrome	FGFR2
269500	Sclerosteosis 1	SOST
614305	Sclerosteosis 2	LRP4
607634	Autosomal Dominant Osteopetrosis 1	LRP5
166600	Autosomal Dominant Osteopetrosis 2	CLCN7
259700	Autosomal Recessive Osteopetrosis 1	TCIRG1
259710	Autosomal Recessive Osteopetrosis 2	TNFSF11
259730	Osteopetrosis with Renal Tubular Acidosis	CA2
611490	Autosomal Recessive Osteopetrosis 4	CLCN7
259720	Autosomal Recessive Osteopetrosis 5	OSTM1
611497	Autosomal Recessive Osteopetrosis 6	PLEKHM1
612301	Autosomal Recessive Osteopetrosis 7	TNFRSF11A
615085	Autosomal Recessive Osteopetrosis 8	SNX10
166200	Osteogenesis Imperfecta type I	COL1A1
166210	Osteogenesis Imperfecta type II	COL1A2, COL1A1
259420	Osteogenesis Imperfecta type III	COL1A2, COL1A1
166220	Osteogenesis Imperfecta type IV	COL1A2, COL1A1
610967	Osteogenesis Imperfecta type V	IFITM5
613982	Osteogenesis Imperfecta type VI	SERPINF1
610682	Osteogenesis Imperfecta type VII	CRTAP
610915	Osteogenesis Imperfecta type VIII	LEPRE1
259440	Osteogenesis Imperfecta type IX	PPIB
613848	Osteogenesis Imperfecta type X	SERPINH1
610968	Osteogenesis Imperfecta type XI	FKBP10
613849	Osteogenesis Imperfecta type XII	SP7
614856	Osteogenesis Imperfecta type XIII	BMP1
615066	Osteogenesis Imperfecta type XIV	TMEM38B
615220	Osteogenesis Imperfecta type XV	WNT1
616507	Osteogenesis imperfecta type XVII	SPARC
166250	Osteoglophonic Dysplasia	FGFR1
300373	Osteopathia Striata with Cranial Sclerosis	AMER1
215150	Otospondylomegaepiphyseal Dysplasia	COL11A2
151210	Platyspondylic Lethal Skeletal dysplasia, Torrance type	COL2A1
614185	Geleophysic dysplasia 2	FBN1
604757	Craniosynostosis 2	MSX2
615314	Craniosynostosis 3	TCF12
600775	Craniosynostosis 4	ERF
616602	Craniosynostosis 6	ZIC1
614188	Craniosynostosis and Dental Anomalies	IL11RA
123150	Jackson-Weiss Syndrome	FGFR1, FGFR2
241520	Autosomal Recessive Hypophosphatemic Rickets 1	DMP1
613312	Autosomal Recessive Hypophosphatemic Rickets 2	ENPP1
193100	Autosomal Dominant Hypophosphatemic Rickets	FGF23
241530	Hypophosphatemic Rickets with Hypercalciuria	SLC34A3
264700	Vitamin D-dependent rickets Type IA	CYP27B1
600081	Vitamin D-dependent rickets Type IB	CYP2R1
277440	Vitamin D-resistant Rickets Type IIA	VDR
616331	Robinow syndrome, autosomal dominant 2	DVL1
616894	Robinow syndrome, autosomal dominant 3	DVL3
616255	Short Stature With Nonspecific Skeletal Abnormalities	NPR2
168500	Parietal foramina 1	MSX2
609597	Parietal foramina 2	ALX4
218600	Baller-Gerold Syndrome	RECQL4
114000	Caffey Disease	COL1A1
607323	Duane-radial ray syndrome	SALL4
101400	Saethre-Chotzen Syndrome	TWIST1, FGFR2
118400	Cherubism	SH3BP2
133700	Hereditary Multiple Osteochondromatosis Type I	EXT1
133701	Hereditary Multiple Osteochondromatosis Type II	EXT2
215140	Greenberg dysplasia	LBR
112310	Boomerang dysplasia	FLNB
123500	Crouzon syndrome	FGFR2
612247	Crouzonodermoskeletal Syndrome	FGFR3
609162	Czech dysplasia	COL2A1
208230	Progressive Pseudorheumatoid Dysplasia	WISP3
266280	Rapadilino Syndrome	RECQL4
272460	Spondylocarpotarsal Synostosis Syndrome	FLNB
186570	Tarsal-Carpal Coalition Syndrome	NOG
185800	Proximal Symphalangism 1A	NOG
615298	Proximal Symphalangism 1B	GDF5
101800	Acrodysostosis 1, with or without Hormone Resistance	PRKAR1A
614613	Acrodysostosis 2, with or without Hormone Resistance	PDE4D
201250	Acromesomelic Dysplasia, Hunter-Thompson Type	GDF5
602875	Acromesomelic Dysplasia, Maroteaux Type	NPR2
102370	Acromicric Dysplasia	FBN1
602483	Auriculocondylar Syndrome 1	GNAI3
614669	Auriculocondylar Syndrome 2	PLCB4
615706	Auriculocondylar Syndrome 3	EDN1
112500	Brachydactyly Type A1	IHH
616849	Brachydactyly Type A1,D	BMPR1B
112600	Brachydactyly Type A2	GDF5, BMPR1B, BMP2
113000	Brachydactyly Type B1	ROR2
611377	Brachydactyly Type B2	NOG
113100	Brachydactyly Type C	GDF5
613382	Brachydactyly Type E2	PTHLH
615072	Brachydactyly Type A1,C	GDF5
156510	Metaphyseal Dysplasia with Maxillary Hypoplasia with or without Brachydactyly	RUNX2
228900	Fibular Hypoplasia and Complex Brachydactyly	GDF5
606835	Familial Digital Arthropathy-Brachydactyly	TRPV4
113500	Brachyolmia Type 3	TRPV4
612847	Brachyolmia Type 4	PAPSS2
259450	Bruck Syndrome 1	FKBP10
609220	Bruck Syndrome 2	PLOD2
608940	Spondylometaphyseal Dysplasia with Cone-Rod Dystrophy	PCYT1A
192950	Congenital Vertical Talus	HOXD10
218330	Cranioectodermal Dysplasia 1	IFT122
613610	Cranioectodermal Dysplasia 2	WDR35
614099	Cranioectodermal Dysplasia 3	IFT43
614378	Cranioectodermal Dysplasia 4	WDR19
180849	Rubinstein-Taybi Syndrome 1	CREBBP
613684	Rubinstein-Taybi Syndrome 2	EP300
251450	Desbuquois Dysplasia 1	CANT1
608022	Diaphanospondylodysostosis	BMPER
223800	Dyggve-Melchior-Clausen Disease	DYM
277590	Weaver Syndrome	EZH2
228520	Fibrochondrogenesis 1	COL11A1
614524	Fibrochondrogenesis 2	COL11A2
135100	Fibrodysplasia Ossificans Progressiva	ACVR1
228930	Fuhrmann Syndrome	WNT7A
136760	Frontonasal Dysplasia 1	ALX3
613451	Frontonasal Dysplasia 2	ALX4
613456	Frontonasal Dysplasia 3	ALX1
102500	Hajdu-Cheney Syndrome	NOTCH2
144750	Endosteal Hyperostosis	LRP5
119900	Isolated Congenital Digital Clubbing	HPGD
245150	Keutel Syndrome	MGP
118100	Klippel-Feil Syndrome 1	GDF6
214300	Klippel-Feil Syndrome 2	MEOX1
613702	Klippel-Feil Syndrome 3	GDF3
616549	Klippel-Feil Syndrome 4	MYO18B
249700	Langer Mesomelic Dwarfism	SHOX
127300	Madelung deformity	SHOX
210720	Microcephalic Osteodysplastic Primordial Dwarfism, Type II	PCNT
154400	Nager Syndrome	SF3B4
101600	Pfeiffer Syndrome	FGFR1, FGFR2
614441	Primary Hypertrophic Osteoarthropathy	SLCO2A1
259775	Raine Syndrome	FAM20C
608355	Parkes Weber Syndrome	RASA1
255800	Schwartz-Jampel Syndrome, Type 1	HSPG2
607326	Smith-McCort Dysplasia 1	DYM
615222	Smith-McCort Dysplasia 2	RAB33B
186100	Syndactyly, Type III	GJA1
186200	Syndactyly, Type IV	LMBR1
212780	Cenani-Lenz Syndactyly Syndrome	LRP4
174500	Triphalangeal Thumb-Polysyndactyly Syndrome	LMBR1
300707	Toe Syndactyly, Telecanthus, and Anogenital and Renal Malformations	FAM58A
174700	Preaxial Polydactyly Type IV	GLI3
239100	Van Buchem Disease	SOST
607636	Van Buchem Disease, Type 2	LRP5
277950	Winchester syndrome	MMP14
614592	Bent Bone Dysplasia Syndrome	FGFR2
119800	Clubfoot, Congenital, With Or Without Deficiency Of Long Bones And/Or mirror-Image Polydactyly	PITX1
607778	Acrocapitofemoral Dysplasia	IHH
211800	Calcification Of Joints And Arteries	NT5E
609441	Acromesomelic Chondrodysplasia With Genital Anomalies	BMPR1B
215045	Chondrodysplasia, Blomstrand Type	PTH1R
218400	Craniometaphyseal Dysplasia, Autosomal Recessive	GJA1
615923	Epiphyseal Chondrodysplasia, Miura Type	NPR2
132450	Epiphyseal Dysplasia, Multiple, With Myopia And Conductive Deafness	COL2A1
174810	Familial Expansile Osteolysis	TNFRSF11A
259100	Hypertrophic Osteoarthropathy, Primary, Autosomal Recessive, 1	HPGD
300554	X-Linked Recessive Hypophosphatemic Rickets	CLCN5
150600	Legg-Calve-Perthes Disease	COL2A1
151050	Lenz-Majewski Hyperostotic Dwarfism	PTDSS1
186550	Liebenberg Syndrome	PITX1
309630	Metacarpal 4-5 Fusion	FGF16
140600	Osteoarthritis Susceptibility 2	MATN3
168550	Parietal Foramina With Cleidocranial Dysplasia	MSX2
174200	Polydactyly, Postaxial, Type A1	GLI3
615226	Polydactyly, Postaxial, Type A6	ZNF141
614416	Radiohumeral Fusions With Other Skeletal And Craniofacial Anomalies	CYP26B1
615709	Sacral Agenesis With Vertebral Anomalies	T
147250	Solitary Median Maxillary Central Incisor	SHH
608728	Spondyloepimetaphyseal Dysplasia, Matrilin-3 Related	MATN3
184252	Spondylometaphyseal Dysplasia, Kozlowski Type	TRPV4
613320	Spondylometaphyseal Dysplasia, Megarbane-Dagher-Melki Type	PAM16
250220	Spondylometaphyseal Dysplasia, Sedaghatian Type	GPX4
271700	Spondyloperipheral Dysplasia	COL2A1
615155	Steel Syndrome	COL27A1
608180	Synpolydactyly 2	FBLN1
188740	Hypoplasia Or Aplasia Of Tibia With Polydactyly	LMBR1
600920	Van Den Ende-Gupta Syndrome	SCARF2
142669	Hip dysplasia, Beukes type	UFSP2
616897	Complex Lethal Osteochondrodysplasia, Symoens-Barnes-Gistelinck Type	TAPT1
616890	Split-Foot Malformation With Mesoaxial Polydactyly	ZAK
617602	Congenital Heart Defects And Skeletal Malformations Syndrome	ABL1
244400	Primary Ciliary Dyskinesia 1	DNAI1
606763	Primary Ciliary Dyskinesia 2	DNAAF3
608644	Primary Ciliary Dyskinesia 3	DNAH5
610852	Primary Ciliary Dyskinesia 6	NME8
611884	Primary Ciliary Dyskinesia 7	DNAH11
612444	Primary Ciliary Dyskinesia 9	DNAI2
612518	Primary Ciliary Dyskinesia 10	DNAAF2
612649	Primary Ciliary Dyskinesia 11	RSPH4A
612650	Primary Ciliary Dyskinesia 12	RSPH9
613193	Primary Ciliary Dyskinesia 13	DNAAF1
613807	Primary Ciliary Dyskinesia 14	CCDC39
613808	Primary Ciliary Dyskinesia 15	CCDC40
614017	Primary Ciliary Dyskinesia 16	DNAL1
614679	Primary Ciliary Dyskinesia 17	CCDC103
614935	Primary Ciliary Dyskinesia 19	LRRC6
615067	Primary Ciliary Dyskinesia 20	CCDC114
615294	Primary Ciliary Dyskinesia 21	DRC1
615444	Primary Ciliary Dyskinesia 22	ZMYND10
615451	Primary Ciliary Dyskinesia 23	ARMC4
615481	Primary Ciliary Dyskinesia 24	RSPH1
615482	Primary Ciliary Dyskinesia 25	DYX1C1
615500	Primary Ciliary Dyskinesia 26	C21orf59
615504	Primary Ciliary Dyskinesia 27	CCDC65
615505	Primary Ciliary Dyskinesia 28	SPAG1
615872	Primary Ciliary Dyskinesia 29	CCNO
616037	Primary Ciliary Dyskinesia 30	CCDC151
616369	Primary Ciliary Dyskinesia 31	CENPF
616481	Primary Ciliary Dyskinesia 32	RSPH3
616726	Primary Ciliary Dyskinesia 33	GAS8
219700	Cystic Fibrosis	CFTR
178600	Primary Pulmonary Hypertension-1	BMPR2
615342	Primary Pulmonary Hypertension-2	SMAD9
615343	Primary Pulmonary Hypertension-3	CAV1
615344	Primary Pulmonary Hypertension-4	KCNK3
601200	Pleuropulmonary blastoma	DICER1
173600	Primary Spontaneous Pneumothorax	FLCN
209880	Congenital Central Hypoventilation Syndrome	PHOX2B
610187	Diaphragmatic Hernia 3	ZFPM2
178500	Familial Idiopathic Pulmonary Fibrosis	SFTPA2
265100	Pulmonary Alveolar Microlithiasis	SLC34A2
NA043	Mucociliary Clearance Disorder	MCIDAS
303600	Coffin-Lowry Syndrome	RPS6KA3
600274	Frontotemporal Dementia	PSEN1, MAPT
127750	Dementia with Lewy Bodies	SNCA, SNCB
607485	GRN-Related Frontotemporal Dementia	GRN
600072	Fatal Familial Insomnia	PRNP
123400	Familial Creutzfeldt-Jakob Disease	PRNP
300322	Lesch-Nyhan Syndrome	HPRT1
300260	MECP2 Duplication Syndrome	MECP2
309520	Lujan-Fryns syndrome	MED12
168601	Parkinson Disease 1	SNCA
600116	Parkinson Disease 2	PARK2
605543	Parkinson Disease 4	SNCA
605909	Parkinson Disease 6	PINK1
606324	Parkinson Disease 7	PARK7
612953	Parkinson Disease 14	PLA2G6
260300	Parkinson Disease 15	FBXO7
614251	Parkinson Disease 18	EIF4G1
615528	Parkinson Disease 19	DNAJC6
615530	Parkinson Disease 20	SYNJ1
616361	Parkinson Disease 21	DNAJC13
616710	Parkinson Disease 22	CHCHD2
616840	Parkinson Disease 23	VPS13C
260540	Parkinson-Dementia Syndrome	MAPT
300911	X-Linked Parkinsonism With Spasticity	ATP6AP2
616859	Childhood-Onset Spasticity With Hyperglycinemia	GLRX5
604348	Familial Advanced Sleep Phase Syndrome 1	PER2
615224	Familial Advanced Sleep Phase Syndrome 2	CSNK1D
616882	Familial Advanced Sleep Phase Syndrome 3	PER3
137580	Tourette Syndrome	SLITRK1
118700	Benign Hereditary Chorea	NKX2-1
605309	Macrocephaly/autism syndrome	PTEN
300624	Fragile X syndrome	FMR1
104310	Alzheimer Disease 2	APOE
607822	Alzheimer Disease 3	PSEN1
606889	Alzheimer Disease 4	PSEN2
614306	Cognitive Impairment With Or Without Cerebellar Ataxia	SCN8A
157600	Mirror Movements 1	DCC
614508	Mirror Movements 2	RAD51
616059	Mirror Movements 3	DNAL4
161400	Narcolepsy 1	HCRT
614250	Narcolepsy 7	MOG
164230	Obsessive-Compulsive Disorder	BDNF, HTR2A, SLC6A4
172700	Pick Disease Of Brain	MAPT
612975	Short Sleeper	BHLHE41
615432	Specific Language Impairment 5	TM4SF20
613229	Trichotillomania	SLITRK1
184450	Familial Persistent Stuttering 1	AP4E1
616939	Childhood-Onset Chorea With Psychomotor Retardation	GPR88
616839	Riboflavin-Responsive Exercise Intolerance	SLC25A32
NA030	Genetic Prion Diseases	PRNP
104530	Amelogenesis imperfecta, type IA	LAMB3
104500	Amelogenesis imperfecta, type IB	ENAM
204650	Amelogenesis imperfecta, type IC	ENAM
301200	Amelogenesis imperfecta, type IE	AMELX
616270	Amelogenesis imperfecta, type IF	AMBN
204690	Amelogenesis imperfecta, type IG	FAM20A
616221	Amelogenesis imperfecta, type IH	ITGB6
204700	Amelogenesis imperfecta, type IIA1	KLK4
612529	Amelogenesis imperfecta, type IIA2	MMP20
613211	Amelogenesis imperfecta, type IIA3	WDR72
614832	Amelogenesis imperfecta, type IIA4	C4orf26
615887	Amelogenesis imperfecta, type IIA5	SLC24A4
130900	Amelogenesis Imperfecta, Type III	FAM83H
104510	Amelogenesis Imperfecta, Type IV	DLX3
125400	Dentin Dysplasia, Type I	SMOC2
125420	Dentin Dysplasia, Type II	DSPP
125490	Dentinogenesis Imperfecta 1	DSPP
125500	Dentinogenesis Imperfecta, Shields Type III	DSPP
135300	Gingival Fibromatosis 1	SOS1
106600	Selective Tooth Agenesis 1	MSX1
604625	Selective Tooth Agenesis 3	PAX9
150400	Selective Tooth Agenesis 4	WNT10A
601216	Selective Tooth Agenesis 6	LTBP3
616724	Selective Tooth Agenesis 7	LRP6
617073	Selective Tooth Agenesis 8	WNT10B
313500	X-Linked Selective Tooth Agenesis 1	EDA
193900	White Sponge Nevus 1	KRT4
615785	White Sponge Nevus 2	KRT13
189500	Witkop Syndrome	MSX1
125350	Primary Failure Of Tooth Eruption	PTH1R
170650	Aggressive Periodontitis 1	CTSC
612286	Hypophosphatemic Nephrolithiasis/osteoporosis 1	SLC34A1
300009	Dent Disease 1	CLCN5
300555	Dent Disease 2	OCRL
310468	X-Linked Recessive Nephrolithiasis with Renal Failure	CLCN5
601678	Bartter Syndrome 1	SLC12A1
241200	Bartter Syndrome 2	KCNJ1
607364	Bartter Syndrome 3	CLCNKB
602522	Bartter Syndrome 4A	BSND
613090	Digenic Bartter Syndrome 4B	CLCNKA, CLCNKB
300971	Bartter Syndrome 5	MAGED2
173900	Polycystic kidney disease 1	PKD1
174000	Medullary cystic kidney disease type 1	MUC1
603860	Medullary cystic kidney disease type 2	UMOD
263200	Autosomal Recessive Polycystic Kidney Disease	PKHD1
194080	Denys-Drash syndrome	WT1
613092	Familial Juvenile Hyperuricemic Nephropathy Type 2	REN
136680	Frasier syndrome	WT1
267430	Renal Tubular Dysgenesis	ACE, AGT, AGTR1, REN
267300	Distal Renal Tubular Acidosis with Progressive Nerve Deafness	ATP6V1B1
104200	Autosomal Dominant Alport Syndrome	COL4A3
301050	X-linked Alport Syndrome	COL4A5
203780	Autosomal Recessive Alport Syndrome	COL4A3, COL4A4
615008	Nephrotic Syndrome Type 7	DGKE
614809	Nephropathy due to CFHR5 deficiency	CFHR5
162000	Familial Juvenile Hyperuricemic Nephropathy Type 1	UMOD
153640	Fechtner Syndrome	MYH9
603278	Focal Segmental Glomerulosclerosis 1	ACTN4
603965	Focal Segmental Glomerulosclerosis 2	TRPC6
607832	Focal Segmental Glomerulosclerosis 3	CD2AP
613237	Focal Segmental Glomerulosclerosis 5	INF2
614131	Focal Segmental Glomerulosclerosis 6	MYO1E
616002	Focal Segmental Glomerulosclerosis 7	PAX2
616032	Focal Segmental Glomerulosclerosis 8	ANLN
616220	Focal Segmental Glomerulosclerosis 9	CRB2
611590	Distal Renal Tubular Acidosis with Hemolytic Anemia	SLC4A1
179800	Autosomal Dominant Distal Renal Tubular Acidosis	SLC4A1
602722	Autosomal Recessive Distal Renal Tubular Acidosis	ATP6V0A4
604278	Proximal Renal Tubular Acidosis with Ocular Abnormalities	SLC4A4
614817	Karyomegalic Interstitial Nephritis	FAN1
236730	Urofacial Syndrome 1	HPSE2
615112	Urofacial Syndrome 2	LRIG2
300539	Nephrogenic Syndrome of Inappropriate Antidiuresis	AVPR2
256100	Nephronophthisis 1	NPHP1
602088	Nephronophthisis 2	INVS
604387	Nephronophthisis 3	NPHP3
606966	Nephronophthisis 4	NPHP4
611498	Nephronophthisis 7	GLIS2
613824	Nephronophthisis 9	NEK8
613550	Nephronophthisis 11	TMEM67
614377	Nephronophthisis 13	WDR19
614845	Nephronophthisis 15	CEP164
615862	Nephronophthisis 18	CEP83
616217	Nephronophthisis 19	DCDC2
613159	Nephronophthisis-Like Nephropathy 1	XPNPEP3
609057	Nephropathy with Pretibial Epidermolysis Bullosa and Deafness	CD151
256300	Nephrotic Syndrome Type 1	NPHS1
600995	Nephrotic Syndrome Type 2	NPHS2
610725	Nephrotic Syndrome Type 3	PLCE1
256370	Nephrotic Syndrome Type 4	WT1
614199	Nephrotic Syndrome, Type 5, with or without Ocular Abnormalities	LAMB2
614196	Nephrotic Syndrome Type 6	PTPRO
615244	Nephrotic Syndrome Type 8	ARHGDIA
615573	Nephrotic Syndrome Type 9	ADCK4
615861	Nephrotic Syndrome Type 10	EMP2
616730	Nephrotic Syndrome Type 11	NUP107
616892	Nephrotic Syndrome Type 12	NUP93
616893	Nephrotic Syndrome Type 13	NUP205
615399	Paroxysmal Nocturnal Hemoglobinuria 2	PIGT
261550	Persistent Mullerian Duct Syndrome, Type I and II	AMH, AMHR2
609049	Pierson Syndrome	LAMB2
233100	Renal Glucosuria	SLC5A2
220150	Renal Hypouricemia 1	SLC22A12
612076	Renal Hypouricemia 2	SLC2A9
191830	Renal Hypodysplasia/aplasia-1	ITGA8
615721	Renal Hypodysplasia/aplasia-2	FGF20
268200	Acute Recurrent Myoglobinuria	LPIN1
613388	Fanconi Renotubular Syndrome 2	SLC34A1
615605	Fanconi Renotubular Syndrome 3	EHHADH
616026	Fanconi renotubular syndrome 4 with maturity-onset diabetes of the young	HNF4A
100100	Abdominal Muscles, Absence Of, With Urinary Tract Abnormality And cryptorchidism	CHRM3
219750	Cystinosis, Adult Nonnephropathic	CTNS
219900	Cystinosis, Late-Onset Juvenile Or Adolescent Nephropathic Type	CTNS
134610	Autosomal Dominant Familial Mediterranean Fever	MEFV
609886	Glomerulocystic Kidney Disease With Hyperuricemia And Isosthenuria	UMOD
601894	Glomerulopathy With Fibronectin Deposits 2	FN1
141200	Benign Familial Hematuria	COL4A3, COL4A4
143870	Absorptive Hypercalciuria 2	ADCY10
242600	Iminoglycinuria	SLC36A2
611771	Lipoprotein Glomerulopathy	APOE
308990	Proteinuria, Low Molecular Weight, With Hypercalciuria And Nephrocalcinosis	CLCN5
615415	Renal-Hepatic-Pancreatic Dysplasia 2	NEK8
143400	Congenital Anomalies Of Kidney And Urinary Tract 2	TBX18
NA018	Nephronophthisis 8	RPGRIP1L
NA019	Immunoglobulin-mediated membranoproliferative glomerulonephritis	CFH
NA045	UPK3A-Related Renal Adysplasia	UPK3A
266900	Senior-Loken Syndrome 1	NPHP1
606996	Senior-Loken Syndrome 4	NPHP4
609254	Senior-Loken syndrome 5	IQCB1
610189	Senior-Loken Syndrome 6	CEP290
613615	Senior-Loken Syndrome 7	SDCCAG8
616307	Senior-Loken Syndrome 8	WDR19
616629	Senior-Loken Syndrome 9	TRAF3IP1
607594	Common Variable Immune Deficiency 1	ICOS
240500	Common Variable Immune Deficiency 2	TNFRSF13B
613493	Common Variable Immune Deficiency 3	CD19
613495	Common Variable Immune Deficiency 5	MS4A1
613496	Common Variable Immune Deficiency 6	CD81
614699	Common Variable Immune Deficiency 7	CR2
614700	Common Variable Immune Deficiency 8 with Autoimmunity	LRBA
615577	Common Variable Immune Deficiency 10	NFKB2
615767	Common Variable Immune Deficiency 11	IL21
616576	Common Variable Immune Deficiency 12	NFKB1
616873	Common Variable Immune Deficiency 13	IKZF1
102700	Adenosine Deaminase Deficiency	ADA
601457	RAG1-Related Severe Combined Immunodeficiency	RAG1, RAG2
602450	DCLRE1C-Related Severe Combined Immunodeficiency	DCLRE1C
603554	Omenn Syndrome	RAG1, RAG2, DCLRE1C
608971	Severe combined immunodeficiency, T cell-negative, B-cell/natural killer-cell positive	PTPRC, IL7R
312863	X-Linked Combined Immunodeficiency	IL2RG
600802	JAK3-Related Related Severe Combined Immunodeficiency	JAK3
235550	Hepatic Veno-Occlusive Disease with Immunodeficiency	SP110
606593	DNA ligase IV deficiency	LIG4
613179	Purine Nucleoside Phosphorylase Deficiency	PNP
267500	Reticular Dysgenesis	AK2
300755	X-Linked Agammaglobulinemia 1	BTK
601495	Agammaglobulinemia 1	IGHM
613500	Agammaglobulinemia 2	IGLL1
613501	Agammaglobulinemia 3	CD79A
613502	Agammaglobulinemia 4	BLNK
613506	Agammaglobulinemia 5	LRRC8A
612692	Agammaglobulinemia 6	CD79B
615214	Agammaglobulinemia 7	PIK3R1
616941	Agammaglobulinemia 8	TCF3
608203	Neutrophil immunodeficiency syndrome	RAC2
613860	Immunodeficiency due to ficolin 3 deficiency	FCN3
610798	Immunodeficiency due to defect in MAPBP-interacting protein	LAMTOR2
300400	X-Linked Severe Combined Immunodeficiency	IL2RG
615387	Immunodeficiency 7	TRAC
612782	Immunodeficiency 9	ORAI1
612783	Immunodeficiency 10	STIM1
615206	Immunodeficiency 11	CARD11
615468	Immunodeficiency 12	MALT1
615518	Immunodeficiency 13	UNC119
615513	Immunodeficiency 14	PIK3CD
615592	Immunodeficiency 15	IKBKB
615593	Immunodeficiency 16	TNFRSF4
615607	Immunodeficiency 17	CD3G
615615	Immunodeficiency 18	CD3E
615617	Immunodeficiency 19	CD3D
615707	Immunodeficiency 20	FCGR3A
614172	Immunodeficiency 21	GATA2
615758	Immunodeficiency 22	LCK
615816	Immunodeficiency 23	PGM3
615897	Immunodeficiency 24	CTPS1
610163	Immunodeficiency 25	CD247
615966	Immunodeficiency 26	PRKDC
209950	Immunodeficiency 27A	IFNGR1
615978	Immunodeficiency 27B	IFNGR1
614890	Immunodeficiency 29	IL12B
614891	Immunodeficiency 30	IL12RB1
614892	Immunodeficiency 31A	STAT1
613796	Immunodeficiency 31B	STAT1
614162	Familial Candidiasis 7(Immunodeficiency 31)	STAT1
614893	Immunodeficiency 32A	IRF8
614894	Immunodeficiency 32B, monocyte and dendritic cell deficiency, autosomal recessive	IRF8
300645	Immunodeficiency 34	CYBB
611521	Immunodeficiency 35	TYK2
616005	Immunodeficiency 36	PIK3R1
616098	Immunodeficiency 37	BCL10
616126	Immunodeficiency 38, With Basal Ganglia Calcification	ISG15
616345	Immunodeficiency 39	IRF7
616433	Immunodeficiency 40	DOCK2
606367	Immunodeficiency 41 with lymphoproliferation and autoimmunity	IL2RA
616622	Immunodeficiency 42	RORC
241600	Immunodeficiency 43	B2M
616636	Immunodeficiency 44	STAT2
616669	Immunodeficiency 45	IFNAR2
616740	Immunodeficiency 46	TFRC
300972	Immunodeficiency 47	ATP6AP1
269840	Immunodeficiency 48	ZAP70
613953	Familial Candidiasis 5	IL17RA
242860	Immunodeficiency-centromeric instability-facial anomalies syndrome 1	DNMT3B
614069	Immunodeficiency-Centromeric Instability-Facial Anomalies Syndrome 2	ZBTB24
616910	Immunodeficiency-Centromeric Instability-Facial Anomalies Syndrome 3	CDCA7
616911	Immunodeficiency-Centromeric Instability-Facial Anomalies Syndrome 4	HELLS
615207	Il21R Immunodeficiency	IL21R
300853	X-Linked Immunodeficiency with Magnesium Defect, Epstein-Barr Virus Infection, and Neoplasia	MAGT1
614868	T-Cell Immunodeficiency, Recurrent Infections, Autoimmunity and Cardiac Malformations	STK4
611291	Severe Combined Immunodeficiency with Microcephaly, Growth Retardation, and Sensitivity to Ionizing Radiation	NHEJ1
202700	Severe Congenital Neutropenia, Autosomal Dominant,1	ELANE
613107	Severe Congenital Neutropenia, Autosomal Dominant,2	GFI1
610738	Severe Congenital Neutropenia, Autosomal Recessive,3	HAX1
612541	Severe Congenital Neutropenia, Autosomal Recessive,4	G6PC3
615285	Severe Congenital Neutropenia, Autosomal Recessive,5	VPS45
616022	Severe Congenital Neutropenia, Autosomal Recessive,6	JAGN1
617014	Severe Congenital Neutropenia, Autosomal Recessive,7	CSF3R
308230	Immunodeficiency with Hyper-IgM, type 1	CD40LG
605258	Immunodeficiency with Hyper-IgM, type 2	AICDA
606843	Immunodeficiency with Hyper-IgM, type 3	CD40
608106	Immunodeficiency with Hyper-IgM, type 5	UNG
245590	Growth Hormone Insensitivity with Immunodeficiency	STAT5B
612132	Anhidrotic Ectodermal Dysplasia with T-cell Immunodeficiency	NFKBIA
601705	Congenital alopecia and T-Cell Immunodeficiency and nail dystrophy	FOXN1
613011	Lymphoproliferative Syndrome 1	ITK
615122	Lymphoproliferative Syndrome 2	CD27
308240	X-Linked Lymphoproliferative syndrome 1	SH2D1A
300635	X-Linked Lymphoproliferative syndrome 2	XIAP
601859	Autoimmune lymphoproliferative syndrome type I	FAS, FASLG
603909	Autoimmune lymphoproliferative syndrome type IIA	CASP10
607271	Autoimmune Lymphoproliferative Syndrome, Type IIB	CASP8
615559	Autoimmune Lymphoproliferative Syndrome, type III	PRKCD
614470	Autoimmune Lymphoproliferative Syndrome, Type IV	NRAS
616100	Autoimmune Lymphoproliferative Syndrome, type V	CTLA4
613075	Macrocephaly, Alopecia, Cutis Laxa, and Scoliosis(MACS syndrome)	RIN2
603553	Familial Hemophagocytic Lymphohistiocytosis 2	PRF1
608898	Familial Hemophagocytic Lymphohistiocytosis 3	UNC13D
603552	Familial Hemophagocytic Lymphohistiocytosis 4	STX11
613101	Familial Hemophagocytic Lymphohistiocytosis 5	STXBP2
249100	Familial Mediterranean Fever	MEFV
147060	Autosomal Dominant Hyper IgE Syndrome	STAT3
300299	X-Linked Severe Congenital Neutropenia	WAS
304790	IPEX Syndrome	FOXP3
609628	Majeed Syndrome	LPIN2
228000	Farber Lipogranulomatosis	ASAH1
186580	Blau syndrome	NOD2
607676	IRAK4 deficiency	IRAK4
142680	Tumor Necrosis Factor Receptor-associated Periodic Syndrome	TNFRSF1A
208900	Ataxia-telangiectasia	ATM
233690	Autosomal Recessive Cytochrome B-Negative Chronic Granulomatous Disease	CYBA
233710	Autosomal Recessive Cytochrome B-Positive Chronic Granulomatous Disease Type II	NCF2
613960	Autosomal Recessive Cytochrome B-Positive Chronic Granulomatous Disease Type III	NCF4
306400	X-linked Chronic Granulomatous Disease	CYBB
604571	Bare Lymphocyte Syndrome, Type I	TAPBP, TAP1, TAP2
209920	Bare Lymphocyte Syndrome, Type II	RFX5, RFXANK, RFXAP, CIITA
243700	Hyper IgE Syndrome	DOCK8
613779	C3 Deficiency	C3
609536	C5 Deficiency	C5
613783	Complement Component C1S Deficiency	C1S
217000	Complement Component C2 Deficiency	C2
120790	Complement Component 4, Partial Deficiency Of	SERPING1
612446	Complement Component 6 Deficiency	C6
610102	Complement Component 7 Deficiency	C7
613790	Complement Component 8 Deficiency, Type I	C8A
613789	Complement Component 8 Deficiency, Type II	C8B
613825	Complement Component 9 Deficiency	C9
615561	Complement Factor B Deficiency	CFB
613912	Complement Factor D Deficiency	CFD
609814	Complement Factor H Deficiency	CFH
212050	Familial Candidiasis 2	CARD9
613108	Familial Candidiasis 4	CLEC7A
613956	Familial Candidiasis 6	IL17F
615527	Familial Candidiasis 8	TRAF3IP2
616445	Familial Candidiasis 9	IL17RC
120100	Familial Cold Autoinflammatory Syndrome 1	NLRP3
611762	Familial Cold Autoinflammatory Syndrome 2	NLRP12
614468	Familial Cold Autoinflammatory Syndrome 3	PLCG2
616115	Familial Cold Autoinflammatory Syndrome 4	NLRC4
616744	Familial Behcet-like autoinflammatory syndrome	TNFAIP3
260920	Hyper IgD Syndrome	MVK
612852	Interleukin 1 Receptor Antagonist Deficiency	IL1RN
116920	Leukocyte Adhesion Deficiency type 1	ITGB2
612840	Leukocyte Adhesion Deficiency type 3	FERMT3
613791	MASP2 Deficiency	MASP2
614038	Primary Lymphedema with Myelodysplasia	GATA2
312060	X-Linked Properdin Deficiency	CFP
612260	Recurrent Pyogenic Bacterial Infections due to MYD88 Deficiency	MYD88
193670	WHIM Syndrome	CXCR4
301000	Wiskott-Aldrich Syndrome 1	WAS
614493	Wiskott-Aldrich Syndrome 2	WIPF1
609889	Alpha/Beta T-Cell Lymphopenia With Gamma/Delta T-Cell Expansion, Severe cytomegalovirus infection, and autoimmunity	RAG1
615952	Infantile-Onset Multisystem Autoimmune Disease 1	STAT3
617006	Infantile-Onset Multisystem Autoimmune Disease 2	ZAP70
613385	Multisystem Autoimmune Disease With Facial Dysmorphism	ITCH
616050	Autoinflammation With Infantile Enterocolitis	NLRC4
614878	PLCG2-Associated Antibody Deficiency Autoinflammation And Immune Dysregulation	PLCG2
608957	Familial CD8 Deficiency	CD8A
233650	Combined Cellular And Humoral Immune Defects With Granulomas	RAG1, RAG2
147050	Atopic IgE Responsiveness	SPINK5
609529	Immunoglobulin A Deficiency 2	TNFRSF13B
614102	Immunoglobulin Kappa Light Chain Deficiency	IGKC
614328	Inflammatory Skin And Bowel Disease, Neonatal, 1	ADAM17
616069	Inflammatory Skin And Bowel Disease, Neonatal, 2	EGFR
254600	Myeloperoxidase Deficiency	MPO
245480	Specific Granule Deficiency 1	CEBPE
614420	Systemic Lupus Erythematosus 16	DNASE1L3
162800	Cyclic Neutropenia	ELANE
616452	B-cell expansion with NFKB and T-cell anergy	CARD11
617388	Autoinflammation with arthritis and dyskeratosis	NLRP1
131100	Multiple Endocrine Neoplasia type 1	MEN1
171400	Multiple Endocrine Neoplasia type 2A	RET
162300	Multiple Endocrine Neoplasia type 2B	RET
610755	Multiple Endocrine Neoplasia type 4	CDKN1B
613038	Combined pituitary hormone deficiency 1	POU1F1
262600	Combined pituitary hormone deficiency 2	PROP1
221750	Combined pituitary hormone deficiency 3	LHX3
262700	Combined pituitary hormone deficiency 4	LHX4
182230	Septo-Optic Dysplasia	HESX1
613986	Combined pituitary hormone deficiency 6	OTX2
615849	Culler-Jones syndrome	GLI2
308700	Kallmann Syndrome 1	KAL1
147950	Kallmann Syndrome 2	FGFR1
244200	Kallmann Syndrome 3	PROKR2
612370	Kallmann Syndrome 5	CHD7
612702	Kallmann Syndrome 6	FGF8
146110	Hypogonadotropic Hypogonadism 7 With Or Without Anosmia	GNRHR
614837	Hypogonadotropic hypogonadism 8	KISS1R
614838	Hypogonadotropic Hypogonadism 9 with or without Anosmia	NSMF
614839	Hypogonadotropic hypogonadism 10	TAC3
614840	Hypogonadotropic hypogonadism 11	TACR3
614841	Hypogonadotropic hypogonadism 12	GNRH1
614842	Hypogonadotropic hypogonadism 13	KISS1
614858	Hypogonadotropic hypogonadism 14	WDR11
615266	Hypogonadotropic hypogonadism 17	SPRY4
615267	Hypogonadotropic hypogonadism 18	IL17RD
615269	Hypogonadotropic hypogonadism 19	DUSP6
615270	Hypogonadotropic hypogonadism 20	FGF17
615271	Hypogonadotropic hypogonadism 21	FLRT3
616030	Hypogonadotropic hypogonadism with or without anosmia 22	FEZF1
228300	Hypogonadotropic hypogonadism with or without anosmia 23	LHB
229070	Hypogonadotropic hypogonadism with or without anosmia 24	FSHB
607694	Hypomyelinating Leukodystrophy 7 with or without oligodontia and/or hypogonadotropic hypogonadism	POLR3A
102200	Familial Isolated Pituitary Adenomas	AIP
300943	Pituitary Adenoma, Growth Hormone-Secreting, 2	GPR101
600634	Pituitary Adenoma, Prolactin-Secreting	AIP
300068	Androgen Insensitivity Syndrome	AR
300200	X-Linked Adrenal Hypoplasia Congenita	NR0B1
240300	Autoimmune Polyendocrine Syndrome Type 1	AIRE
145000	Hyperparathyroidism 1	CDC73
145001	Hyperparathyroidism 2	CDC73
617343	Hyperparathyroidism 4	GCM2
262400	Isolated growth hormone deficiency type IA	GH1
612781	Isolated growth hormone deficiency type IB	GH1,GHRHR
173100	Isolated growth hormone deficiency type II	GH1
307200	Isolated growth hormone deficiency type III	BTK
615925	Partial Isolated Growth Hormone Deficiency	GHSR
256450	Familial Hyperinsulinemic Hypoglycemia 1	ABCC8
601820	Familial Hyperinsulinemic Hypoglycemia 2	KCNJ11
602485	Familial Hyperinsulinemic Hypoglycemia 3	GCK
609975	Familial Hyperinsulinemic Hypoglycemia 4	HADH
606762	Familial Hyperinsulinemic Hypoglycemia 6	GLUD1
610021	Familial Hyperinsulinemic Hypoglycemia 7	SLC16A1
262190	Pineal Hyperplasia, Insulin-Resistant Diabetes Mellitus, and Somatic Abnormalities	INSR
125852	Diabetes Mellitus, Insulin-Dependent, 2	INS
612520	Diabetes Mellitus, Insulin-Dependent, 20	HNF1A
610549	Diabetes Mellitus, Insulin-Resistant, With Acanthosis Nigricans	INSR
612227	Diabetes Mellitus, Ketosis-Prone	PAX4
610374	Diabetes Mellitus, Transient Neonatal 2	ABCC8
610582	Diabetes Mellitus, Transient Neonatal 3	KCNJ11
202010	Congenital Adrenal Hyperplasia due to 11-beta-Hydroxylase-Deficiency	CYP11B1
201910	Congenital Adrenal Hyperplasia due to 21-Hydroxylase-Deficiency	CYP21A2
202110	Congenital Adrenal Hyperplasia due to 17-alpha Hydroxylase Deficiency	CYP17A1
201400	Adrenocorticotropic hormone Deficiency	TBX19
601198	Hypocalcemia 1	CASR
615361	Hypocalcemia 2	GNA11
146200	Familial Isolated Hypoparathyroidism	PTH,GCM2
239200	Neonatal Severe Hyperparathyroidism	CASR
103580	Pseudohypoparathyroidism Type IA	GNAS
603233	Pseudohypoparathyroidism Type IB	STX16
612462	Pseudohypoparathyroidism Type IC	GNAS
146255	Hypoparathyroidism, Sensorineural Deafness, and Renal Disease	GATA3
241410	Hypoparathyroidism-Retardation-Dysmorphism Syndrome	TBCE
300888	Central Hypothyroidism and Testicular Enlargement	IGSF1
201710	Lipoid Congenital Adrenal Hyperplasia	STAR
274400	Thyroid dyshormonogenesis 1	SLC5A5
274500	Thyroid dyshormonogenesis 2A	TPO
274700	Thyroid dyshormonogenesis 3	TG
274800	Thyroid dyshormonogenesis 4	IYD
274900	Thyroid dyshormonogenesis 5	DUOXA2
607200	Thyroid dyshormonogenesis 6	DUOX2
275200	Hypothyroidism Congenital Nongoitrous 1	TSHR
218700	Hypothyroidism Congenital Nongoitrous 2	PAX8
275100	Hypothyroidism Congenital Nongoitrous 4	TSHB
225250	Hypothyroidism Congenital Nongoitrous 5	NKX2-5
614450	Hypothyroidism Congenital Nongoitrous 6	THRA
203400	Congenital Hypoaldosteronism due to CMO I deficiency	CYP11B2
610600	Congenital Hypoaldosteronism due to CMO II deficiency	CYP11B2
613677	Familial Hyperaldosteronism Type III	KCNJ5
617027	Familial Hyperaldosteronism Type IV	CACNA1H
246200	Donohue Syndrome	INSR
615999	Familial Dysalbuminemic Hyperthyroxinemia	ALB
615363	Estrogen Resistance	ESR1
202200	Glucocorticoid Deficiency 1	MC2R
607398	Glucocorticoid Deficiency 2	MRAP
614736	Glucocorticoid Deficiency 4	NNT
103900	Glucocorticoid-Remediable Aldosteronism	CYP11B1
609152	Nonautoimmune Hyperthyroidism	TSHR
608747	Insulin-Like Growth Factor I Deficiency	IGF1
270450	Insulin-Like Growth Factor I, Resistance to	IGF1R
261000	Intrinsic Factor Deficiency	GIF
262650	Kowarski Syndrome	GH1
125850	Maturity-onset diabetes of the young, type 1	HNF4A
125851	Maturity-onset diabetes of the young, type 2	GCK
600496	Maturity-onset diabetes of the young, type 3	HNF1A
137920	Renal Cysts and Diabetes Syndrome	HNF1B
606394	Maturity-onset diabetes of the young, type 6	NEUROD1
610508	Maturity-onset diabetes of the young, type 7	KLF11
609812	Maturity-onset diabetes of the young, type 8	CEL
612225	Maturity-onset diabetes of the young, type 9	PAX4
613370	Maturity-onset diabetes of the young, type 10	INS
613375	Maturity-onset diabetes of the young, type 11	BLK
616329	Maturity-onset diabetes of the young, type 13	KCNJ11
610489	Pigmented Nodular Adrenocortical Disease, Primary, 1	PRKAR1A
610475	Pigmented Nodular Adrenocortical Disease, Primary, 2	PDE11A
614190	Pigmented Nodular Adrenocortical Disease, Primary, 3	PDE8B
262500	Pituitary Dwarfism II	GHR
600955	Proprotein Convertase-1 Deficiency	PCSK1
145650	Selective Pituitary Thyroid Hormone Resistance	THRB
613743	Adrenal Insufficiency, Congenital, with 46XY Sex Reversal, Partial or Complete	CYP11A1
180920	Aplasia of Lacrimal and Salivary Glands	FGF10
605373	Familial Paragangliomas 3	SDHC
115310	Familial Paragangliomas 4	SDHB
615474	Primary Aldosteronism, Seizures, And Neurologic Abnormalities	CACNA1D
615954	Acth-Independent Macronodular Adrenal Hyperplasia 2	ARMC5
312300	Partial Androgen Insensitivity	AR
139300	Aromatase Excess Syndrome	CYP19A1
611489	Corticosteroid-Binding Globulin Deficiency	SERPINA6
260660	Cousin Syndrome	TBX15
615962	Generalized Glucocorticoid Resistance	NR3C1
138800	Goiter, Multinodular 1, With Or Without Sertoli-Leydig Cell Tumors	DICER1
604271	Partial Growth Hormone Insensitivity	GHR
609968	Familial Hyperinsulinemic Hypoglycemia 5	INSR
603373	Familial Gestational Hyperthyroidism	TSHR
145680	Hyperthyroxinemia, Dystransthyretinemic	TTR
240800	Leucine-Induced Hypoglycemia	ABCC8
612463	Pseudopseudohypoparathyroidism	GNAS
609698	Abnormal Thyroid Hormone Metabolism	SECISBP2
188570	Generalized Thyroid Hormone Resistance, Autosomal Dominant	THRB
274300	Generalized Thyroid Hormone Resistance, Autosomal Recessive	THRB
NA016	UCP2-Related Hyperinsulinism	UCP2
304150	Occipital Horn Syndrome	ATP7A
123700	Autosomal Dominant Cutis Laxa 1	ELN
614434	Autosomal Dominant Cutis Laxa 2	FBLN5
616603	Autosomal Dominant Cutis Laxa 3	ALDH18A1
219100	Autosomal Recessive Cutis Laxa type 1A	FBLN5
614437	Autosomal Recessive Cutis Laxa type 1B	EFEMP2
613177	Autosomal Recessive Cutis Laxa type 1C	LTBP4
219200	Autosomal Recessive Cutis Laxa type 2A	ATP6V0A2
612940	Autosomal Recessive Cutis Laxa type 2B	PYCR1
219150	Autosomal Recessive Cutis Laxa type 3A	ALDH18A1
614438	Autosomal Recessive Cutis Laxa type 3B	PYCR1
242300	Lamellar Ichthyosis	TGM1
242100	Autosomal Recessive Congenital Ichthyosis 2	ALOX12B
606545	Nonbullous Congenital Ichthyosiform Erythroderma	ALOXE3
601277	Ichthyosis, Congenital, Autosomal Recessive 4a	ABCA12
242500	Harlequin ichthyosis	ABCA12
604777	Autosomal Recessive Congenital Ichthyosis 5	CYP4F22
612281	Autosomal Recessive Congenital Ichthyosis 6	NIPAL4
613943	Autosomal Recessive Congenital Ichthyosis 8	LIPN
615023	Autosomal Recessive Congenital Ichthyosis 9	CERS3
615024	Autosomal Recessive Congenital Ichthyosis 10	PNPLA1
602400	Autosomal Recessive Congenital Ichthyosis 11	ST14
308205	Ichthyosis Follicularis-Atrichia-Photophobia Syndrome	MBTPS2
146590	Ichthyosis Hystrix, Curth-Macklin type	KRT1
607602	Cyclic Ichthyosis With Epidermolytic Hyperkeratosis	KRT1, KRT10
602540	Hystrix-Like Ichthyosis With Deafness	GJB2
308100	X-Linked Ichthyosis	STS
608649	Ichthyosis Prematurity Syndrome	SLC27A4
607626	Neonatal Ichthyosis-sclerosing cholangitis Syndrome	CLDN1
607936	Ichthyosis Bullosa Of siemens-Like Exfoliative Ichthyosis	CSTA
146700	Ichthyosis Vulgaris	FLG
256500	Netherton syndrome	SPINK5
609528	Cerebral Dysgenesis, Neuropathy, Ichthyosis, And Palmoplantar Keratoderma Syndrome	SNAP29
614457	Ichthyosis, Spastic Qudraplegia and Mental Retardation	ELOVL4
604117	Vohwinkel syndrome with ichthyosis	LOR
124500	Vohwinkel syndrome	GJB2
148210	Keratitis-Ichthyosis-Deafness Syndrome	GJB2
146800	Ichthyosis Bullosa of Siemens	KRT2
601952	Keratosis Linearis with Ichthyosis Congenita and Sclerosing Keratoderma	POMP
203100	Oculocutaneous Albinism type 1A	TYR
606952	Oculocutaneous Albinism type 1B	TYR
203200	Oculocutaneous Albinism type 2	OCA2
203290	Oculocutaneous Albinism type 3	TYRP1
606574	Oculocutaneous Albinism type 4	SLC45A2
113750	Oculocutaneous Albinism type 6	SLC24A5
615179	Oculocutaneous Albinism type 7	C10orf11
278700	Xeroderma Pigmentosum Group A	XPA
610651	Xeroderma Pigmentosum Group B	ERCC3
278720	Xeroderma Pigmentosum Group C	XPC
278730	Xeroderma Pigmentosum Group D	ERCC2
278740	Xeroderma Pigmentosum Group E	DDB2
278760	Xeroderma Pigmentosum Group F	ERCC4
278780	Xeroderma Pigmentosum Group G	ERCC5
278750	Xeroderma Pigmentosum, Variant type	POLH
305000	X-Linked Dyskeratosis Congenita	DKC1
613989	Dyskeratosis Congenita, Autosomal Dominant, 2	TERT
613990	Dyskeratosis Congenita, Autosomal Dominant, 3	TINF2
616553	Dyskeratosis Congenita, Autosomal Dominant, 6	ACD
224230	Dyskeratosis Congenita, Autosomal Recessive, 1	NOP10
613987	Dyskeratosis Congenita, Autosomal Recessive, 2	NHP2
613988	Dyskeratosis Congenita, Autosomal Recessive, 3	WRAP53
615190	Dyskeratosis Congenita, Autosomal Recessive, 5	RTEL1
616353	Dyskeratosis Congenita, Autosomal Recessive, 6	PARN
130060	Arthrochalasia type Ehlers-Danlos Syndrome	COL1A1, COL1A2
130000	Classic type Ehlers-Danlos Syndrome	COL5A1, COL5A2, COL1A1
130020	Ehlers-Danlos Syndrome type III	COL3A1
130050	Ehlers-Danlos Syndrome type IV	COL3A1
225400	Ehlers-Danlos Syndrome, type VI;	PLOD1
225410	Ehlers-Danlos syndrome type VIIC	ADAMTS2
612350	Ehlers-Danlos Syndrome-Like Spondylocheiro dysplasia	SLC39A13
614557	Ehlers-Danlos Syndrome with Progressive Kyphoscoliosis, Myopathy, and Hearing Loss	FKBP14
130070	Ehlers-Danlos Syndrome, Progeroid type, 1	B4GALT7
225320	Ehlers-Danlos Syndrome, Cardiac Valvular Form	COL1A2
601776	Ehlers-Danlos Syndrome, Musculocontractural type 1	CHST14
615539	Ehlers-Danlos Syndrome, Musculocontractural type 2	DSE
617174	Ehlers-Danlos syndrome, periodontal type, 2	C1S
131760	Dowling-Meara type of Epidermolysis Bullosa Simplex	KRT5, KRT14
226730	Epidermolysis Bullosa with Pyloric Atresia	ITGB4, ITGA6
612138	PLEC-Related Epidermolysis Bullosa with Pyloric Atresia	PLEC
226700	Herlitz type of junctional epidermolysis bullosa	LAMB3, LAMA3, LAMC2
226650	non-Herlitz type Junctional Epidermolysis Bullosa	COL17A1, LAMC2, LAMB3, ITGB4, LAMA3
122400	Epithelial Recurrent Erosion Dystrophy	COL17A1
226600	Autosomal Recessive Epidermolysis Bullosa Dystrophica	COL7A1
131750	Autosomal Dominant Epidermolysis Bullosa Dystrophica	COL7A1
131800	Localized Epidermolysis Bullosa Simplex	ITGB4, KRT14, KRT5
616487	Epidermolysis bullosa simplex with nail dystrophy	PLEC
131850	Pretibial Epidermolysis Bullosa Dystrophica	COL7A1
131900	Generalized Epidermolysis Bullosa Simplex	KRT14,KRT5
131950	Epidermolysis Bullosa Simplex, Ogna type	PLEC
131960	Epidermolysis Bullosa Simplex With Mottled Pigmentation	KRT5
604129	Epidermolysis Bullosa Pruriginosa	COL7A1
609352	Epidermolysis Bullosa Simplex With Migratory Circinate Erythema	KRT5
601001	Autosomal Recessive Epidermolysis Bullosa Simplex 1	KRT5, KRT14
615425	Autosomal Recessive Epidermolysis Bullosa Simplex 2	DST
615028	Nonspecific Autosomal Recessive Epidermolysis Bullosa,	EXPH5
132000	Epidermolysis Bullosa With Congenital Localized Absence Of Skin And deformity Of Nails	COL7A1
151100	Multiple Lentigines Syndrome 1	PTPN11
611554	Multiple Lentigines Syndrome 2	RAF1
613707	Multiple Lentigines Syndrome 3	BRAF
274150	Congenital Thrombotic thrombocytopenic purpura	ADAMTS13
124200	Darier-White Disease	ATP2A2
268400	Rothmund-Thomson Syndrome	RECQL4
305600	Focal dermal hypoplasia	PORCN
228600	Hyaline fibromatosis syndrome	ANTXR2
611431	Legius Syndrome	SPRED1
113800	Epidermolytic hyperkeratosis	KRT1, KRT10
166700	Buschke-Ollendorff Syndrome	LEMD3
123790	Beare-Stevenson Cutis Gyrata syndrome	FGFR2
169600	Benign Chronic Pemphigus	ATP2C1
605041	Brooke-Spiegler syndrome	CYLD
179850	Dowling-Degos disease 1	KRT5
615327	Dowling-Degos disease 2	POFUT1
615696	Dowling-Degos disease 4	POGLUT1
132700	Familial Cylindromatosis	CYLD
173200	Familial Pityriasis Rubra Pilaris	CARD14
607115	CINCA syndrome	NLRP3
172800	Piebaldism	KIT, SNAI2
184500	Steatocystoma Multiplex	KRT17
600630	UV-sensitive syndrome 1	ERCC6
614621	UV-Sensitive Syndrome 2	ERCC8
614640	UV-sensitive syndrome 3	UVSSA
167200	Pachyonychia Congenita 1	KRT16
167210	Pachyonychia Congenita 2	KRT17
615726	Pachyonychia Congenita 3	KRT6A
615728	Pachyonychia Congenita 4	KRT6B
613000	Non Epidermolytic Focal Palmoplantar Keratoderma 1	KRT16
616400	Non Epidermolytic Focal Palmoplantar Keratoderma 2	TRPV3
615735	Non Epidermolytic Focal or Diffuse Palmoplantar Keratoderma	KRT6C
616099	Palmoplantar Keratoderma And Woolly Hair	KANK2
600231	Palmoplantar Keratoderma, Bothnian Type	AQP5
615598	Palmoplantar Keratoderma, Nagashima Type	SERPINB7
148600	Palmoplantar Keratoderma, Punctate Type Ia	AAGAB
104100	Palmoplantar keratoderma and congenital alopecia-1	GJA1
201100	Acrodermatitis Enteropathica, Zinc-Deficiency Type	SLC39A4
610448	Chilblain Lupus 1	TREX1
614415	Chilblain Lupus 2	SAMHD1
615522	Cole Disease	ENPP1
607655	Skin fragility-woolly hair syndrome	DSP
127400	Dyschromatosis Symmetrica Hereditaria	ADAR
615402	Dyschromatosis Universalis Hereditaria 3	ABCB6
226400	Epidermodysplasia Verruciformis	TMC6, TMC8
609638	Epidermolysis Bullosa, Lethal Acantholytic	DSP
144200	Epidermolytic Palmoplantar Keratoderma	KRT9
614204	Generalized Pustular Psoriasis	IL36RN
231070	Geroderma Osteodysplasticum	GORAB
133200	Erythrokeratodermia variabilis et progressiva 1	GJB3, GJB4, GJA1
617525	Erythrokeratodermia variabilis et progressiva 3	GJA1
138000	Glomuvenous Malformation	GLMN
245010	Haim-Munk Syndrome	CTSC
605389	Hypotrichosis 1	APCDD1
146520	Hypotrichosis 2	CDSN
613981	Hypotrichosis 3	KRT74
146550	Hypotrichosis 4	HR
607903	Hypotrichosis 6	DSG4
604379	Hypotrichosis 7	LIPH
278150	Hypotrichosis 8	LPAR6
615059	Hypotrichosis 11	SNRPE
615885	Hypotrichosis 12	RPL21
615896	Hypotrichosis 13	KRT71
615508	Congenital Erythroderma with Palmoplantar Keratoderma, Hypotrichosis and hyper IgE	DSG1
148700	Keratosis Palmoplantaris Striata I	DSG1
612908	Keratosis Palmoplantaris Striata II	DSP
607654	Keratosis Palmoplantaris Striata III	KRT1
173650	Kindler Syndrome	FERMT1
245660	Laryngoonychocutaneous Syndrome	LAMA3
275210	Lethal Restrictive Dermopathy	ZMPSTE24, LMNA
248300	Mal de Meleda	SLURP1
228550	Myofibromatosis, Infantile 1	PDGFRB
600962	Nonepidermolytic Palmoplantar Hyperkeratosis	KRT1
614594	Palmoplantar Keratoderma, Mutilating, with Periorificial Keratotic Plaques	TRPV3
270300	Peeling Skin Syndrome 1	CDSN
609796	Peeling Skin Syndrome 2	TGM5
616265	Peeling Skin Syndrome 3	CHST8
604173	Poikiloderma with Neutropenia	USB1
604416	Pyogenic Sterile Arthritis, Pyoderma Gangrenosum, and Acne	PSTPIP1
268130	Revesz Syndrome	TINF2
184900	Stiff Skin Syndrome	FBN1
308800	X-linked Keratosis Follicularis Spinulosa Decalvans	MBTPS2
300918	X-linked Olmsted Syndrome	MBTPS2
203655	Alopecia Universalis	HR
206800	Anonychia Congenita	RSPO4
209500	Atrichia with Papular Lesions	HR
126700	Basal Laminar Drusen	CFH
142690	Acne Inversa, Familial, 1	NCSTN
613736	Acne Inversa, Familial, 2	PSENEN
613737	Acne Inversa, Familial, 3	PSEN1
101900	Acrokeratosis Verruciformis	ATP2A2
136000	Adermatoglyphia	SMARCAD1
105250	Primary Localized Cutaneous Amyloidosis,1	OSMR
613955	Primary Localized Cutaneous Amyloidosis,2	IL31RA
107600	Nonsyndromic Aplasia Cutis Congenita	BMS1
125595	Dermatopathia Pigmentosa Reticularis	KRT14
609165	Congenital Reticular Ichthyosiform Erythroderma	KRT10
145250	Familial Progressive Hyperpigmentation With Or Without Hypopigmentation	KITLG
613102	Hypotrichosis And Recurrent Skin Vesicles	DSC3
148350	Palmoplantar Keratoderma With Deafness	GJB2
158000	Monilethrix	KRT83
151600	Congenital Nonsyndromic Nail Disorder 3	PLCD1
607523	Congenital Nonsyndromic Nail Disorder 8	COL7A1
614157	Congenital Nonsyndromic Nail Disorder 10	FZD6
610644	Palmoplantar Hyperkeratosis With Squamous Cell Carcinoma Of Skin And sex reversal	RSPO1
175800	Porokeratosis 1, Multiple Types	PMVK
175900	Porokeratosis 3, Disseminated Superficial Actinic Type	MVK
614714	Porokeratosis 7, Multiple Types	MVD
616063	Porokeratosis 8, Disseminated Superficial Actinic Type	SLC17A9
616631	Porokeratosis 9, Multiple Types	FDPS
612318	Pseudofolliculitis Barbae	KRT75
602723	Psoriasis 2	CARD14
615537	Reticulate Acropigmentation Of Kitamura	ADAM10
610227	Seborrhea-Like Dermatitis With Psoriasiform Elements	ZNF750
615934	Infantile-Onset Sting-Associated Vasculopathy	TMEM173
131705	Transient Bullous Dermolysis Of The Newborn	COL7A1
194300	Autosomal Dominant Woolly Hair	KRT74
616760	Autosomal Recessive Woolly Hair 3	KRT25
278250	Wrinkly Skin Syndrome	ATP6V0A2
106190	Isolated Anhidrosis with normal sweat glands	ITPR2
300887	Linear skin defects with multiple congenital anomalies 2	COX7B
300952	Linear skin defects with multiple congenital anomalies 3	NDUFB11
616295	Peeling skin with leukonychia, acral punctate keratoses, cheilitis, and knuckle pads	CAST
125630	Vibratory Urticaria	ADGRE2
156610	Congenital symmetric circumferential skin creases 1	TUBB
616734	Congenital symmetric circumferential skin creases 2	MAPRE2
NA002	Xeroderma Pigmentosum Group H	ERCC1
125800	Autosomal Nephrogenic Diabetes Insipidus	AQP2
304800	X-Linked Nephrogenic Diabetes Insipidus	AVPR2
162200	Neurofibromatosis 1	NF1
101000	Neurofibromatosis 2	NF2
162210	Familial Spinal Neurofibromatosis	NF1
105400	Amyotrophic Lateral Sclerosis 1	SOD1
205100	Amyotrophic Lateral Sclerosis 2	ALS2
602433	Amyotrophic Lateral Sclerosis 4	SETX
602099	Amyotrophic Lateral Sclerosis 5	SPG11
608030	Amyotrophic Lateral Sclerosis 6	FUS
608627	Amyotrophic Lateral Sclerosis 8	VAPB
611895	Amyotrophic Lateral Sclerosis 9	ANG
612069	Amyotrophic Lateral Sclerosis 10 with or without Frontotemporal Dementia	TARDBP
612577	Amyotrophic Lateral Sclerosis 11	FIG4
613435	Amyotrophic Lateral Sclerosis 12	OPTN
613954	Amyotrophic lateral sclerosis 14 with or without frontotemporal dementia	VCP
300857	Amyotrophic Lateral Sclerosis 15 with or without frontotemporal dementia	UBQLN2
614373	Amyotrophic Lateral Sclerosis 16	SIGMAR1
614696	Amyotrophic Lateral Sclerosis 17	CHMP2B
614808	Amyotrophic Lateral Sclerosis 18	PFN1
615515	Amyotrophic Lateral Sclerosis 19	ERBB4
615426	Amyotrophic Lateral Sclerosis 20	HNRNPA1
606070	Amyotrophic Lateral Sclerosis 21	MATR3
616208	Amyotrophic Lateral Sclerosis 22 with or without frontotemporal dementia	TUBA4A
105500	Amyotrophic Lateral Sclerosis-Parkinsonism/Dementia Complex 1	TRPM7
616437	Frontotemporal dementia and/or amyotrophic lateral sclerosis 3	SQSTM1
616439	Frontotemporal dementia and/or amyotrophic lateral sclerosis 4	TBK1
135700	Congenital Fibrosis of the Extraocular Muscles 1	KIF21A
602078	Congenital Fibrosis of the Extraocular Muscles 2	PHOX2A
600638	Congenital Fibrosis of the Extraocular Muscles 3A	TUBB3
616219	Congenital Fibrosis of the Extraocular Muscles 5	COL25A1
213300	Joubert Syndrome 1	INPP5E
608091	Joubert Syndrome 2	TMEM216
608629	Joubert Syndrome 3	AHI1
609583	Joubert Syndrome 4	NPHP1
610188	Joubert Syndrome 5	CEP290
610688	Joubert Syndrome 6	TMEM67
611560	Joubert Syndrome 7	RPGRIP1L
612291	Joubert Syndrome 8	ARL13B
612285	Joubert Syndrome 9	CC2D2A
300804	Joubert Syndrome 10	OFD1
200990	Joubert Syndrome 12	KIF7
614173	Joubert Syndrome 13	TCTN1
614424	Joubert Syndrome 14	TMEM237
614464	Joubert Syndrome 15	CEP41
614465	Joubert Syndrome 16	TMEM138
614615	Joubert Syndrome 17	C5orf42
614815	Joubert Syndrome 18	TCTN3
614970	Joubert Syndrome 20	TMEM231
615636	Joubert Syndrome 21	CSPP1
615665	Joubert Syndrome 22	PDE6D
616490	Joubert Syndrome 23	KIAA0586
616654	Joubert Syndrome 24	TCTN2
616781	Joubert Syndrome 25	CEP104
616784	Joubert Syndrome 26	KIAA0556
617120	Joubert Syndrome 27	B9D1
617121	Joubert Syndrome 28	MKS1
118220	Charcot-Marie-Tooth disease type 1A	PMP22
118200	Charcot-Marie-Tooth disease type 1B	MPZ
601098	Charcot-Marie-Tooth disease type 1C	LITAF
607678	Charcot-Marie-Tooth disease type 1D	EGR2
118300	Charcot-Marie-Tooth disease type 1E	PMP22
607734	Charcot-Marie-Tooth disease type 1F	NEFL
118210	Charcot-Marie-Tooth disease type 2A1	KIF1B
609260	Charcot-Marie-Tooth disease type 2A2	MFN2
600882	Charcot-Marie-Tooth disease type 2B	RAB7A
605588	Charcot-Marie-Tooth disease type 2B1	LMNA
605589	Charcot-Marie-Tooth disease type 2B2	MED25
606071	Charcot-Marie-Tooth disease type 2C	TRPV4
601472	Charcot-Marie-Tooth disease type 2D	GARS
607684	Charcot-Marie-Tooth disease type 2E	NEFL
606595	Charcot-Marie-Tooth disease type 2F	HSPB1
607831	Charcot-Marie-Tooth disease type 2K	GDAP1
607677	Charcot-Marie-Tooth disease type 2I	MPZ
607736	Charcot-Marie-Tooth disease type 2J	MPZ
608673	Charcot-Marie-Tooth disease type 2L	HSPB8
613287	Charcot-Marie-Tooth disease type 2N	AARS
614228	Charcot-Marie-Tooth disease type 2O	DYNC1H1
614436	Charcot-Marie-Tooth disease type 2P	LRSAM1
615025	Charcot-Marie-Tooth disease type 2Q	DHTKD1
615490	Charcot-Marie-Tooth disease type 2R	TRIM2
616155	Charcot-Marie-Tooth Disease, Axonal, type 2s	IGHMBP2
616233	Charcot-Marie-Tooth Disease, Axonal, type 2t	DNAJB2
616280	Charcot-Marie-Tooth Disease, Axonal, type 2u	MARS
616491	Charcot-Marie-Tooth disease, Axonal, type 2v	NAGLU
616625	Charcot-Marie-Tooth Disease, Axonal, Type 2W	HARS
616668	Charcot-Marie-Tooth Disease, Axonal, Type 2X	SPG11
616687	Charcot-Marie-Tooth Disease, Axonal, Type 2Y	VCP
616688	Charcot-Marie-Tooth Disease, Axonal, Type 2Z	MORC2
617087	Charcot-Marie-Tooth disease, axonal, type 2A2B	MFN2
607706	Charcot-Marie-Tooth Disease, Axonal, With Vocal Cord Paresis	GDAP1
145900	Charcot-Marie-Tooth disease type 3	MPZ, PMP22, EGR2, PRX
214400	Charcot-Marie-Tooth disease type 4A	GDAP1
601382	Charcot-Marie-Tooth disease type 4B1	MTMR2
604563	Charcot-Marie-Tooth disease type 4B2	SBF2
615284	Charcot-Marie-Tooth disease type 4B3	SBF1
601596	Charcot-Marie-Tooth disease type 4C	SH3TC2
601455	Charcot-Marie-Tooth disease type 4D	NDRG1
605253	Congenital Hypomyelinating Neuropathy	EGR2, MPZ
614895	Charcot-Marie-Tooth disease type 4F	PRX
609311	Charcot-Marie-Tooth disease type 4H	FGD4
611228	Charcot-Marie-Tooth disease type 4J	FIG4
616684	Charcot-Marie-Tooth disease type 4K	SURF1
606482	Charcot-Marie-Tooth disease, dominant intermediate B	DNM2
608323	Charcot-Marie-Tooth disease, dominant intermediate C	YARS
607791	Charcot-Marie-Tooth disease, dominant intermediate D	MPZ
614455	Charcot-Marie-Tooth disease, dominant intermediate E	INF2
615185	Charcot-Marie-Tooth disease, dominant intermediate F	GNB4
608340	Charcot-Marie-Tooth disease, recessive intermediate A	GDAP1
613641	Charcot-Marie-Tooth disease, recessive intermediate B	KARS
615376	Charcot-Marie-Tooth disease, recessive intermediate, C	PLEKHG5
616039	Charcot-Marie-Tooth disease, recessive intermediate, D	COX6A1
302800	X-linked Charcot-Marie-Tooth disease 1	GJB1
310490	X-linked Charcot-Marie-Tooth disease 4	AIFM1
311070	X-linked Charcot-Marie-Tooth disease 5	PRPS1
300905	X-linked Charcot-Marie-Tooth disease 6	PDK3
611067	Autosomal Recessive Distal Spinal Muscular Atrophy 4	PLEKHG5
607641	Distal Hereditary Motor Neuronopathy type VIIB	DCTN1
243000	Congenital Insensitivity to Pain	SCN9A
614881	Autosomal Recessive Distal Spinal Muscular Atrophy 5	DNAJB2
604484	Hereditary motor and sensory neuropathy, proximal type	TFG
605285	Hereditary Motor and Sensory Neuropathy, Russe type	HK1
218000	Hereditary Motor and Sensory Neuropathy with Agenesis of the Corpus Callosum	SLC12A6
601152	Hereditary Motor And Sensory Neuropathy type VI	MFN2
616505	Hereditary Motor And Sensory Neuropathy type VIB	SLC25A46
615632	Hereditary Sensory Neuropathy type IF	ATL3
256840	Hereditary sensory and autonomic neuropathy with spastic paraplegia	CCT5
137200	Neuromyotonia and Axonal Neuropathy	HINT1
608236	Slowed Nerve Conduction Velocity	ARHGEF10
615290	Childhood-onset proximal spinal muscular atrophy with contractures	BICD2
256850	Giant Axonal Neuropathy	GAN
162500	Hereditary Neuropathy With Liability To Pressure Palsies	PMP22
614116	Hereditary Sensory Neuropathy type IE	DNMT1
182980	Adult-onset proximal spinal muscular atrophy	VAPB
609136	Peripheral Demyelinating Neuropathy, Central Dysmyelination, Waardenburg Syndrome, and Hirschsprung Disease	SOX10
300489	X-linked Distal Spinal Muscular Atrophy 3	ATP7A
162400	Hereditary Sensory and Autonomic Neuropathy type IA	SPTLC1
613640	Hereditary Sensory and Autonomic Neuropathy type IC	SPTLC2
613708	Hereditary Sensory and Autonomic Neuropathy type ID	ATL1
201300	Hereditary Sensory and Autonomic Neuropathy type IIA	WNK1
613115	Hereditary Sensory and Autonomic Neuropathy type IIB	FAM134B
614213	Hereditary Sensory and Autonomic Neuropathy type IIC	KIF1A
223900	Familial Dysautonomia	IKBKAP
256800	Hereditary Sensory and Autonomic Neuropathy IV	NTRK1
608654	Hereditary Sensory and Autonomic Neuropathy type V	NGF
614653	Hereditary Sensory and Autonomic Neuropathy type VI	DST
615548	Hereditary Sensory and Autonomic Neuropathy type VII	SCN11A
609284	Nemaline Myopathy 1	TPM3
161800	Nemaline Myopathy 3	ACTA1
609285	Nemaline Myopathy 4	TPM2
605355	Nemaline Myopathy 5	TNNT1
609273	Nemaline Myopathy 6	KBTBD13
610687	Nemaline Myopathy 7	CFL2
615348	Nemaline Myopathy 8	KLHL40
615731	Nemaline Myopathy 9	KLHL41
616165	Nemaline Myopathy 10	LMOD3
617336	Nemaline myopathy 11	MYPN
611705	Salih Myopathy	TTN
251950	Mitochondrial myopathy with lactic acidosis	PNPLA8
255160	Autosomal Recessive Myosin Storage Myopathy	MYH7
613869	Fatal Infantile Hypertonic Myofibrillar Myopathy	CRYAB
255995	Native American Myopathy	STAC3
255125	Myopathy with Deficiency of ISCU	ISCU
310400	X-Linked Centronuclear Myopathy	MTM1
160150	Centronuclear Myopathy 1	DNM2
255200	Centronuclear Myopathy 2	BIN1
614408	Centronuclear Myopathy 3	MYF6
614807	Centronuclear Myopathy 4	CCDC78
615959	Centronuclear Myopathy 5	SPEG
605820	Inclusion Body Myopathy 2	GNE
605637	Inclusion Body Myopathy 3	MYH2
128100	Dystonia 1	TOR1A
224500	Dystonia 2	HPCA
314250	X-Linked Dystonia-Parkinsonism Syndrome	TAF1
128101	Dystonia 4	TUBB4A
128230	GTP Cyclohydrolase 1-Deficient Dopa-Responsive Dystonia	GCH1
602629	Dystonia 6	THAP1
601042	Dystonia 9	SLC2A1
128200	Familial Paroxysmal Kinesigenic Dyskinesia	PRRT2
159900	Myoclonic Dystonia	SGCE
128235	Rapid-Onset Dystonia-Parkinsonism	ATP1A3
612067	Dystonia 16	PRKRA
612126	Dystonia 18	SLC2A1
614860	Dystonia 23	CIZ1
615034	Dystonia 24	ANO3
615073	Dystonia 25	GNAL
616398	Dystonia 26, myoclonic	KCTD17
616411	Dystonia 27	COL6A3
607371	Juvenile-Onset Dystonia	ACTB
612716	Dopa-responsive dystonia due to sepiapterin reductase deficiency	SPR
605407	Tyrosine Hydroxylase Deficiency	TH
118800	Familial Paroxysmal Nonkinesigenic Dyskinesia	PNKD
613724	Leukoencephalopathy with Dystonia and Motor Neuropathy	SCP2
304700	Deafness-Dystonia-Optic Neuronopathy Syndrome	TIMM8A
167320	Inclusion Body Myopathy with Paget Disease of Bone and Frontotemporal Dementia	VCP
160800	Autosomal Dominate Myotonia Congenita	CLCN1
255700	Autosomal Recessive Myotonia Congenita	CLCN1
168300	Paramyotonia Congenita	SCN4A
170500	Potassium-Sensitive Normokalemic Periodic Paralysis	SCN4A
170400	Hypokalemic Periodic Paralysis type 1	CACNA1S
613345	Hypokalemic Periodic Paralysis type 2	SCN4A
608390	Potassium-aggravated myotonia	SCN4A
613280	Hypermanganesemia With Dystonia 1	SLC30A10
117000	Central Core Disease	RYR1
255320	Multiminicore disease	RYR1, SEPN1
255310	Congenital fiber-type disproportion myopathy	ACTA1, SEPN1, TPM3
254130	Miyoshi Distal Myopathy	DYSF
613319	Miyoshi Muscular Dystrophy 3	ANO5
608358	Myosin Storage Myopathy	MYH7
614321	Distal Myopathy, Tateyama type	CAV3
606768	Distal Myopathy With Anterior Tibial Onset	DYSF
182920	Myopathy, Spheroid Body	MYOT
160565	Myopathy, tubular aggregate, 1	STIM1
615883	Myopathy, Tubular Aggregate, 2	ORAI1
616231	Myopathy, Vacuolar, With Casq1 Aggregates	CASQ1
254090	Ullrich congenital muscular dystrophy 1	COL6A1, COL6A2, COL6A3
616470	Ullrich congenital muscular dystrophy 2	COL12A1
617066	Muscular dystrophy, congenital, Davignon-Chauveau type	TRIP4
601003	Brody Myopathy	ATP2A1
181430	MYH7-Related Scapuloperoneal Myopathy	MYH7
600334	Tibial Muscular Dystrophy	TTN
164300	Oculopharyngeal Muscular Dystrophy	PABPN1
160500	Laing Distal Myopathy	MYH7
614065	Distal Myopathy 4	FLNC
607855	Merosin-deficient congenital muscular dystrophy type 1A	LAMA2
310200	Duchenne Muscular Dystrophy	DMD
300376	Becker Muscular Dystrophy	DMD
236670	Congenital Muscular Dystrophy-Dystroglycanopathy with Brain and Eye Anomalies type A1	POMT1
613150	Congenital Muscular Dystrophy-Dystroglycanopathy with Brain and Eye Anomalies type A 2	POMT2
253280	Congenital Muscular Dystrophy-Dystroglycanopathy with Brain and Eye Anomalies type A 3	POMGNT1
253800	Congenital Muscular Dystrophy-Dystroglycanopathy with Brain and Eye Anomalies type A 4	FKTN
613153	Congenital Muscular Dystrophy-Dystroglycanopathy with Brain and Eye Anomalies type A 5	FKRP
613154	Congenital Muscular Dystrophy-Dystroglycanopathy with Brain and Eye Anomalies type A 6	LARGE
614643	Congenital Muscular Dystrophy-Dystroglycanopathy with Brain and Eye Anomalies type A 7	ISPD
614830	Congenital Muscular Dystrophy-Dystroglycanopathy with Brain and Eye Anomalies type A 8	POMGNT2
616538	Congenital Muscular Dystrophy-Dystroglycanopathy with Brain and Eye Anomalies type A 9	DAG1
615041	Congenital Muscular Dystrophy-Dystroglycanopathy with Brain and Eye Anomalies type A 10	TMEM5
615249	Congenital Muscular Dystrophy-Dystroglycanopathy with Brain and Eye Anomalies type A 12	POMK
615287	Congenital Muscular Dystrophy-Dystroglycanopathy with Brain and Eye Anomalies type A 13	B3GNT1
615350	Congenital Muscular Dystrophy-Dystroglycanopathy with Brain and Eye Anomalies type A 14	GMPPB
613155	Muscular Dystrophy-Dystroglycanopathy (Congenital With Mental Retardation),type B1	POMT1
613156	Muscular Dystrophy-Dystroglycanopathy (Congenital With Mental Retardation),type B2	POMT2
613151	Muscular Dystrophy-Dystroglycanopathy (Congenital With Mental Retardation),type B3	POMGNT1
613152	Muscular Dystrophy-Dystroglycanopathy (Congenital Without Mental Retardation),type B4	FKTN
606612	Muscular Dystrophy-Dystroglycanopathy (Congenital With Or Without Mental Retardation),type B5	FKRP
608840	Muscular Dystrophy-Dystroglycanopathy (Congenital With Mental Retardation),type B6	LARGE
615351	Muscular Dystrophy-Dystroglycanopathy (Congenital With Mental Retardation),type B14	GMPPB
616052	Muscular Dystrophy-Dystroglycanopathy (Limb-Girdle), type C, 7	ISPD
159000	Limb-Girdle Muscular Dystrophy type 1A	MYOT
159001	Limb-Girdle Muscular Dystrophy type 1B	LMNA
607801	Limb-Girdle Muscular Dystrophy type 1C	CAV3
603511	Limb-Girdle Muscular Dystrophy type 1E	DNAJB6
608423	Limb-Girdle Muscular Dystrophy type 1F	TNPO3
609115	Limb-Girdle Muscular Dystrophy type 1G	HNRNPDL
253600	Limb-Girdle Muscular Dystrophy type 2A	CAPN3
253601	Limb-Girdle Muscular Dystrophy type 2B	DYSF
253700	Limb-Girdle Muscular Dystrophy type 2C	SGCG
608099	Limb-Girdle Muscular Dystrophy type 2D	SGCA
604286	Limb-Girdle Muscular Dystrophy type 2E	SGCB
601287	Limb-Girdle Muscular Dystrophy type 2F	SGCD
601954	Limb-Girdle Muscular Dystrophy type 2G	TCAP
254110	Limb-Girdle Muscular Dystrophy type 2H	TRIM32
607155	Limb-Girdle Muscular Dystrophy type 2I	FKRP
608807	Limb-Girdle Muscular Dystrophy type 2J	TTN
609308	Limb-Girdle Muscular Dystrophy type 2K	POMT1
611307	Limb-Girdle Muscular Dystrophy type 2L	ANO5
611588	Limb-Girdle Muscular Dystrophy type 2M	FKTN
613158	Limb-Girdle Muscular Dystrophy type 2N	POMT2
613157	Limb-Girdle Muscular Dystrophy type 2O	POMGNT1
613818	Limb-Girdle Muscular Dystrophy type 2P	DAG1
613723	Limb-Girdle Muscular Dystrophy type 2Q	PLEC
615325	Limb-Girdle Muscular Dystrophy type 2R	DES
615356	Limb-Girdle Muscular Dystrophy type 2S	TRAPPC11
615352	Limb-Girdle Muscular Dystrophy type 2T	GMPPB
616812	Limb-Girdle Muscular Dystrophy type 2X	BVES
617232	Limb-Girdle Muscular Dystrophy type 2Z	POGLUT1
616094	Muscular dystrophy-dystroglycanopathy (limb-girdle), type C, 12	POMK
226670	Epidermolysis Bullosa Simplex with Muscular Dystrophy	PLEC
300695	X-Linked Scapuloperoneal Myopathy	FHL1
300717	X-linked Reducing Body Myopathy-1 with infantile or early childhood onset	FHL1
300718	X-linked Reducing Body Myopathy-1 with late childhood or adult onset	FHL1
613204	Congenital Muscular Dystrophy due to Integrin Alpha-7 Deficiency	ITGA7
602541	Congenital Muscular Dystrophy, Megaconial type	CHKB
310300	X-linked Emery-Dreifuss Muscular Dystrophy 1	EMD
181350	Emery-Dreifuss muscular dystrophy 2	LMNA
616516	Emery-Dreifuss Muscular Dystrophy 3	LMNA
612998	Emery-Dreifuss muscular dystrophy 4	SYNE1
612999	Emery-Dreifuss muscular dystrophy 5	SYNE2
300696	X-linked Emery-Dreifuss Muscular Dystrophy 6	FHL1
614302	Emery-Dreifuss muscular dystrophy 7	TMEM43
601462	Congenital Myasthenic Syndrome 1A	CHRNA1
608930	Congenital Myasthenic Syndrome 1B	CHRNA1
616313	Congenital Myasthenic Syndrome 2A, slow-channel	CHRNB1
616314	Congenital Myasthenic Syndrome 2C, associated with acetylcholine receptor deficiency	CHRNB1
616321	Congenital Myasthenic Syndrome 3A, slow-channel	CHRND
616322	Congenital Myasthenic Syndrome 3B, fast-channel	CHRND
616323	Congenital Myasthenic Syndrome 3C, associated with acetylcholine receptor deficiency	CHRND
605809	Congenital Myasthenic Syndrome 4A, slow-channel	CHRNE
616324	Congenital Myasthenic Syndrome 4B, fast-channel	CHRNE
608931	Congenital Myasthenic Syndrome 4C, associated with acetylcholine receptor deficiency	CHRNE
603034	Congenital Myasthenic Syndrome 5	COLQ
254210	Congenital Myasthenic Syndrome 6	CHAT
616040	Congenital Myasthenic Syndrome 7	SYT2
615120	Congenital Myasthenic Syndrome 8	AGRN
616325	Congenital Myasthenic Syndrome 9	MUSK
254300	Congenital Myasthenic Syndrome 10	DOK7
616326	Congenital Myasthenic Syndrome 11	RAPSN
610542	Congenital Myasthenic Syndrome 12	GFPT1
614750	Congenital Myasthenic Syndrome 13	DPAGT1
616228	Congenital Myasthenic Syndrome 14	ALG2
616227	Congenital Myasthenic Syndrome 15	ALG14
614198	Congenital Myasthenic Syndrome 16	SCN4A
616304	Congenital Myasthenic Syndrome 17	LRP4
616330	Congenital Myasthenic Syndrome 18	SNAP25
616720	Congenital Myasthenic Syndrome 19	COL13A1
617143	Congenital Myasthenic Syndrome 20	SLC5A7
616224	Congenital Myasthenic Syndrome 22	PREPL
601419	Myofibrillar Myopathy 1	DES
608810	Myofibrillar Myopathy 2	CRYAB
609200	Myofibrillar Myopathy 3	MYOT
609452	Myofibrillar Myopathy 4	LDB3
609524	Myofibrillar Myopathy 5	FLNC
612954	Myofibrillar Myopathy 6	BAG3
303350	X-Linked Spastic Paraplegia 1	L1CAM
312920	X-Linked Spastic paraplegia 2	PLP1
182600	Autosomal Dominant Spastic paraplegia 3A	ATL1
182601	Autosomal Dominant Spastic paraplegia 4	SPAST
600363	Autosomal Dominant Spastic paraplegia 6	NIPA1
603563	Autosomal Dominant Spastic paraplegia 8	KIAA0196
601162	Autosomal Dominant Spastic paraplegia 9A	ALDH18A1
604187	Autosomal Dominant Spastic paraplegia 10	KIF5A
604805	Autosomal Dominant Spastic paraplegia 12	RTN2
605280	Autosomal Dominant Spastic paraplegia 13	HSPD1
270685	Autosomal Dominant Spastic paraplegia 17	BSCL2
610250	Autosomal Dominant Spastic paraplegia 31	REEP1
610244	Autosomal Dominant Spastic paraplegia 33	ZFYVE27
612539	Autosomal Dominant Spastic paraplegia 42	SLC33A1
616282	Autosomal Dominant Spastic paraplegia 73	CPT1C
270800	Autosomal Recessive Spastic paraplegia 5A	CYP7B1
616586	Autosomal Recessive Spastic Paraplegia 9B	ALDH18A1
604360	Hereditay Spastic Paraplegia with a Thin Corpus Callosum	SPG11
270700	Autosomal Recessive Spastic paraplegia 15	ZFYVE26
611225	Autosomal Recessive Spastic paraplegia 18	ERLIN2
275900	Troyer syndrome	SPG20
248900	Autosomal Recessive Spastic paraplegia 21	SPG21
609195	Autosomal Recessive Spastic paraplegia 26	B4GALNT1
609340	Autosomal Recessive Spastic paraplegia 28	DDHD1
610357	Autosomal Recessive Spastic paraplegia 30	KIF1A
612020	Autosomal Recessive Spastic paraplegia 39	PNPLA6
615043	Autosomal Recessive Spastic paraplegia 43	C19orf12
613162	Autosomal Recessive Spastic paraplegia 45	NT5C2
614409	Autosomal Recessive Spastic paraplegia 46	GBA2
614066	Autosomal Recessive Spastic paraplegia 47	AP4B1
613647	Autosomal Recessive Spastic paraplegia 48	AP5Z1
615031	Autosomal Recessive Spastic paraplegia 49	TECPR2
612936	Autosomal Recessive Spastic paraplegia 50	AP4M1
613744	Autosomal Recessive Spastic paraplegia 51	AP4E1
614067	Autosomal Recessive Spastic paraplegia 52	AP4S1
614898	Autosomal Recessive Spastic paraplegia 53	VPS37A
615033	Autosomal Recessive Spastic paraplegia 54	DDHD2
615035	Autosomal Recessive Spastic paraplegia 55	C12orf65
615030	Autosomal Recessive Spastic paraplegia 56	CYP2U1
615658	Autosomal Recessive Spastic paraplegia 57	TFG
615685	Autosomal Recessive Spastic paraplegia 61	ARL6IP1
615681	Autosomal Recessive Spastic paraplegia 62	ERLIN1
615686	Autosomal Recessive Spastic paraplegia 63	AMPD2
615683	Autosomal Recessive Spastic paraplegia 64	ENTPD1
615625	Autosomal Recessive Spastic paraplegia 72	REEP2
616451	Autosomal Recessive Spastic paraplegia 74	IBA57
616680	Autosomal Recessive Spastic paraplegia 75	MAG
616907	Autosomal Recessive Spastic paraplegia 76	CAPN1
617046	Autosomal Recessive Spastic paraplegia 77	FARS2
617225	Autosomal Recessive Spastic paraplegia 78	ATP13A2
616756	Spastic Paraplegia And Psychomotor Retardation With Or Without Seizures	HACE1
609541	Spastic Paraplegia, Optic Atrophy, And Neuropathy	KLC2
221770	Polycystic Lipomembranous Osteodysplasia with Sclerosing Leukoencephalopathy	TREM2, TYROBP
249900	Metachromatic leukodystrophy due to Saposin B deficiency	PSAP
616763	Leukodystrophy And Acquired Microcephaly With Or Without Dystonia	PLEKHG2
192315	Retinal Vasculopathy with Cerebral Leukodystrophy	TREX1
169500	Adult-Onset Leukodystrophy	LMNB1
312080	Pelizaeus-Merzbacher disease	PLP1
260600	Hypomyelinating Leukodystrophy 3	AIMP1
612233	Hypomyelinating Leukodystrophy 4	HSPD1
612438	Hypomyelinating Leukodystrophy 6	TUBB4A
614381	Hypomyelinating Leukodystrophy 8	POLR3B
616140	Hypomyelinating Leukodystrophy 9	RARS
616420	Hypomyelinating Leukodystrophy 10	PYCR2
616494	Hypomyelinating Leukodystrophy 11	POLR1C
616683	Hypomyelinating Leukodystrophy 12	VPS11
616881	Hypomyelinating Leukodystrophy 13	C11orf73
225750	Aicardi-Goutieres Syndrome 1	TREX1
610181	Aicardi-Goutieres Syndrome 2	RNASEH2B
610329	Aicardi-Goutieres Syndrome 3	RNASEH2C
610333	Aicardi-Goutieres Syndrome 4	RNASEH2A
612952	Aicardi-Goutieres Syndrome 5	SAMHD1
615010	Aicardi-Goutieres Syndrome 6	ADAR
615846	Aicardi-Goutieres Syndrome 7	IFIH1
203450	Alexander Disease	GFAP
271900	Canavan Disease	ASPA
603896	Leukoencephalopathy with Vanishing White Matter	EIF2B1, EIF2B2, EIF2B3, EIF2B4, EIF2B5
604004	Megalencephalic Leukoencephalopathy with Subcortical Cysts 1	MLC1
613925	Megalencephalic Leukoencephalopathy with Subcortical Cysts 2A	HEPACAM
613926	Megalencephalic Leukoencephalopathy with Subcortical Cysts 2B	HEPACAM
221820	Hereditary Diffuse Leukoencephalopathy with Spheroids	CSF1R
270200	Sjogren-Larsson syndrome	ALDH3A2
300100	X-Linked Adrenoleukodystrophy	ABCD1
614924	Combined Oxidative Phosphorylation Deficiency 12	EARS2
159950	Spinal Muscular Atrophy With Progressive Myoclonic Epilepsy	ASAH1
604320	Autosomal Recessive Distal Spinal Muscular Atrophy 1	IGHMBP2
301830	X-Linked Infantile Spinal Muscular Atrophy	UBA1
605726	Autosomal Recessive Distal Spinal Muscular Atrophy 2	RAX2
158600	Autosomal Dominant Lower Extremity-Predominant Spinal Muscular Atrophy 1	DYNC1H1
600175	Congenital Distal Spinal Muscular Atrophy	TRPV4
600794	Neuropathy, distal hereditary motor, type VA	GARS, BSCL2
300672	Early Infantile Epileptic Encephalopathy 2	CDKL5
609304	Early Infantile Epileptic Encephalopathy 3	SLC25A22
612164	Early Infantile Epileptic Encephalopathy 4	STXBP1
613477	Early Infantile Epileptic Encephalopathy 5	SPTAN1
607208	Early Infantile Epileptic Encephalopathy 6	SCN1A, SCN9A, GABRG2
613720	Early Infantile Epileptic Encephalopathy 7	KCNQ2
300607	Early Infantile Epileptic Encephalopathy 8	ARHGEF9
300088	Early Infantile Epileptic Encephalopathy 9	PCDH19
613402	Early Infantile Epileptic Encephalopathy 10	PNKP
613721	Early Infantile Epileptic Encephalopathy 11	SCN2A
613722	Early Infantile Epileptic Encephalopathy 12	PLCB1
614558	Early Infantile Epileptic Encephalopathy 13	SCN8A
614959	Early Infantile Epileptic Encephalopathy 14	KCNT1
615006	Early Infantile Epileptic Encephalopathy 15	ST3GAL3
615338	Early Infantile Epileptic Encephalopathy 16	TBC1D24
615473	Early Infantile Epileptic Encephalopathy 17	GNAO1
615476	Early Infantile Epileptic Encephalopathy 18	SZT2
615744	Early Infantile Epileptic Encephalopathy 19	GABRA1
300868	Multiple congenital anomalies-hypotonia-seizures syndrome 2	PIGA
615833	Early Infantile Epileptic Encephalopathy 21	NECAP1
615859	Early Infantile Epileptic Encephalopathy 23	DOCK7
615871	Early Infantile Epileptic Encephalopathy 24	HCN1
615905	Early Infantile Epileptic Encephalopathy 25	SLC13A5
616056	Early Infantile Epileptic Encephalopathy 26	KCNB1
616139	Early Infantile Epileptic Encephalopathy 27	GRIN2B
616211	Early Infantile Epileptic Encephalopathy 28	WWOX
616339	Early Infantile Epileptic Encephalopathy 29	AARS
616341	Early Infantile Epileptic Encephalopathy 30	SIK1
616346	Early Infantile Epileptic Encephalopathy 31	DNM1
616366	Early Infantile Epileptic Encephalopathy 32	KCNA2
616409	Early Infantile Epileptic Encephalopathy 33	EEF1A2
616645	Early Infantile Epileptic Encephalopathy 34	SLC12A5
616647	Early Infantile Epileptic Encephalopathy 35	ITPA
615369	Childhood-onset Epileptic Encephalopathy	CHD2
615553	Arthrogryposis, Mental Retardation and Seizures	SLC35A3
605751	Benign Familial Infantile Seizures 2	PRRT2
607745	Benign Familial Infantile Seizures 3	SCN2A
617080	Benign Familial Infantile Seizures 5	SCN8A
266100	Pyridoxine-Dependent Epilepsy	ALDH7A1
610090	Pyridoxal 5'-Phosphate-dependent Epilepsy	PNPO
606777	Glucose Transporter Type 1 Deficiency Syndrome	SLC2A1
608885	GLUT1 Deficiency Syndrome With Pseudohyperkalemia And Hemolysis	SLC2A1
612437	Progressive Myoclonic Epilepsy 1B	PRICKLE1
254780	Lafora Disease	EPM2A, NHLRC1
611726	Progressive Myoclonic Epilepsy 3	KCTD7
254900	Progressive Myoclonic Epilepsy 4	SCARB2
613832	Progressive Myoclonic Epilepsy 5	PRICKLE2
614018	Progressive Myoclonic Epilepsy 6	GOSR2
616187	Progressive Myoclonic Epilepsy 7	KCNC1
616540	Progressive Myoclonic Epilepsy 9	LMNB2
607596	Pontocerebellar hypoplasia type 1A	VRK1
614678	Pontocerebellar Hypoplasia type 1B	EXOSC3
277470	Pontocerebellar hypoplasia type 2A	TSEN54
612389	Pontocerebellar hypoplasia type 2B	TSEN2
612390	Pontocerebellar hypoplasia type 2C	TSEN34
613811	Pontocerebellar Hypoplasia type 2D	SEPSECS
608027	Pontocerebellar Hypoplasia type 3	PCLO
225753	Pontocerebellar hypoplasia type 4	TSEN54
610204	Pontocerebellar hypoplasia type 5	TSEN54
611523	Pontocerebellar hypoplasia type 6	RARS2
614961	Pontocerebellar hypoplasia type 8	CHMP1A
615809	Pontocerebellar hypoplasia type 9	AMPD2
615803	Pontocerebellar hypoplasia type 10	CLP1
616486	Primary Autosomal Recessive Microcephaly 15	MFSD2A
616681	Primary Autosomal Recessive Microcephaly 16	ANKLE2
600513	Nocturnal Frontal Lobe Epilepsy 1	CHRNA4
605375	Nocturnal Frontal Lobe Epilepsy 3	CHRNB2
610353	Nocturnal Frontal Lobe Epilepsy 4	CHRNA2
615005	Nocturnal Frontal Lobe Epilepsy 5	KCNT1
604403	Epilepsy, generalized, with febrile seizures plus, type 2	SCN1A
613863	Epilepsy, generalized, with febrile seizures plus, type 7	SCN9A
607876	Familial Adult Myoclonic Epilepsy 2	ADRA2B
615400	Familial Adult Myoclonic Epilepsy 5	CNTN2
616172	Epilepsy, generalized, with febrile seizures plus, type 9	STX1B
604352	Familial Febrile Seizures 4	ADGRV1
611277	Familial Febrile Seizures 8	GABRG2
614418	Familial Febrile Seizures 11	CPA6
254770	EFHC1-Related Juvenile Myoclonic Epilepsy	EFHC1
605021	Familial Infantile Myoclonic Epilepsy	TBC1D24
121200	Seizures, benign neonatal, type 1	KCNQ2
121201	Seizures, benign neonatal, type 2	KCNQ3
600512	Epilepsy, Familial Temporal Lobe, 1	LGI1
614417	Epilepsy, Familial Temporal Lobe, 5	CPA6
616436	Epilepsy, Familial Temporal Lobe, 7	RELN
616461	Epilepsy, Familial Temporal Lobe, 8	GAL
610042	Cortical Dysplasia-Focal Epilepsy Syndrome	CNTNAP2
604364	Familial Focal Epilepsy with Variable Foci	DEPDC5
609446	Generalized Epilepsy and Paroxysmal Dyskinesia	KCNMA1
607432	Lissencephaly 1	PAFAH1B1
257320	Lissencephaly 2	RELN
611603	Lissencephaly 3	TUBA1A
614019	Lissencephaly 4	NDE1
615191	Lissencephaly 5	LAMB1
616212	Lissencephaly 6	KATNB1
616342	Lissencephaly 7	CDK5
300067	X-linked Lissencephaly 1	DCX
605899	Glycine encephalopathy	AMT, GLDC, GCSH
608097	Periventricular Heterotopia	ARFGEF2
300049	X-Linked Periventricular Heterotopia	FLNA
615544	Periventricular Nodular Heterotopia 6	ERMARD
617201	Periventricular Nodular Heterotopia 7	NEDD4L
245570	Focal Epilepsy with Speech Disorder with or without Mental Retardation	GRIN2A
611105	Leukoencephalopathy with Brain Stem and Spinal Cord Involvement and Lactate Elevation	DARS2
175780	Porencephaly 1	COL4A1
614483	Porencephaly 2	COL4A2
191100	Tuberous Sclerosis 1	TSC1
613254	Tuberous Sclerosis 2	TSC2
168605	Perry Syndrome	DCTN1
261540	Peters Plus Syndrome	B3GALTL
133020	Primary Erythermalgia	SCN9A
200150	Chorea-Acanthocytosis	VPS13A
610313	Cold-induced Sweating Syndrome 2	CLCF1
617055	Cold-induced Sweating Syndrome 3	KLHL7
602066	Familial Infantile Convulsions with Paroxysmal Choreoathetosis	PRRT2
600795	Chromosome 3-Linked Frontotemporal Dementia	CHMP2B
603218	Huntington Disease-Like 1	PRNP
137440	Gerstmann-Straussler Disease	PRNP
312750	Rett Syndrome	MECP2
300673	MECP2-Related Severe Neonatal Encephalopathy	MECP2
300842	McLeod Neuroacanthocytosis Syndrome	XK
602473	Ethylmalonic Encephalopathy	ETHE1
256710	Elejalde Disease	MYO5A
211530	Brown-Vialetto-Van Laere syndrome 1	SLC52A3
614707	Brown-Vialetto-Van Laere syndrome 2	SLC52A2
604218	Familial Encephalopathy with Neuroserpin Inclusion Bodies	SERPINI1
304100	X-linked partial agenesis of the corpus callosum	L1CAM
616819	Agenesis Of Corpus Callosum with Facial Anomalies And Cerebellar Ataxia	FRMD4A
167400	Paroxysmal Extreme Pain Disorder	SCN9A
160120	Episodic Ataxia Type 1	KCNA1
108500	Episodic Ataxia Type 2	CACNA1A
613855	Episodic Ataxia Type 5	CACNB4
612656	Episodic Ataxia Type 6	SLC1A3
600224	Spinocerebellar ataxia type 5	SPTBN2
604432	Spinocerebellar ataxia type 11	TTBK2
605361	Spinocerebellar ataxia type 14	PRKCG
606658	Spinocerebellar ataxia type 15	ITPR1
607346	Spinocerebellar ataxia type 19	KCND3
607454	Spinocerebellar ataxia type 21	TMEM240
610245	Spinocerebellar ataxia type 23	PDYN
609306	Spinocerebellar ataxia type 26	EEF2
609307	Spinocerebellar ataxia type 27	FGF14
610246	Spinocerebellar ataxia type 28	AFG3L2
117360	Spinocerebellar ataxia type 29	ITPR1
133190	Spinocerebellar ataxia type 34	ELOVL4
613908	Spinocerebellar ataxia type 35	TGM6
615957	Spinocerebellar ataxia type 38	ELOVL5
616053	Spinocerebellar ataxia type 40	CCDC88C
616410	Spinocerebellar ataxia type 41	TRPC3
616795	Spinocerebellar ataxia type 42	CACNA1G
213200	Autosomal Recessive Spinocerebellar Ataxia 2	PMPCA
616354	Autosomal Recessive Spinocerebellar ataxia 20	SNX14
616719	Autosomal Recessive Spinocerebellar ataxia 21	SCYL1
616948	Autosomal Recessive Spinocerebellar ataxia 22	VWA3B
616949	Autosomal Recessive Spinocerebellar ataxia 23	TDP2
604121	Autosomal dominant Cerebellar ataxia, deafness, and narcolepsy	DNMT1
108600	Spastic ataxia 1	VAMP1
604391	Ataxia-Telangiectasia-Like Disorder 1	MRE11A
615919	Ataxia-Telangiectasia-Like Disorder 2	PCNA
277460	Ataxia with vitamin E deficiency	TTPA
208920	Ataxia with oculomotor apraxia type 1	APTX
271245	Infantile-Onset Spinocerebellar Ataxia	C10orf2
611302	Spastic Ataxia 2	KIF1C
611390	Spastic Ataxia 3	MARS2
613672	Spastic Ataxia 4	MTPAP
614487	Spastic Ataxia 5	AFG3L2
270550	Autosomal Recessive Spastic Ataxia of Charlevoix-Saguenay	SACS
607250	Autosomal Recessive Spinocerebellar Ataxia with Axonal Neuropathy	TDP1
609033	Ataxia, posterior column, with retinitis pigmentosa	FLVCR1
615651	Leukoencephalopathy with ataxia	CLCN2
607259	Autosomal Recessive Spastic paraplegia 7	SPG7
615960	Poretti-Boltshauser syndrome	LAMA1
616267	Ataxia-oculomotor apraxia 4	PNKP
616291	Lichtenstein-Knorr syndrome	SLC9A1
614559	Infantile Cerebellar-Retinal Degeneration	ACO2
612780	Seizures, Sensorineural Deafness, Ataxia, Mental Retardation, and Electrolyte Imbalance Syndrome	KCNJ10
601238	Cerebellar Ataxia, Cayman Type	ATCAY
213700	Cerebrotendinous xanthomatosis	CYP27A1
606002	Autosomal Recessive Spinocerebellar Ataxia 1	SETX
606937	Autosomal Recessive Spinocerebellar Ataxia 5	WDR73
609270	Autosomal Recessive Spinocerebellar Ataxia 7	TPP1
610743	Autosomal Recessive Spinocerebellar Ataxia 8	SYNE1
613728	Autosomal Recessive Spinocerebellar Ataxia 10	ANO10
614229	Autosomal Recessive Spinocerebellar Ataxia 11	SYT14
614322	Autosomal Recessive Spinocerebellar Ataxia 12	WWOX
614831	Autosomal Recessive Spinocerebellar Ataxia 13	GRM1
615386	Autosomal Recessive Spinocerebellar Ataxia 14	SPTBN2
615705	Autosomal Recessive Spinocerebellar Ataxia 15	KIAA0226
615768	Autosomal Recessive Spinocerebellar Ataxia 16	STUB1
616127	Autosomal Recessive Spinocerebellar Ataxia 17	CWF19L1
616204	Autosomal Recessive Spinocerebellar Ataxia 18	GRID2
117210	Spinocerebellar Ataxia Type 31	BEAN1
302500	X-Linked Spinocerebellar Ataxia 1	ATP2B3
229300	Friedreich Ataxia	FXN
149400	Hereditary Hyperekplexia 1	GLRA1
614619	Hereditary Hyperekplexia 2	GLRB
614618	Hereditary Hyperekplexia 3	SLC6A5
104290	Alternating Hemiplegia of Childhood 1	ATP1A2
614820	Alternating Hemiplegia of Childhood 2	ATP1A3
258450	Progressive External Ophthalmoplegia With Mitochondrial DNA Deletions, Autosomal Recessive 1	POLG
616479	Progressive External Ophthalmoplegia with Mitochondrial DNA Deletions, Autosomal Recessive 2	RNASEH1
617069	Progressive external ophthalmoplegia with mitochondrial DNA deletions, autosomal recessive 3	TK2
617070	Progressive external ophthalmoplegia with mitochondrial DNA deletions, autosomal recessive 4	DGUOK
609283	Progressive External Ophthalmoplegia with Mitochondrial DNA Deletions, Autosomal Dominant 2	SLC25A4
609286	Progressive External Ophthalmoplegia with Mitochondrial DNA Deletions, Autosomal Dominant 3	C10orf2
610131	Progressive External Ophthalmoplegia with Mitochondrial DNA Deletions, Autosomal Dominant 4	POLG2
613077	Progressive External Ophthalmoplegia with Mitochondrial DNA Deletions, Autosomal Dominant 5	RRM2B
615156	Progressive External Ophthalmoplegia with Mitochondrial DNA Deletions, Autosomal Dominant 6	DNA2
613730	Hemorrhagic Destruction of the Brain, Subependymal Calcification and Cataracts	JAM3
604168	Congenital Cataracts, Facial Dysmorphism, and Neuropathy	CTDP1
600145	Sacral defect with anterior meningocele	VANGL1
603513	Spastic Quadriplegic Cerebral Palsy 1	GAD1
610978	Brain-Lung-Thyroid Syndrome	NKX2-1
601536	Athabaskan Brain Stem Dysgenesis Syndrome	HOXA1
604213	Chudley-McCullough Syndrome	GPSM2
310700	X-Linked Congenital Nystagmus 1	FRMD7
614039	Cortical Dysplasia, Complex, with Other Brain Malformations 1	TUBB3
615282	Cortical Dysplasia, Complex, with Other Brain Malformations 2	KIF5C
615411	Cortical Dysplasia, Complex, with Other Brain Malformations 3	KIF2A
615412	Cortical Dysplasia, Complex, with Other Brain Malformations 4	TUBG1
615771	Cortical Dysplasia, Complex, with Other Brain Malformations 6	TUBB
602398	Desmosterolosis	DHCR24
158590	Distal Hereditary Motor Neuronopathy, Type IIA	HSPB8
608634	Distal Hereditary Motor Neuronopathy, Type IIB	HSPB1
613376	Distal Hereditary Motor Neuronopathy, Type IIC	HSPB3
615575	Distal Hereditary Motor Neuronopathy, Type IID	FBXO38
614751	Distal Hereditary Motor Neuronopathy, Type VB	REEP1
158580	Distal Hereditary Motor Neuropathy, Type VIIA	SLC5A7
613135	Infantile Parkinsonism-Dystonia	SLC6A3
158901	Facioscapulohumeral Muscular Dystrophy 2, digenic	SMCHD1, DUX4
116860	Familial Cerebral Cavernous Malformation 1	KRIT1
603284	Familial Cerebral Cavernous Malformation 2	CCM2
603285	Familial Cerebral Cavernous Malformation 3	PDCD10
614564	Familial Cutaneous Telangiectasia and Cancer Syndrome	ATR
615040	Familial Episodic Pain Syndrome 1	TRPA1
615551	Familial Episodic Pain Syndrome 2	SCN10A
615552	Familial Episodic Pain Syndrome 3	SCN11A
141500	Familial Hemiplegic Migraine 1	CACNA1A
602481	Familial Hemiplegic Migraine 2	ATP1A2
609634	Familial Hemiplegic Migraine 3	SCN1A
211500	Fazio-Londe Disease	SLC52A3
607341	Focal Cortical Dysplasia of Taylor	TSC1
227260	Focal Facial Dermal Dysplasia 3	TWIST2
614974	Focal Facial Dermal Dysplasia 4	CYP26C1
614744	Hereditary Congenital Facial Paresis 3	HOXB1
614782	Hereditary Essential Tremor 4	FUS
616736	Hereditary Essential Tremor 5	TENM4
603689	Hereditary Myopathy with Early Respiratory Failure	TTN
600462	Myopathy, lactic acidosis, and sideroblastic anemia 1	PUS1
253310	Lethal Congenital Contracture Syndrome 1	GLE1
607598	Lethal Congenital Contracture Syndrome 2	ERBB3
611369	Lethal Congenital Contracture Syndrome 3	PIP5K1C
614915	Lethal Congenital Contracture Syndrome 4	MYBPC1
615368	Lethal Congenital Contracture Syndrome 5	DNM2
616248	Lethal Congenital Contracture Syndrome 6	ZBTB42
616286	Lethal Congenital Contracture Syndrome 7	CNTNAP1
616287	Lethal Congenital Contracture Syndrome 8	ADCY6
614388	Lethal Encephalopathy due to Defective Mitochondrial Peroxisomal Fission	DNM1L
612951	Cystic Leukoencephalopathy without Megalencephaly	RNASET2
616531	Polymicrogyria, Perisylvian, With Cerebellar Hypoplasia And Arthrogryposis	PI4KA
251290	Band-Like Calcification with Simplified Gyration and Polymicrogyria	OCLN
614833	Polymicrogyria with Seizures	RTTN
615752	Bilateral Perisylvian Polymicrogyria	ADGRG1
613180	Polymicrogyria with Optic Nerve Hypoplasia	TUBA8
606854	bilateral frontoparietal polymicrogyria	ADGRG1
234200	Neurodegeneration with brain iron accumulation 1	PANK2
256600	Neurodegeneration with brain iron accumulation 2A	PLA2G6
610217	Neurodegeneration with brain iron accumulation 2B	PLA2G6
606159	Neurodegeneration with brain iron accumulation 3	FTL
614298	Neurodegeneration with brain iron accumulation 4(Mitochondrial Membrane Protein-Associated Neurodegeneration)	C19orf12
300894	Neurodegeneration with brain iron accumulation 5	WDR45
615643	Neurodegeneration with brain iron accumulation 6	COASY
256000	Leigh Syndrome	BCS1L, NDUFA10, SDHA,NDUFS4, NDUFAF2, NDUFA2, NDUFAF6, SURF1, COX15, NDUFS3, NDUFS8, FOXRED1, NDUFA9, NDUFA12, COX10, NDUFS7
220111	Leigh Syndrome, French-Canadian Type	LRPPRC
613834	Multisystemic Smooth Muscle Dysfunction Syndrome	ACTA2
614399	Early-Onset Myopathy, Areflexia, Respiratory Distress, and Dysphagia	MEGF10
255600	Myosclerosis, Autosomal Recessive	COL6A2
613820	Nephronophthisis 12	TTC21B
614844	Nephronophthisis 14	ZNF423
613068	Neurodegeneration due to Cerebral Folate Transport Deficiency	FOLR1
213600	Idiopathic Basal Ganglia Calcification 1	SLC20A2
615007	Idiopathic Basal Ganglia Calcification 4	PDGFRB
615483	Idiopathic Basal Ganglia Calcification 5	PDGFB
616413	Idiopathic Basal Ganglia Calcification 6	XPR1
225790	Proliferative Vasculopathy And Hydranencephaly-Hydrocephaly Syndrome	FLVCR2
614498	Lethal Neonatal Rigidity and Multifocal Seizure Syndrome	BRAT1
180800	Roussy-Levy Syndrome	MPZ, PMP22
162091	Schwannomatosis 1	SMARCB1
615670	Schwannomatosis 2	LZTR1
602081	Speech-Language Disorder 1	FOXP2
108120	Distal Arthrogryposis type 1A	TPM2
614335	Arthrogryposis, Distal, Type 1B	MYBPC1
601680	Distal Arthrogryposis type 2B	TPM2, TNNI2, MYH3, TNNT3
193700	Distal Arthrogryposis type 2A	MYH3
114300	Distal Arthrogryposis type 3	PIEZO2
108145	Distal Arthrogryposis type 5	PIEZO2
615065	Distal Arthrogryposis type 5D	ECEL1
158300	Distal Arthrogryposis type 7	MYH8
178110	Distal Arthrogryposis type 8	MYH3
604454	Welander Distal Myopathy	TIA1
607483	Biotin-Responsive Basal Ganglia Disease	SLC19A3
614160	Myostatin-Related Muscle Hypertrophy	MSTN
607459	Mitochondrial recessive ataxia syndrome	POLG
615491	Childhood-Onset Neurodegeneration With Optic Atrophy	UCHL1
608984	Autosomal Dominant Sensory Ataxia 1	RNF170
613680	Beaulieu-Boycott-Innes Syndrome	THOC6
158810	Bethlem Myopathy 1	COL6A1, COL6A2, COL6A3
616471	Bethlem Myopathy 2	COL12A1
115430	Carpal Tunnel Syndrome	TTR
601338	Cerebellar Ataxia, Areflexia, Pes Cavus, Optic Atrophy, And Sensorineuralhearing Loss	ATP1A3
614756	Nonprogressive Cerebellar Ataxia With Mental Retardation	CAMTA1
616202	Cerebellofaciodental Syndrome	BRF1
606703	Familial Dyskinesia With Facial Myokymia	ADCY5
616921	Infantile-Onset Limb And Orofacial Dyskinesia	PDE10A
615924	Encephalopathy, Progressive, With Or Without Lipodystrophy	BSCL2
605013	Microhydranencephaly	NDE1
610100	Giant Axonal Neuropathy 2, Autosomal Dominant	DCAF8
212840	Gordon Holmes Syndrome	RNF216
139393	Familial Guillain-Barre Syndrome	PMP22
615281	Hypomyelination With Brainstem And Spinal Cord Involvement And Legspasticity	DARS
615422	Inclusion Body Myopathy With Early-Onset Paget Disease With Or Without frontotemporal Dementia 2	HNRNPA2B1
615424	Inclusion Body Myopathy With Early-Onset Paget Disease With Or Without frontotemporal Dementia 3	HNRNPA1
612713	Kahrizi Syndrome	SRD5A3
245800	Laurence-Moon Syndrome	PNPLA6
615889	Progressive Leukoencephalopathy With Ovarian Failure	AARS2
250100	Metachromatic Leukodystrophy	ARSA
613353	Mild Mononeuropathy Of The Median Nerve	SH3TC2
614937	Familial Cortical Myoclonus	NOL3
615673	Myopathy With Extrapyramidal Signs	MICU1
614369	Peripheral Neuropathy, Myopathy, Hoarseness, And Hearing Loss	MYH14
615895	Polyglucosan Body Myopathy 1 With Or Without Immunodeficiency	RBCK1
616199	Polyglucosan Body Myopathy 2	GYG1
612691	Polymicrogyria, Bilateral Temporooccipital	FIG4
616081	Pontocerebellar Hypoplasia, Type 1C	EXOSC8
615851	Pontocerebellar Hypoplasia, Type 2E	VPS53
606353	Primary Lateral Sclerosis, Juvenile	ALS2
157640	Progressive External Ophthalmoplegia With Mitochondrial Dna Deletions, autosomal dominant 1	POLG
606072	Rippling Muscle Disease	CAV3
181405	Scapuloperoneal Spinal Muscular Atrophy	TRPV4
607225	Infantile-Onset Ascending Spastic Paralysis	ALS2
606688	Spongiform Encephalopathy With Neuropsychiatric Features	PRNP
609161	Striatal Degeneration, Autosomal Dominant 1	PDE8B
616922	Striatal Degeneration, Autosomal Dominant 2	PDE10A
271930	Striatonigral Degeneration, Infantile	NUP62
601104	Progressive Supranuclear Palsy 1	MAPT
218340	Temtamy Syndrome	C12orf57
155310	Visceral Myopathy	ACTG2
314580	Wieacker-Wolff Syndrome	ZC4H2
615217	Ataxia-oculomotor apraxia 3	PIK3R5
616421	Myoclonic-atonic epilepsy	SLC6A1
300960	Mend Syndrome	EBP
616852	Scapulohumeroperoneal Myopathy	ACTA1
616866	Spinal Muscular Atrophy With Congenital Bone Fractures 1	TRIP4
616867	Spinal Muscular Atrophy With Congenital Bone Fractures 2	ASCC1
617158	Myopathy, distal, with rimmed vacuoles	SQSTM1
NA005	Charcot-Marie-Tooth disease type 2	BSCL2
NA006	MYH7-Related Congenital Fiber-type Disproportion	MYH7
NA007	TPM2-Related Congenital Fiber-type Disproportion	TPM2
NA008	RYR1-Related Congenital Fiber-type Disproportion	RYR1
NA017	X-Linked Leigh Syndrome	PDHA1
NA024	Peripheral Neuropathy with sensory symptoms	HARS
NA025	DNAJB6-Related Myofibrillar Myopathy	DNAJB6
NA026	FHL1-Related Myofibrillar Myopathy	FHL1
NA029	Lennox-Gastaut Syndrome	MAPK10
NA031	Childhood Myocerebrohepatopathy Spectrum	POLG
NA032	L1 syndrome	L1CAM
617435	Lopes-Maciel-Rodan syndrome	HTT
617493	Neurodevelopmental disorder with involuntary movements	GNAO1
617145	Neurodegeneration with ataxia, dystonia, and gaze palsy, childhood-onset	SQSTM1
617146	Arthrogryposis, distal, with impaired proprioception and touch	PIEZO2
617207	Encephalopathy, progressive, with amyotrophy and optic atrophy	TBCE
617235	Myoclonus, intractable, neonatal	KIF5A
269880	SHORT Syndrome	PIK3R1
168400	Parastremmatic Dwarfism	TRPV4
187601	Thanatophoric Dysplasia, type II	FGFR3
616462	Acrofacial dysostosis, Cincinnati type	POLR1A
156550	Kniest dysplasia	COL2A1
238320	Leydig cell hypoplasia	LHCGR
612287	Hypophosphatemic Nephrolithiasis/Osteoporosis 2	SLC9A3R1
163950	Noonan Syndrome 1	PTPN11
609942	Noonan Syndrome 3	KRAS
610733	Noonan Syndrome 4	SOS1
611553	Noonan Syndrome 5	RAF1
613224	Noonan Syndrome 6	NRAS
613706	Noonan Syndrome 7	BRAF
615355	Noonan Syndrome 8	RIT1
616559	Noonan Syndrome 9	SOS2
616564	Noonan Syndrome 10	LZTR1
613563	Noonan-Like Syndrome Disorder with or without Juvenile Myelomonocytic Leukemia	CBL
607721	Noonan-Like Syndrome with Loose Anagen Hair 1	SHOC2
214500	Chediak-Higashi Syndrome	LYST
203300	Hermansky-Pudlak Syndrome 1	HPS1
608233	Hermansky-Pudlak Syndrome 2	AP3B1
614072	Hermansky-Pudlak Syndrome 3	HPS3
614073	Hermansky-Pudlak Syndrome 4	HPS4
614074	Hermansky-Pudlak Syndrome 5	HPS5
614075	Hermansky-Pudlak Syndrome 6	HPS6
614076	Hermansky-Pudlak Syndrome 7	DTNBP1
614171	Hermansky-Pudlak Syndrome 9	BLOC1S6
121050	Congenital Contractural Arachnodactyly	FBN2
154500	Treacher Collins Syndrome 1	TCOF1
613717	Treacher Collins Syndrome 2	POLR1D
248390	Treacher Collins Syndrome 3	POLR1C
277600	Weill-Marchesani Syndrome 1	ADAMTS10
608328	Weill-Marchesani Syndrome 2	FBN1
614819	Weill-Marchesani Syndrome 3	LTBP2
129490	Autosomal Recessive Hypohidrotic Ectodermal Dysplasia	EDAR
614940	Autosomal Dominant Hypohidrotic Ectodermal Dysplasia	EDARADD
604536	Ectodermal Dysplasia/Skin Fragility Syndrome	PKP1
305100	X-Linked Hypohidrotic Ectodermal Dysplasia	EDA
129500	Clouston syndrome	GJB6
602032	Ectodermal Dysplasia 4, Hair/Nail type	KRT85
614929	Ectodermal Dysplasia 7, Hair/Nail type	KRT74
614931	Ectodermal Dysplasia 9, Hair/Nail type	HOXC13
224900	Ectodermal Dysplasia 10b, Hypohidrotic/Hair/Tooth type	EDAR
614941	Ectodermal Dysplasia 11b, Hypohidrotic/Hair/Tooth type	EDARADD
616029	Ectodermal Dysplasia/Short Stature Syndrome	GRHL2
613573	Ectodermal Dysplasia-Syndactyly Syndrome 1	PVRL4
616657	Spastic Tetraplegia, Thin Corpus Callosum, And Progressive Microcephaly	SLC1A4
209900	Bardet-Biedl Syndrome 1	BBS1
615981	Bardet-Biedl Syndrome 2	BBS2
600151	Bardet-Biedl Syndrome 3	ARL6
615982	Bardet-Biedl Syndrome 4	BBS4
615983	Bardet-Biedl Syndrome 5	BBS5
605231	Bardet-Biedl Syndrome 6	MKKS
615984	Bardet-Biedl Syndrome 7	BBS7
615985	Bardet-Biedl Syndrome 8	TTC8
615986	Bardet-Biedl Syndrome 9	BBS9
615987	Bardet-Biedl Syndrome 10	BBS10
615988	Bardet-Biedl Syndrome 11	TRIM32
615989	Bardet-Biedl Syndrome 12	BBS12
615990	Bardet-Biedl Syndrome 13	MKS1
615991	Bardet-Biedl Syndrome 14	CEP290
615992	Bardet-Biedl Syndrome 15	WDPCP
615993	Bardet-Biedl Syndrome 16	SDCCAG8
615994	Bardet-Biedl Syndrome 17	LZTFL1
615995	Bardet-Biedl Syndrome 18	BBIP1
615996	Bardet-Biedl Syndrome 19	IFT27
617406	Bardet-Biedl syndrome 21	C8orf37
157170	Holoprosencephaly 2	SIX3
142945	Holoprosencephaly 3	SHH
142946	Holoprosencephaly 4	TGIF1
609637	Holoprosencephaly 5	ZIC2
610828	Holoprosencephaly 7	PTCH1
610829	Holoprosencephaly 9	GLI2
614226	Holoprosencephaly 11	CDON
260400	Shwachman-Diamond Syndrome	SBDS
243800	Johanson-Blizzard Syndrome	UBR1
164200	Oculodentodigital Dysplasia	GJA1
214800	CHARGE syndrome	CHD7, SEMA3E
216400	Cockayne Syndrome A	ERCC8
133540	Cockayne Syndrome B	ERCC6
605432	Radioulnar Synostosis with Amegakaryocytic Thrombocytopenia 1	HOXA11
616738	Radioulnar Synostosis With Amegakaryocytic Thrombocytopenia 2	MECOM
273750	3-M Syndrome 1	CUL7
612921	3-M Syndrome 2	OBSL1
614205	3-M Syndrome 3	CCDC8
268310	Autosomal Recessive Robinow Syndrome	ROR2
180700	Robinow syndrome, autosomal dominant 1	WNT5A
214450	Griscelli Syndrome 1	MYO5A
607624	Griscelli Syndrome 2	RAB27A
609227	Griscelli Syndrome 3	MLPH
251200	Primary Autosomal Recessive Microcephaly 1	MCPH1
604317	Primary Autosomal Recessive Microcephaly 2, With Or Without Corticalmalformations	WDR62
604804	Primary Autosomal Recessive Microcephaly 3	CDK5RAP2
604321	Primary Autosomal Recessive Microcephaly 4	CASC5
608716	Primary Autosomal Recessive Microcephaly 5	ASPM
608393	Primary Autosomal Recessive Microcephaly 6	CENPJ
612703	Primary Autosomal Recessive Microcephaly 7	STIL
614673	Primary Autosomal Recessive Microcephaly 8	CEP135
614852	Primary Autosomal Recessive Microcephaly 9	CEP152
615095	Primary Autosomal Recessive Microcephaly 10	ZNF335
616080	Primary Autosomal Recessive Microcephaly 12	CDK6
616051	Primary Autosomal Recessive Microcephaly 13	CENPE
616402	Primary Autosomal Recessive Microcephaly 14	SASS6
607196	Amish Lethal Microcephaly	SLC25A19
251270	Autosomal Recessive Microcephaly And Chorioretinopathy 1	TUBGCP6
616171	Autosomal Recessive Microcephaly And Chorioretinopathy 2	PLK4
152950	Microcephaly With Or Without Chorioretinopathy, Lymphedema, Or Mental retardation	KIF11
613668	Postnatal Progressive Microcephaly With Seizures And Brain Atrophy	MED17
615760	Progressive Microcephaly With Seizures And Cerebral And Cerebellaratrophy	QARS
616541	Short Stature, Microcephaly, And Endocrine Dysfunction	XRCC4
610536	Mandibulofacial Dysostosis with Microcephaly	EFTUD2
614231	Microcephaly, Epilepsy, and Diabetes Syndrome	IER3IP1
614261	Microcephaly-Capillary Malformation Syndrome	STAMBP
253290	Multiple Pterygium Syndrome,lethal type	CHRNA1, CHRND, CHRNG
265000	Multiple Pterygium Syndrome,nonlethal type	CHRNG
122470	Cornelia de Lange syndrome 1	NIPBL
300590	Cornelia de Lange syndrome 2	SMC1A
610759	Cornelia de Lange syndrome 3	SMC3
614701	Cornelia de Lange syndrome 4	RAD21
300882	Cornelia de Lange syndrome 5	HDAC8
203800	Alstrom Syndrome	ALMS1
106260	Ankyloblepharon-Ectodermal Defects-Cleft Lip/palate syndrome	TP63
210900	Bloom Syndrome	BLM
113620	Branchiooculofacial Syndrome	TFAP2A
169100	Char Syndrome	TFAP2B
602849	Muenke Syndrome	FGFR3
308050	Congenital Hemidysplasia with Ichthyosiform Erythroderma and Limb Defects	NSDHL
251260	Nijmegen Breakage Syndrome	NBN
613078	Nijmegen Breakage Syndrome-like Disorder	RAD50
311200	Oral-Facial-Digital Syndrome	OFD1
268300	Roberts Syndrome	ESCO2
614753	Sotos Syndrome 2	NFIX
273395	Tetra-Amelia Syndrome	WNT3
107480	Townes-Brocks Syndrome 1	SALL1
277700	Werner Syndrome	WRN
304110	Craniofrontonasal syndrome	EFNB1
312870	Simpson-Golabi-Behmel Syndrome Type 1	GPC3
300209	Simpson-Golabi-Behmel Syndrome Type 2	OFD1
216550	Cohen Syndrome	VPS13B
136140	Floating-Harbor Syndrome	SRCAP
176670	Hutchinson-Gilford Progeria Syndrome	LMNA
147920	Kabuki Syndrome 1	KMT2D
300867	Kabuki syndrome 2	KDM6A
309000	Lowe Syndrome	OCRL
248450	Manitoba Oculotrichoanal Syndrome	FREM1
236700	McKusick-Kaufman Syndrome	MKKS
231550	Achalasia-Addisonianism-Alacrima Syndrome	AAAS
609242	Kanzaki Disease	NAGA
169400	Pelger-Huet Anomaly	LBR
164280	Feingold syndrome 1	MYCN
146510	Pallister-Hall Syndrome	GLI3
276950	VACTERL association with hydrocephalus	PTEN
314390	X-linked VACTERL syndrome with or without hydrocephalus	ZIC3
153480	Bannayan-Riley-Ruvalcaba syndrome	PTEN
243310	Baraitser-Winter Syndrome 1	ACTB
614583	Baraitser-Winter Syndrome 2	ACTG1
609460	Goldberg-Shprintzen syndrome	KIAA1279
175700	Greig cephalopolysyndactyly syndrome	GLI3
119300	Van der Woude syndrome 1	IRF6
119500	Popliteal pterygium syndrome	IRF6
263650	Popliteal Pterygium Syndrome, Lethal Type	RIPK4
201000	Carpenter syndrome 1	RAB23
614976	Carpenter Syndrome 2	MEGF8
607313	Horizontal gaze palsy with progressive scoliosis 1	ROBO3
235730	Mowat-Wilson syndrome	ZEB2
263750	Miller syndrome	DHODH
149730	Lacrimoauriculodentodigital Syndrome	FGFR2, FGFR3, FGF10
259770	Osteoporosis-pseudoglioma syndrome	LRP5
269150	Schinzel-Giedion syndrome	SETBP1
184460	Stapes Ankylosis with Broad Thumb and Toes	NOG
215470	Boucher-Neuhauser syndrome	PNPLA6
118450	Alagille Syndrome 1	JAG1
610205	Alagille Syndrome 2	NOTCH2
305400	Aarskog-Scott syndrome	FGD1
100300	Adams-Oliver Syndrome 1	ARHGAP31
614219	Adams-Oliver Syndrome 2	DOCK6
614814	Adams-Oliver Syndrome 3	RBPJ
615297	Adams-Oliver Syndrome 4	EOGT
616028	Adams-Oliver Syndrome 5	NOTCH1
616589	Adams-Oliver Syndrome 6	DLL4
306955	Visceral Heterotaxy 1	ZIC3
613751	Visceral Heterotaxy 4	ACVR2B
270100	Visceral Heterotaxy 5	NODAL
614779	Visceral Heterotaxy 6	CCDC11
616749	Visceral Heterotaxy 7	MMP21
249000	Meckel syndrome 1	MKS1
603194	Meckel syndrome 2	TMEM216
607361	Meckel syndrome 3	TMEM67
611134	Meckel syndrome 4	CEP290
611561	Meckel syndrome 5	RPGRIP1L
612284	Meckel syndrome 6	CC2D2A
267010	Meckel syndrome 7	NPHP3
613885	Meckel syndrome 8	TCTN2
614209	Meckel syndrome 9	B9D1
614175	Meckel syndrome 10	B9D2
615397	Meckel syndrome 11	TMEM231
616258	Meckel syndrome 12	KIF14
214150	Cerebrooculofacioskeletal Syndrome 1	ERCC6
610756	Cerebrooculofacioskeletal Syndrome 2	ERCC2
616570	Cerebrooculofacioskeletal Syndrome 3	ERCC5
610758	Cerebrooculofacioskeletal Syndrome 4	ERCC1
303400	X-Linked Cleft Palate with or without Ankyloglossia	TBX22
607842	Congenital Aural Atresia	TSHZ1
161200	Nail-Patella Syndrome	LMX1B
603041	Mitochondrial DNA depletion syndrome 1	TYMP
609560	Mitochondrial DNA depletion syndrome 2	TK2
251880	Mitochondrial DNA depletion syndrome 3	DGUOK
203700	Mitochondrial DNA depletion syndrome 4A	POLG
613662	Mitochondrial DNA depletion syndrome 4B	POLG
256810	Mitochondrial DNA depletion syndrome 6	MPV17
612075	Mitochondrial DNA depletion syndrome 8A	RRM2B
212350	Mitochondrial DNA depletion syndrome 10	AGK
615084	Mitochondrial DNA depletion syndrome 11	MGME1
615418	Mitochondrial DNA depletion syndrome 12	SLC25A4
615471	Mitochondrial DNA depletion syndrome 13	FBXL4
616896	Mitochondrial DNA Depletion Syndrome 14	OPA1
208150	Akinesia Deformation Sequence	RAPSN, DOK7, MUSK
604292	Ectrodactyly, Ectodermal Dysplasia, Clefting Syndrome 3	TP63
225280	Ectodermal dysplasia, Ectrodactyly, and macular dystrophy Syndrome	CDH3
612714	Exocrine Pancreatic Insufficiency, Dyserythropoietic Anemia, and Calvarial Hyperostosis	COX4I2
219000	Fraser Syndrome	GRIP1, FRAS1, FREM2
249420	Frank-ter Haar Syndrome	SH3PXD2B
612938	Growth Retardation, Developmental Delay, Coarse Facies, and Early Death	FTO
611174	Hamamy Syndrome	IRX5
611773	Hereditary Angiopathy with Nephropathy, Aneurysms, and Muscle Cramps	COL4A1
613561	Myopathy, Lactic acidosis, and Sideroblastic anemia 2	YARS2
602782	Histiocytosis-Lymphadenopathy Plus Syndrome	SLC29A3
236680	Hydrolethalus Syndrome 1	HYLS1
614120	Hydrolethalus Syndrome 2	KIF7
616483	Infantile liver failure syndrome 2	NBAS
217080	Jalili Syndrome	CNNM4
251255	Jawad Syndrome	RBBP8
148050	KBG Syndrome	ANKRD11
244460	Kenny-Caffey Syndrome Type 1	TBCE
127000	Kenny-Caffey Syndrome Type 2	FAM111A
226750	Kohlschutter-Tonz Syndrome	ROGDI
616503	Lethal Congenital Contracture Syndrome 9	GPR126
603543	Limb-Mammary Syndrome	TP63
604308	MASS Syndrome	FBN1
615937	Megalencephaly-polymicrogyria-polydactyly-hydrocephalus syndrome 2	AKT3
224690	Meier-Gorlin Syndrome 1	ORC1
613800	Meier-Gorlin Syndrome 2	ORC4
613803	Meier-Gorlin Syndrome 3	ORC6
613804	Meier-Gorlin Syndrome 4	CDT1
613805	Meier-Gorlin Syndrome 5	CDC6
616835	Meier-Gorlin Syndrome 6	GMNN
604273	Mitochondrial Complex V (ATP Synthase) Deficiency, Nuclear Type 1	ATPAF2
614052	Mitochondrial Complex V (ATP Synthase) Deficiency, Nuclear Type 2	TMEM70
614053	Mitochondrial Complex V (ATP Synthase) Deficiency, Nuclear Type 3	ATP5E
615228	Mitochondrial Complex V (ATP Synthase) Deficiency, Nuclear Type 4	ATP5A1
241080	Woodhouse-Sakati syndrome	DCAF17
252010	Mitochondrial complex I deficiency	NDUFB3, NDUFS1, NDUFAF3, NDUFS6, NDUFS4, NDUFAF2, NDUFAF4, NDUFB9, NDUFS3, NDUFV1, FOXRED1, NUBPL, NDUFAF1, NDUFV2, NDUFA11, NDUFAF5, NDUFA1
252011	Mitochondrial complex II deficiency	SDHA, SDHD, SDHAF1
615157	Mitochondrial complex III deficiency nuclear type 2	TTC19
615158	Mitochondrial complex III deficiency nuclear type 3	UQCRB
615159	Mitochondrial complex III deficiency nuclear type 4	UQCRQ
615160	Mitochondrial complex III deficiency nuclear type 5	UQCRC2
615453	Mitochondrial complex III deficiency nuclear type 6	CYC1
615824	Mitochondrial complex III deficiency nuclear type 7	UQCC2
615838	Mitochondrial complex III deficiency nuclear type 8	LYRM7
616111	Mitochondrial complex III deficiency nuclear type 9	UQCC3
220110	Mitochondrial complex IV deficiency	COX20, COA5, FASTKD2, COX14, APOPT1, COX10, TACO1, COX6B1, PET100
257300	Mosaic variegated aneuploidy syndrome 1	BUB1B
614114	Mosaic variegated aneuploidy syndrome 2	CEP57
253250	Mulibrey nanism	TRIM37
605711	Multiple mitochondrial dysfunctions syndrome 1	NFU1
615330	Multiple mitochondrial dysfunctions syndrome 3	IBA57
616370	Multiple mitochondrial dysfunctions syndrome 4	ISCA2
161000	Naegeli-Franceschetti-Jadassohn Syndrome	KRT14
609981	Natural Killer Cell and Glucocorticoid Deficiency with DNA Repair Defect	MCM4
601214	Naxos Disease	JUP
614008	Nestor-Guillermo Progeria Syndrome	BANF1
601321	Neurofibromatosis-Noonan Syndrome	NF1
181400	Neurogenic Scapuloperoneal Syndrome, Kaeser Type	DES
190440	Trigonocephaly 1	FGFR1
614485	Trigonocephaly 2	FREM1
257980	Odontoonychodermal Dysplasia	WNT10A
300855	Ogden Syndrome	NAA10
217085	Congenital Heart Defects, Hamartomas Of Tongue, And Polysyndactyly	WDPCP
614980	Multiple Types Congenital Heart Defects 2	TAB2
615779	Multiple Types Congenital Heart Defects 4	NR2F2
615710	Mitchell-Riley syndrome	RFX6
245000	Papillon-Lefevre Syndrome	CTSC
267000	Perlman Syndrome	DIS3L2
176920	Proteus Syndrome	AKT1
208540	Renal-Hepatic-Pancreatic Dysplasia 1	NPHP3
611943	RIDDLE Syndrome	RNF168
269000	SC Phocomelia Syndrome	ESCO2
224750	Schopf-Schulz-Passarge Syndrome	WNT10A
601559	Schwartz-Jampel Syndrome, Type 2	LIFR
225500	Ellis-van Creveld Syndrome	EVC2, EVC
182212	Shprintzen-Goldberg Craniosynostosis Syndrome	SKI
615703	Morbid Obesity And Spermatogenic Failure	CEP19
311900	TARP Syndrome	RBM10
187500	Tetralogy of Fallot	NKX2-5, GATA4, ZFPM2, GDF1, GATA6, TBX1, JAG1
190350	Trichorhinophalangeal Syndrome	TRPS1
208085	Arthrogryposis, Renal Dysfunction, and Cholestasis Syndrome 1	VPS33B
613404	Arthrogryposis, Renal Dysfunction, and Cholestasis Syndrome 2	VIPAS39
611890	Lethal Arthrogryposis With Anterior Horn Cell Disease	GLE1
181450	Ulnar-Mammary Syndrome	TBX3
606713	Van der Woude Syndrome 2	GRHL3
242840	Vici Syndrome	EPG5
277610	Weissenbacher-Zweymuller Syndrome	COL11A2
103285	ADULT syndrome	TP63
202650	Agnathia-Otocephaly Complex	PRRX1
257920	3MC Syndrome 1	MASP1
265050	3MC Syndrome 2	COLEC11
256040	Autoinflammation, Lipodystrophy, and Dermatosis Syndrome	PSMB8
605039	Bohring-Opitz Syndrome	ASXL1
208250	Camptodactyly-Arthropathy-Coxa Vara-Pericarditis Syndrome	PRG4
608874	Orofacial Cleft 5	MSX1
225060	Orofacial cleft 7	PVRL1
129400	Orofacial Cleft 8	TP63
613705	Orofacial Cleft 10	SUMO1
600625	Orofacial Cleft 11	BMP4
616788	Orofacial Cleft 15	DLX4
616373	Pulmonary fibrosis and/or bone marrow failure, telomere-related, 3	RTEL1
616371	Pulmonary fibrosis and/or bone marrow failure, telomere-related, 4	PARN
615465	Hartsfield Syndrome	FGFR1
615485	Bainbridge-Ropers Syndrome	ASXL3
302905	Abruzzo-Erickson Syndrome	TBX22
200500	Acheiropody	LMBR1
615071	Alazami Syndrome	LARP7
612079	Alopecia, Neurologic Defects, And Endocrinopathy Syndrome	RBM28
201750	Antley-Bixler Syndrome With Genital Anomalies And Disordered Steroidogenesis	POR
207410	Antley-Bixler Syndrome Without Genital Anomalies Or Disordered Steroidogenesis	FGFR2
616192	Ataxia, Combined Cerebellar And Peripheral, With Hearing Loss And diabetes Mellitus	DNAJC3
608980	Bifid Nose With Or Without Anorectal And Renal Anomalies	FREM1
616001	Breasts And/Or Nipples, Aplasia Or Hypoplasia Of, 2	PTPRF
608572	Burn-Mckeown Syndrome	TXNL4A
616145	Catel-Manzke Syndrome	TGDS
117650	Cerebrocostomandibular Syndrome	SNRPB
613611	Choanal Atresia And Lymphedema	PTPN14
300863	Chondrodysplasia With Platyspondyly, Distinctive Brachydactyly, Hydrocephaly,And Microphthalmia	HDAC6
616201	Chronic Atrial And Intestinal Dysrhythmia	SGOL1
613630	Cocoon Syndrome	CHUK
600373	CODAS Syndrome	LONP1
616266	Congenital Contractures Of The Limbs And Face, Hypotonia, And Developmental delay	NALCN
614115	Cortical Malformations, Occipital	LAMC3
614195	Craniofacial Anomalies And Anterior Segment Dysgenesis Syndrome	VSX1
607812	Craniolenticulosutural Dysplasia	SEC23A
278800	De Sanctis-Cacchione Syndrome	ERCC6
612651	Endocrine-Cerebroosteodysplasia	ICK
600251	Facial Clefting, Oblique, 1	SPECC1L
615139	Facial Dysmorphism, Immunodeficiency, Livedo, And Short Stature	POLE
601552	Facial Dysmorphism, Lens Dislocation, Anterior Segment Abnormalities,And Spontaneous Filtering Blebs	ASPH
272440	Filippi Syndrome	CKAP2L
609218	Foveal Hypoplasia 2	SLC38A8
230740	GAPO Syndrome	ANTXR1
605130	Hairy Elbows, Short Stature, Facial Dysmorphism, And Developmental delay	KMT2A
610140	Heart-Hand Syndrome, Slovenian Type	LMNA
300537	Heterotopia, Periventricular, Ehlers-Danlos Variant	FLNA
613845	Hyperuricemia, Pulmonary Hypertension, Renal Failure, And Alkalosis syndrome	SARS2
615419	Hypotonia, Infantile, With Psychomotor Retardation And Characteristic facies 1	NALCN
616801	Hypotonia, Infantile, With Psychomotor Retardation And Characteristic facies 2	UNC80
616900	Hypotonia, Infantile, With Psychomotor Retardation And Characteristic facies 3	TBCK
616816	Hypotonia, Infantile, With Psychomotor Retardation	CCDC174
601553	Congenital Hypotrichosis with Juvenile Macular Dystrophy	CDH3
614748	Congenital Interstitial Lung Disease, Nephrotic Syndrome, And Epidermolysis Bullosa	ITGA3
614098	Keppen-Lubinsky Syndrome	KCNJ6
135750	Laurin-Sandrow Syndrome	LMBR1
614192	Macrocephaly, Macrosomia, And Facial Dysmorphism Syndrome	RNF135
248000	Autosomal Recessive Macrocephaly/Megalencephaly Syndrome	TBC1D7
248700	Marden-Walker Syndrome	PIEZO2
602535	Marshall-Smith Syndrome	NFIX
615938	Megalencephaly-Polymicrogyria-Polydactyly-Hydrocephalus Syndrome 3	CCND2
614080	Multiple Congenital Anomalies-Hypotonia-Seizures Syndrome 1	PIGN
615398	Multiple Congenital Anomalies-Hypotonia-Seizures Syndrome 3	PIGT
256520	Neu-Laxova Syndrome 1	PHGDH
616038	Neu-Laxova Syndrome 2	PSAT1
616263	Infantile-Onset Multisystem Neurologic, Endocrine, And Pancreatic Disease	PTRH2
613886	Obesity, Hyperphagia, And Developmental Delay	NTRK2
257850	Autosomal Recessive Oculodentodigital Dysplasia	GJA1
275400	Oliver-Mcfarlane Syndrome	PNPLA6
300000	Opitz Gbbb Syndrome, Type I	MID1
258860	Orofaciodigital Syndrome IV	TCTN3
174300	Orofaciodigital Syndrome V	DDX59
277170	Orofaciodigital Syndrome VI	C5orf42
615948	Orofaciodigital Syndrome XIV	C2CD3
604715	Orthostatic Intolerance	SLC6A2
606721	Partial Lipodystrophy, Congenital Cataracts, And Neurodegeneration syndrome	CAV1
615704	Poikiloderma, Hereditary Fibrosing, With Tendon Contractures, Myopathy,And Pulmonary Fibrosis	FAM111B
616113	Polyendocrine-Polyneuropathy Syndrome	DMXL2
611087	Polyhydramnios, Megalencephaly, And Symptomatic Epilepsy	STRADA
614501	Psychomotor Retardation, Epilepsy, And Craniofacial Dysmorphism	SNIP1
612798	Question Mark Ears, Isolated	EDN1
613471	Reynolds Syndrome	LBR
268305	Richieri Costa-Pereira syndrome	EIF4A3
220210	Ritscher-Schinzel Syndrome 1	KIAA0196
300963	Ritscher-Schinzel Syndrome 2	CCDC22
616200	Ruijs-Aalfs Syndrome	SPRTN
615789	Short Stature With Microcephaly And Distinctive Facies	CRIPT
614813	Short Stature, Onychodysplasia, Facial Dysmorphism, And Hypotrichosis	POC1A
614800	Short Stature, Optic Nerve Atrophy, And Pelger-Huet Anomaly	NBAS
609508	Stickler Syndrome, Type I, Nonsyndromic Ocular	COL2A1
608800	Sudden Infant Death With Dysgenesis Of The Testes Syndrome	TSPYL1
615542	Testicular Anomalies With Or Without Congenital Heart Disease	GATA4
190320	Trichodentoosseous Syndrome	DLX3
190351	Trichorhinophalangeal Syndrome, Type III	TRPS1
219730	Ventriculomegaly With Cystic Kidney Disease	CRB2
613398	Warsaw Breakage Syndrome	DDX11
615926	Webb-Dattani Syndrome	ARNT2
216340	Yunis-Varon Syndrome	FIG4
135500	Zimmermann-Laband syndrome 1	KCNH1
616455	Zimmermann-Laband syndrome 2	ATP6V1B2
112240	Cole-Carpenter syndrome 1	P4HB
616294	Cole-Carpenter syndrome 2	SEC24D
112410	Hypertension and brachydactyly syndrome	PDE3A
182250	Singleton-Merten syndrome 1	IFIH1
616298	Singleton-Merten syndrome 2	DDX58
200110	Ablepharon-macrostomia syndrome	TWIST2
209885	Barber-Say syndrome	TWIST2
605822	Spondyloocular syndrome	XYLT2
616367	Mandibulofacial dysostosis with alopecia	EDNRA
616368	CHOPS syndrome	AFF4
616459	Al-Raqad syndrome	DCPS
616482	Severe achondroplasia with developmental delay and acanthosis nigricans	FGFR3
616489	Severe Growth restriction with distinctive facies	IGF2
210000	BEHR syndrome	OPA1
607131	Al-Gazali-Bakalinova Syndrome	KIF7
616875	Cerebellar Atrophy, Visual Impairment, And Psychomotor Retardation	EMC1
616728	Cleft Palate, Psychomotor Retardation, And Distinctive Facial Features	KDM1A
616901	Developmental Delay With Short Stature, Dysmorphic Features, And Sparse Hair	DPH1
616577	Epilepsy, Hearing Loss, And Mental Retardation Syndrome	SPATA5
616854	Even-Plus Syndrome	HSPA9
263210	Gillessen-Kaesbach-Nishimura Syndrome	ALG9
616920	Heart And Brain Malformation Syndrome	SMG9
616592	Kosaki Overgrowth Syndrome	PDGFRB
616803	Lamb-Shaffer Syndrome	SOX5
616831	Luscan-Lumish Syndrome	SETD2
616914	Marfan Lipodystrophy Syndrome	FBN1
616878	Recurrent Metabolic Encephalomyopathic Crises With Rhabdomyolysis, Cardiac Arrhythmias, And Neurodegeneration	TANGO2
602342	Pierpont Syndrome	TBL1XR1
601812	Premature Aging Syndrome, Penttinen Type	PDGFRB
616632	Seizures, Cortical Blindness, And Microcephaly Syndrome	DIAPH1
616682	Seizures, Scoliosis, And Macrocephaly Syndrome	EXT2
243605	Stromme Syndrome	CENPF
601675	Photosensitive Trichothiodystrophy 1	ERCC2
616390	Photosensitive Trichothiodystrophy 2	ERCC3
616395	Photosensitive Trichothiodystrophy 3	GTF2H5
234050	Nonphotosensitive Trichothiodystrophy 4	MPLKIP
300953	Nonphotosensitive Trichothiodystrophy 5	RNF113A
616943	Nonphotosensitive Trichothiodystrophy 6	GTF2E2
616975	Neurodevelopmental disorder with or without anomalies of the brain, eye, or heart	RERE
617053	MIRAGE syndrome	SAMD9
617101	Dias-Logan syndrome	BCL11A
617171	Dyskinesia, seizures, and intellectual developmental disorder	DEAF1
617180	Chitayat syndrome	ERF
NA001	MAP2K1-Related Noonan Syndrome	MAP2K1
NA009	FOXH1-Related Holoprosencephaly	FOXH1
NA010	NODAL-Related Holoprosencephaly	NODAL
NA011	TDGF1-Related Holoprosencephaly	TDGF1
NA013	DLL1-Related Holoprosencephaly	DLL1
NA014	FGF8-Related Holoprosencephaly	FGF8
617306	COMMAD syndrome	MITF
277180	Congenital Absence of the Vas Deferens	CFTR
608978	Meacham Syndrome	WT1
176400	Central Precocious Puberty 1	KISS1R
615346	Central Precocious Puberty 2	MKRN3
300018	46,XY Sex Reversal 2	NR0B1
612965	46,XY Sex Reversal 3	NR5A1
613080	46,XY Sex Reversal 5	CBX2
613762	46,XY Sex Reversal 6	MAP3K1
233420	46,XY Sex Reversal 7	DHH
614279	46,XY Sex Reversal 8	AKR1C2, AKR1C4
616067	46,XY Sex Reversal 9	ZFPM2
617480	46,XX Sex Reversal 4	NR5A1
611812	SERKAL syndrome	WNT4
231090	Recurrent Hydatidiform Mole 1	NLRP7
614293	Recurrent Hydatidiform Mole 2	KHDC3L
300633	X-Linked Hypospadias 1	AR
300758	X-Linked Hypospadias 2	MAMLD1
158330	Mullerian aplasia and hyperandrogenism	WNT4
608115	Ovarian Hyperstimulation Syndrome	FSHR
233400	Perrault Syndrome 1	HSD17B4
614926	Perrault Syndrome 2	HARS2
614129	Perrault Syndrome 3	CLPP
615300	Perrault Syndrome 4	LARS2
616138	Perrault Syndrome 5	C10orf2
233300	Ovarian Dysgenesis 1	FSHR
300510	Ovarian Dysgenesis 2(Premature Ovarian Failure 4)	BMP15
614324	Ovarian Dysgenesis 3	PSMC3IP
616185	Ovarian Dysgenesis 4	MCM9
300511	Premature Ovarian Failure 2A	DIAPH2
300604	Premature Ovarian Failure 2B	POF1B
608996	Premature Ovarian Failure 3	FOXL2
611548	Premature Ovarian Failure 5	NOBOX
612310	Premature Ovarian Failure 6	FIGLA
612964	Premature Ovarian Failure 7	NR5A1
615723	Premature Ovarian Failure 8	STAG3
615724	Premature Ovarian Failure 9	HFM1
612885	Premature Ovarian Failure 10	MCM8
616946	Premature Ovarian Failure 11	ERCC6
616947	Premature Ovarian Failure 12	SYCE1
309120	X-linked spermatogenic Failure 2	TEX11
606766	Spermatogenic failure 3	SLC26A8
270960	Spermatogenic failure 4	SYCP3
243060	Spermatogenic failure 5	AURKC
102530	Spermatogenic failure 6	SPATA16
612997	Spermatogenic failure 7	CATSPER1
613957	Spermatogenic failure 8	NR5A1
613958	Spermatogenic failure 9	DPY19L2
615081	Spermatogenic failure 11	KLHL10
615841	Spermatogenic failure 13	TAF4B
615842	Spermatogenic failure 14	ZMYND15
616950	Spermatogenic failure 15	SYCE1
607080	46,XY gonadal dysgenesis - motor and sensory neuropathy	DHH
219050	Cryptorchidism, Unilateral Or Bilateral	INSL3
615774	Oocyte Maturation Defect 1	ZP1
616780	Oocyte Maturation Defect 2	TUBB8
614674	Menstrual Cycle-Dependent Periodic Fever	HTR1A
616814	Preimplantation Embryonic Lethality 1	TLE6
606170	Genitopatellar syndrome	KAT6B
175100	Gardner syndrome	APC
608456	Familial adenomatous polyposis 2	MUTYH
276300	Turcot Syndrome	MLH1, MSH2, MSH6, PMS2, APC
120435	Hereditary Nonpolyposis Colorectal cancer type 1	MSH2
609310	Hereditary Nonpolyposis Colorectal cancer type 2	MLH1
614337	Hereditary Nonpolyposis Colorectal cancer type 4	PMS2
614350	Hereditary Nonpolyposis Colorectal cancer type 5	MSH6
614331	Hereditary Nonpolyposis Colorectal cancer type 6	TGFBR2
614385	Hereditary Nonpolyposis Colorectal cancer type 7	MLH3
613244	Hereditary Nonpolyposis Colorectal cancer type 8	EPCAM
175200	Peutz-Jeghers syndrome	STK11
610069	Hereditary Mixed Polyposis Syndrome 2	BMPR1A
174900	Juvenile polyposis syndrome	SMAD4, BMPR1A
613870	Hirschsprung disease, cardiac defects, and autonomic dysfunction	ECE1
211600	Progressive Familial Intrahepatic Cholestasis 1	ATP8B1
601847	Progressive Familial Intrahepatic Cholestasis 2	ABCB11
602347	Progressive Familial Intrahepatic Cholestasis 3	ABCB4
615878	Progressive Familial Intrahepatic Cholestasis 4	TJP2
243300	Benign Recurrent Intrahepatic Cholestasis 1	ATP8B1
605479	Benign Recurrent Intrahepatic Cholestasis 2	ABCB11
147480	Intrahepatic Cholestasis Of Pregnancy, 1	ATP8B1
614972	Intrahepatic Cholestasis Of Pregnancy, 3	ABCB4
167800	Pancreatitis, hereditary	PRSS1, SPINK1
222700	Lysinuric Protein Intolerance	SLC7A7
246700	Chylomicron Retention Disease	SAR1B
223000	Lactose Intolerance	LCT
223100	Lactose Intolerance, Adult Type	MCM6
606824	Glucose-galactose Malabsorption	SLC5A1
607748	Familial Hypercholanemia	EPHX1, BAAT, TJP2
608594	Congenital Generalized Lipodystrophy Type 1	AGPAT2
269700	Congenital Generalized Lipodystrophy Type 2	BSCL2
612526	Congenital Generalized Lipodystrophy Type 3	CAV1
613327	Congenital Generalized Lipodystrophy Type 4	PTRF
607765	Congenital Bile Acid Synthesis Defect 1	HSD3B7
235555	Congenital Bile Acid Synthesis Defect 2	AKR1D1
613812	Congenital Bile Acid Synthesis Defect 3	CYP7B1
214950	Congenital Bile Acid Synthesis Defect 4	AMACR
616278	Congenital Bile Acid Synthesis Defect 5	ABCD3
300048	Chronic Idiopathic Neuronal Intestinal Pseudoobstruction	FLNA
214700	Familial Chloride Diarrhea	SLC26A3
251850	Diarrhea with Microvillus Atrophy 2	MYO5B
270420	Congenital Sodium Diarrhea	SPINT2
610370	Congenital Malabsorptive Diarrhea 4	NEUROG3
613217	Diarrhea 5 With Congenital Tufting Enteropathy	EPCAM
614616	Diarrhea 6	GUCY2C
615863	Diarrhea 7	DGAT1
616868	Diarrhea 8, Secretory Sodium, Congenital	SLC9A3
226200	Enterokinase Deficiency	TMPRSS15
600803	Gallbladder Disease 1	ABCB4
611465	Gallbladder Disease 4	ABCG8
602014	Hypomagnesemia 1, intestinal	TRPM6
615438	Infantile Liver Failure Syndrome 1	LARS
615486	Interstitial lung and liver disease	MARS
613070	Infantile Transient Liver Failure	TRMU
612567	Inflammatory Bowel Disease 25	IL10RB
613148	Inflammatory Bowel Disease 28	IL10RA
604901	North American Indian Childhood Cirrhosis	CIRH1A
174050	Polycystic Liver Disease 1	PRKCSH
617004	Polycystic Liver Disease 2	SEC63
237450	Rotor Syndrome	SLCO1B3, SLCO1B1
222470	Trichohepatoenteric syndrome 1	TTC37
614602	Trichohepatoenteric Syndrome 2	SKIV2L
613291	Bile Acid Malabsorption, Primary	SLC10A2
215600	Familial Cirrhosis	KRT18, KRT8
615237	Congenital Short Bowel Syndrome	CLMP
243150	Multiple Intestinal Atresia	TTC7A
614665	Meconium Ileus	GUCY2C
608189	Tropical Calcific Pancreatitis	SPINK1
617068	Portal hypertension, noncirrhotic	DGUOK
617394	Neonatal Sclerosing cholangitis	DCDC2
105650	Diamond-Blackfan Anemia 1	RPS19
610629	Diamond-Blackfan Anemia 3	RPS24
612528	Diamond-Blackfan Anemia 5	RPL35A
612561	Diamond-Blackfan Anemia 6	RPL5
612562	Diamond-Blackfan Anemia 7	RPL11
613308	Diamond-Blackfan Anemia 9	RPS10
613309	Diamond-Blackfan Anemia 10	RPS26
614900	Diamond-Blackfan Anemia 11	RPL26
615550	Diamond-Blackfan Anemia 12	RPL15
615909	Diamond-Blackfan Anemia 13	RPS29
300946	Diamond-Blackfan anemia 14 with mandibulofacial dysostosis	TSR2
606164	Diamond-Blackfan anemia 15 with mandibulofacial dysostosis	RPS28
300835	X-linked Anemia with/without neutropenia and/or platelet abnormalities	GATA1
227650	Fanconi anemia, complementation group A	FANCA
300514	Fanconi anemia, complementation group B	FANCB
227645	Fanconi anemia, complementation group C	FANCC
605724	Fanconi anemia, complementation group D1	BRCA2
227646	Fanconi anemia, complementation group D2	FANCD2
600901	Fanconi anemia, complementation group E	FANCE
603467	Fanconi anemia, complementation group F	FANCF
614082	Fanconi anemia, complementation group G	FANCG
609053	Fanconi anemia, complementation group I	FANCI
609054	Fanconi anemia, complementation group J	BRIP1
614083	Fanconi anemia, complementation group L	FANCL
610832	Fanconi anemia, complementation group N	PALB2
613390	Fanconi anemia, complementation group O	RAD51C
613951	Fanconi anemia, complementation group P	SLX4
615272	Fanconi anemia, complementation group Q	ERCC4
616435	Fanconi anemia, complementation group T	UBE2T
224120	Congenital Dyserythropoietic Anemia Type Ia	CDAN1
615631	Congenital Dyserythropoietic Anemia Type Ib	C15orf41
224100	Congenital Dyserythropoietic Anemia Type II	SEC23B
613673	Congenital Dyserythropoietic Anemia Type IV	KLF1
306700	Hemophilia A	F8
306900	Hemophilia B	F9
193400	Von Willebrand Disease, Type 1	VWF
613554	Von Willebrand Disease, Type 2	VWF
277480	Von Willebrand Disease, Type 3	VWF
613679	Congenital Prothrombin Deficiency	F2
202400	Congenital Afibrinogenemia	FGA, FGB, FGG
313900	Thrombocytopenia 1	WAS
188000	Thrombocytopenia 2	ANKRD26, MASTL
612004	Thrombocytopenia 4	CYCS
616216	Thrombocytopenia 5	ETV6
616937	Thrombocytopenia 6	SRC
604498	Congenital Amegakaryocytic Thrombocytopenia	MPL
314050	Thrombocytopenia with beta-thalassemia	GATA1
305371	GATA1-Related X-Linked Cytopenia	GATA1
605249	Sebastian Syndrome	MYH9
227400	Factor V deficiency	F5
301040	Alpha-Thalassemia X-Linked Intellectual Disability Syndrome	ATRX
604131	Alpha-Thalassemia	HBA2, HBA1
613985	Beta-Thalassemia	HBB
603903	Sickle Cell Disease	HBB
188055	Factor V Leiden Thrombophilia	F5
200100	Abetalipoproteinemia	MTTP
176860	Autosomal dominant Thrombophilia due to protein C deficiency	PROC
612304	Autosomal recessive Thrombophilia due to protein C deficiency	PROC
612336	Autosomal dominant Thrombophilia due to protein S deficiency	PROS1
614514	Autosomal recessive Thrombophilia due to protein S deficiency	PROS1
182900	Spherocytosis 1	ANK1
616649	Spherocytosis 2	SPTB
270970	Spherocytosis 3	SPTA1
612653	Spherocytosis 4	SLC4A1
612690	Spherocytosis 5	EPB42
153670	Bernard-Soulier syndrome, type A2	GP1BA
231200	Bernard-Soulier syndrome	GP1BA, GP9, GP1BB
273800	Glanzmann thrombasthenia	ITGB3, ITGA2B
177820	Pseudo-von Willebrand Disease	GP1BA
139090	Gray Platelet Syndrome	NBEAL2
601709	Quebec Platelet Disorder	PLAU
155100	May-Hegglin Anomaly	MYH9
262890	Scott syndrome	ANO6
609821	Bleeding Disorder Platelet Type 8	P2RY12
608404	Platelet Glycoprotein IV Deficiency	CD36
614201	Bleeding Disorder Platelet Type 11	GP6
614158	Bleeding Disorder Platelet Type 14	TBXAS1
615193	Bleeding Disorder Platelet Type 15	ACTN1
187800	Bleeding Disorder Platelet Type 16	ITGB3, ITGA2B
187900	Bleeding Disorder Platelet Type 17	GFI1B
615888	Bleeding Disorder Platelet Type 18	RASGRP2
616176	Bleeding Disorder Platelet Type 19	PRKACG
616913	Bleeding Disorder Platelet Type 20	SLFN14
617443	Bleeding Disorder Platelet Type 21	FLI1
209300	Atransferrinemia	TF
194380	Dehydrated Hereditary Stomatocytosis 1	PIEZO1
616689	Dehydrated Hereditary Stomatocytosis 2	KCNN4
611804	Elliptocytosis 1	EPB41
130600	Elliptocytosis 2	SPTA1
182870	Elliptocytosis 3	SPTB
166900	Elliptocytosis 4	SLC4A1
227300	Combined Deficiency of Factor V and Factor VIII type 1	LMAN1
613625	Combined Deficiency of Factor V and Factor VIII type 2	MCFD2
227500	Factor VII Deficiency	F7
227600	Factor X Deficiency	F10
612416	Factor XI Deficiency	F11
234000	Factor XII Deficiency	F12
613225	Factor XIII Subunit A Deficiency	F13A1
613235	Factor XIII Subunit B Deficiency	F13B
133100	Familial Erythrocytosis 1	EPOR
263400	Familial Erythrocytosis 2	VHL
609820	Familial Erythrocytosis 3	EGLN1
611783	Familial Erythrocytosis 4	EPAS1
612631	Hemolytic Anemia due to Adenylate Kinase Deficiency	AK1
230450	Hemolytic Anemia due to Gamma-glutamylcysteine Synthetase Deficiency	GCLC
614164	Hemolytic Anemia due to Glutathione Peroxidase Deficiency	GPX1
235700	Hemolytic Anemia due to Hexokinase Deficiency	HK1
266120	Hemolytic Anemia due to UMPH1 Deficiency	NT5C3A
613470	Nonspherocytic Hemolytic Anemia due to Glucose Phosphate Isomerase Deficiency	GPI
266140	Hereditary Pyropoikilocytosis	SPTA1
300751	Sideroblastic Anemia 1	ALAS2
182170	Sideroblastic Anemia 4	HSPA9
205950	Pyridoxine-Refractory Sideroblastic Anemia 2	SLC25A38
616860	Pyridoxine-Refractory Sideroblastic Anemia 3	GLRX5
206100	Hypochromic Microcytic Anemia with Iron Overload 1	SLC11A2
615234	Hypochromic Microcytic Anemia with Iron Overload 2	STEAP3
206200	Iron-Refractory Iron Deficiency Anemia	TMPRSS6
261100	Megaloblastic anemia-1	CUBN, AMN
613839	Megaloblastic Anemia due to Dihydrofolate Reductase Deficiency	DHFR
249270	Thiamine-Responsive Megaloblastic Anemia Syndrome	SLC19A2
250800	Methemoglobinemia Due to Deficiency of Methemoglobin Reductase	CYB5R3
250790	Methemoglobinemia Type IV	CYB5A
162830	Hereditary Neutrophilia	CSF3R
613329	Plasminogen Activator Inhibitor-1 Deficiency	SERPINE1
268150	Rh-null, regulator type	RHAG
277450	Vitamin K-Dependent Clotting Factors, Combined Deficiency of, 1	GGCX
607473	Vitamin K-Dependent Clotting Factors, Combined Deficiency of, 2	VKORC1
614081	Anhaptoglobinemia	HP
614675	Bone marrow failure syndrome 1	SRP72
615715	Bone marrow failure syndrome 2	ERCC6L2
187950	Thrombocythemia 1	THPO
154800	Mast Cell Disease	KIT
102900	Pyruvate Kinase Hyperactivity	PKLR
262850	Alpha-2-Plasmin Inhibitor Deficiency	SERPINF2
616000	Analbuminemia	ALB
271400	Isolated Congenital Asplenia	RPSA
603902	Beta-Thalassemia, Dominant Inclusion Body Type	HBB
616004	Congenital Dysfibrinogenemia	FGA, FGB, FGG, FGA
231900	Hemolytic Anemia Due To Glutathione Synthetase Deficiency Of Erythrocytes	GSS
140700	Heinz Body Anemias	HBA2, HBB, HBA1
614034	Heme Oxygenase 1 Deficiency	HMOX1
613978	Hemoglobin H Disease	HBA1, HBA2
612300	Cd59-Mediated Hemolytic Anemia With Or Without Immune-Mediated Polyneuropathy	CD59
612356	Heparin Cofactor II Deficiency	SERPIND1
228960	High Molecular Weight Kininogen Deficiency	KNG1
613112	Macrothrombocytopenia, Autosomal Dominant, Tubb1-Related	TUBB1
131440	Chronic Myeloproliferative Disorder With Eosinophilia	PDGFRB
614278	Platelet-Activating Factor Acetylhydrolase Deficiency	PLA2G7
612423	Prekallikrein Deficiency	KLKB1
269600	Sea-Blue Histiocyte Disease	APOE
616084	Sideroblastic Anemia With B-Cell Immunodeficiency, Periodic Fevers,And Developmental Delay	TRNT1
185070	Stormorken Syndrome	STIM1
300367	Thrombocytopenia, X-Linked, With Or Without Dyserythropoietic Anemia	GATA1
613116	Thrombophilia Due To Histidine-Rich Glycoprotein Deficiency	HRG
614486	Thrombophilia Due To Thrombomodulin Defect	THBD
300807	Thrombophilia X-Linked, Due To Factor IX Defect	F9
185000	Overhydrated hereditary stomatocytosis	RHAG
185020	Cryohydrocytosis	SLC4A1
301310	X-linked sideroblastic anemia and ataxia	ABCB7
610168	Loeys-Dietz syndrome type 2	TGFBR2
613795	Loeys-Dietz syndrome type 3	SMAD3
614816	Loeys-Dietz syndrome type 4	TGFB2
615582	Loeys-Dietz syndrome type 5	TGFB3
132900	Familial Aortic aneurysm and thoracic 4	MYH11
611788	Familial Aortic aneurysm and thoracic 6	ACTA2
613780	Familial Aortic aneurysm and thoracic 7	MYLK
615436	Familial Aortic aneurysm and thoracic 8	PRKG1
616166	Familial Aortic aneurysm and thoracic 9	MFAP5
208050	Arterial tortuosity syndrome	SLC2A10
154700	Marfan Syndrome	FBN1
612199	Cerebroretinal Microangiopathy With Calcifications And Cysts	CTC1
187300	Hereditary Hemorrhagic Telangiectasia type 1	ENG
600376	Hereditary Hemorrhagic Telangiectasia type 2	ACVRL1
615506	Hereditary Hemorrhagic Telangiectasia type 5	GDF2
175050	SMAD4-Related Hereditary Hemorrhagic Telangiectasia	SMAD4
608354	Capillary malformation-arteriovenous malformation syndrome	RASA1
105150	CST3-Related Cerebral Amyloid Angiopathy	CST3
605714	APP-Related Cerebral Amyloid Angiopathy	APP
176500	ITM2B-Related Cerebral Amyloid Angiopathy 1	ITM2B
117300	ITM2B-Related Cerebral Amyloid Angiopathy 2	ITM2B
607595	Brain Small-Vessel Disease with Hemorrhage	COL4A1
600195	Multiple Cutaneous and Mucosal Venous Malformations	TEK
142900	Holt-Oram Syndrome	TBX5
153400	Lymphedema-Distichiasis Syndrome	FOXC2
265380	Alveolar Capillary Dysplasia With Misalignment Of Pulmonary Veins	FOXF1
177200	Liddle Syndrome	SCNN1B, SCNN1G
185500	Supravalvular Aortic Stenosis	ELN
194200	Wolff-Parkinson-White syndrome	PRKAG2
601144	Brugada syndrome 1	SCN5A
611777	Brugada syndrome 2	GPD1L
611875	Brugada syndrome 3	CACNA1C
611876	Brugada syndrome 4	CACNB2
613119	Brugada syndrome 6	KCNE3
613120	Familial Atrial Fibrillation 16	SCN3B
613123	Brugada syndrome 8	HCN4
616399	Brugada syndrome 9	KCND3
264800	Pseudoxanthoma Elasticum	ABCC6
115200	Dilated Cardiomyopathy 1A	LMNA
612158	Dilated Cardiomyopathy 1AA	ACTN2
612877	Dilated Cardiomyopathy 1BB	DSG2
613122	Dilated Cardiomyopathy 1CC	NEXN
601494	Dilated Cardiomyopathy 1D	TNNT2
613172	Dilated Cardiomyopathy 1DD	RBM20
601154	Dilated Cardiomyopathy 1E	SCN5A
613252	Dilated Cardiomyopathy 1EE	MYH6
613286	Dilated Cardiomyopathy 1FF	TNNI3
604145	Dilated Cardiomyopathy 1G	TTN
613642	Dilated Cardiomyopathy 1GG	SDHA
613881	Dilated Cardiomyopathy 1HH	BAG3
604765	Dilated Cardiomyopathy 1I	DES
615184	Dilated Cardiomyopathy 1II	CRYAB
605362	Dilated Cardiomyopathy 1J	EYA4
615235	Dilated Cardiomyopathy 1JJ	LAMA4
615248	Dilated Cardiomyopathy 1KK	MYPN
606685	Dilated Cardiomyopathy 1L	SGCD
615373	Dilated Cardiomyopathy 1LL	PRDM16
607482	Dilated Cardiomyopathy 1M	CSRP3
615396	Dilated Cardiomyopathy 1MM	MYBPC3
607487	Dilated Cardiomyopathy 1N	TCAP
615916	Dilated Cardiomyopathy 1NN	RAF1
608569	Dilated Cardiomyopathy 1O	ABCC9
609909	Dilated Cardiomyopathy 1P	PLN
613424	Dilated Cardiomyopathy 1R	ACTC1
613426	Dilated Cardiomyopathy 1S	MYH7
613694	Dilated Cardiomyopathy 1U	PSEN1
613697	Dilated Cardiomyopathy 1V	PSEN2
611407	Dilated Cardiomyopathy 1W	VCL
611615	Dilated Cardiomyopathy 1X	FKTN
611878	Dilated Cardiomyopathy 1Y	TPM1
611879	Dilated Cardiomyopathy 1Z	TNNC1
611880	Dilated Cardiomyopathy 2A	TNNI3
302045	Dilated Cardiomyopathy 3B	DMD
601493	Left ventricular noncompaction 3 with or without dilated cardiomyopathy	LDB3
616117	Cardiac Conduction Disease With Or Without Dilated Cardiomyopathy	TNNI3K
212112	Dilated Cardiomyopathy With Hypergonadotropic Hypogonadism	LMNA
605676	Dilated Cardiomyopathy With Woolly Hair And Keratoderma	DSP
615821	Dilated Cardiomyopathy With Woolly Hair, Keratoderma, And Toothagenesis	DSP
192600	Familial Hypertrophic Cardiomyopathy 1	MYH7
115195	Familial Hypertrophic Cardiomyopathy 2	TNNT2
115196	Familial Hypertrophic Cardiomyopathy 3	TPM1
115197	Familial Hypertrophic Cardiomyopathy 4	MYBPC3
600858	Familial Hypertrophic Cardiomyopathy 6	PRKAG2
613690	Familial Hypertrophic Cardiomyopathy 7	TNNI3
608751	Familial Hypertrophic Cardiomyopathy 8	MYL3
613765	Familial Hypertrophic Cardiomyopathy 9	TTN
608758	Familial Hypertrophic Cardiomyopathy 10	MYL2
612098	Familial Hypertrophic Cardiomyopathy 11	ACTC1
612124	Familial Hypertrophic Cardiomyopathy 12	CSRP3
613243	Familial Hypertrophic Cardiomyopathy 13	TNNC1
613251	Familial Hypertrophic Cardiomyopathy 14	MYH6
613255	Familial Hypertrophic Cardiomyopathy 15	VCL
613838	Familial Hypertrophic Cardiomyopathy 16	MYOZ2
613873	Familial Hypertrophic Cardiomyopathy 17	JPH2
613874	Familial Hypertrophic Cardiomyopathy 18	PLN
613875	Familial Hypertrophic Cardiomyopathy 19	CALR3
613876	Familial Hypertrophic Cardiomyopathy 20	NEXN
115210	Familial Restrictive Cardiomyopathy 1	TNNI3
612422	Familial Restrictive Cardiomyopathy 3	TNNT2
617047	Familial Restrictive Cardiomyopathy 5	FLNC
607554	Familial Atrial Fibrillation 3	KCNQ1
611493	Familial Atrial Fibrillation 4	KCNE2
612201	Familial Atrial Fibrillation 6	NPPA
612240	Familial Atrial Fibrillation 7	KCNA5
613980	Familial Atrial Fibrillation 9	KCNJ2
614022	Familial Atrial Fibrillation 10	SCN5A
614049	Familial Atrial Fibrillation 11	GJA5
614050	Familial Atrial Fibrillation 12	ABCC9
615378	Familial Atrial Fibrillation 14	SCN2B
615770	Familial Atrial Fibrillation 15	NUP155
611819	Familial Atrial Fibrillation 17	SCN4B
107970	Arrhythmogenic Right Ventricular Dysplasia/Cardiomyopathy 1	TGFB3
600996	Arrhythmogenic Right Ventricular Dysplasia/Cardiomyopathy 2	RYR2
604400	Arrhythmogenic Right Ventricular Dysplasia/Cardiomyopathy 5	TMEM43
607450	Arrhythmogenic Right Ventricular Dysplasia/Cardiomyopathy 8	DSP
609040	Arrhythmogenic Right Ventricular Dysplasia/Cardiomyopathy 9	PKP2
610193	Arrhythmogenic Right Ventricular Dysplasia/Cardiomyopathy 10	DSG2
610476	Arrhythmogenic Right Ventricular Dysplasia/Cardiomyopathy 11	DSC2
611528	Arrhythmogenic Right Ventricular Dysplasia/Cardiomyopathy 12	JUP
615616	Arrhythmogenic Right Ventricular Dysplasia/Cardiomyopathy 13	CTNNA3
208000	Generalized Arterial Calcification of Infancy 1	ENPP1
614473	Generalized Arterial Calcification of Infancy 2	ABCC6
607941	Atrial Septal Defect 2	GATA4
614089	Atrial Septal Defect 3	MYH6
611363	Atrial Septal Defect 4	TBX20
612794	Atrial Septal Defect 5	ACTC1
613087	Atrial Septal Defect 6	TLL1
614433	Atrial Septal Defect 8	CITED2
606217	Atrioventricular septal defect, partial, with heterotaxy syndrome	CRELD1
600309	Atrioventricular Septal Defect 3	GJA1
614430	Atrioventricular Septal Defect 4	GATA4
604772	Catecholaminergic Polymorphic Ventricular Tachycardia 1	RYR2
611938	Catecholaminergic Polymorphic Ventricular Tachycardia 2	CASQ2
614916	Catecholaminergic Polymorphic Ventricular Tachycardia 4	CALM1
615441	Catecholaminergic Polymorphic Ventricular Tachycardia 5, with or without muscle weakness	TRDN
217095	Conotruncal Heart Malformations	NKX2-6, NKX2-5, GATA6, ZFPM2, GDF1, TBX1
314400	X-linked Cardiac Valvular Dysplasia	FLNA
235510	Hennekam Lymphangiectasia-Lymphedema Syndrome	CCBE1
106100	Hereditary Angioedema Type I and Type II	SERPING1
610618	Hereditary Angioedema Type III	F12
153100	Hereditary Lymphedema IA	FLT4
616843	Hereditary Lymphedema III	PIEZO1
241550	Hypoplastic Left Heart Syndrome 1	GJA1
614435	Hypoplastic Left Heart Syndrome 2	NKX2-5
604169	Left Ventricular Noncompaction 1	DTNA
615092	Left Ventricular Noncompaction 7	MIB1
109730	Aortic Valve Disease 1	NOTCH1
614823	Aortic Valve Disease 2	SMAD6
603830	Long QT Syndrome 3	SCN5A
613695	Long QT Syndrome 5	KCNE1
613693	Long QT Syndrome 6	KCNE2
170390	Long QT Syndrome 7	KCNJ2
601005	Long QT Syndrome 8	CACNA1C
611818	Long QT Syndrome 9	CAV3
611820	Long QT Syndrome 11	AKAP9
613485	Long QT Syndrome 13	KCNJ5
616247	Long QT Syndrome 14	CALM1
616249	Long QT Syndrome 15	CALM2
609620	Short QT Syndrome 1	KCNH2
609621	Short QT Syndrome 2	KCNQ1
609622	Short QT Syndrome 3	KCNJ2
614042	Moyamoya disease 5	ACTA2
615750	Moyamoya disease 6 with achalasia	GUCY1A3
236600	Nonsyndromic Hydrocephalus 1	CCDC88C
615219	Nonsyndromic Hydrocephalus 2	MPDZ
307000	X-linked hydrocephalus	L1CAM
113900	Progressive Familial Heart Block, Type IA	SCN5A
604559	Progressive Familial Heart Block, Type IB	TRPM4
265450	Pulmonary Venoocclusive Disease 1	BMPR2
234810	Pulmonary Venoocclusive Disease 2	EIF2AK4
608567	Sick Sinus Syndrome 1	SCN5A
163800	Sick Sinus Syndrome 2	HCN4
614429	Ventricular Septal Defect 1	GATA4
614431	Ventricular Septal Defect 2	CITED2
614432	Ventricular Septal Defect 3	NKX2-5
610878	Vesicoureteral Reflux 2	ROBO2
613674	Vesicoureteral Reflux 3	SOX17
608320	Coronary Artery Disease 1	MEF2A
610947	Coronary Artery Disease 2	LRP6
617035	Patent ductus arteriosus 2	TFAP2B
608808	Dextro-looped Transposition of the Great Arteries 1	MED13L
108010	Arteriovenous Malformations Of The Brain	IL6
108770	Atrial Standstill 1	GJA5
615745	Atrial Standstill 2	NPPA
609129	Autosomal Dominant Auditory Neuropathy 1	DIAPH3
600919	Ankyrin-B-Related Cardiac Arrhythmia	ANK2
115080	Cardiac Conduction Defect	AKAP10
616006	Hennekam Lymphangiectasia-Lymphedema Syndrome 2	FAT4
608622	Resistance To Diastolic Hypertension	KCNMB1
605115	Hypertension, Early-Onset, Autosomal Dominant, With Severe Exacerbation in Pregnancy	NR3C2
615907	Hereditary Lymphedema ID	VEGFC
615688	Childhood-Onset Polyarteritis Nodosa	CECR1
614595	Preeclampsia/Eclampsia 5	CORIN
177850	Forme Fruste Pseudoxanthoma Elasticum	ABCC6
610842	Pseudoxanthoma Elasticum-Like Disorder With Multiple Coagulation Factor deficiency	GGCX
614896	Sinoatrial Node Dysfunction And Deafness	CACNA1D
182410	Sneddon Syndrome	CECR1
192605	Familial Ventricular Tachycardia	GNAI2
607829	Mitral Valve Prolapse 2	DCHS1
NA039	ANKRD1-Related Dilated Cardiomyopathy	ANKRD1
NA040	Childhood Restrictive Cardiomyopathy	ACTA1
NA047	Familial Isolated Noncompaction of Left Ventricular Myocardium	TAZ
133780	Familial Exudative Vitreoretinopathy 1	FZD4
305390	Familial Exudative Vitreoretinopathy 2	NDP
601813	Familial Exudative Vitreoretinopathy 4	LRP5
613310	Familial Exudative Vitreoretinopathy 5	TSPAN12
616468	Familial Exudative Vitreoretinopathy 6	ZNF408
617572	Familial Exudative Vitreoretinopathy 7	CTNNB1
610532	Hypomyelination and Congenital Cataract	FAM126A
610092	Microphthalmia with coloboma 3	VSX2
611638	Microphthalmia with coloboma 5	SHH
613703	Microphthalmia with coloboma 6	PRSS56
614497	Microphthalmia with coloboma 7	ABCB6
601186	Microphthalmia with coloboma 8	STRA6
615145	Microphthalmia with coloboma 9	TENM3
616428	Microphthalmia with coloboma 10	RBP4
309800	Syndromic Microphthalmia 1	NAA10
300166	Syndromic Microphthalmia 2	BCOR
206900	Syndromic Microphthalmia 3	SOX2
610125	Syndromic Microphthalmia 5	OTX2
607932	Syndromic Microphthalmia 6	BMP4
309801	Syndromic Microphthalmia 7	HCCS
615524	Syndromic Microphthalmia 12	RARB
300915	Syndromic Microphthalmia 13	HMGB3
615877	Syndromic Microphthalmia 14	MAB21L2
610093	Isolated Microphthalmia 2	VSX2
611038	Isolated Microphthalmia 3	RAX
613094	Isolated Microphthalmia 4	GDF6
611040	Isolated Microphthalmia 5	MFRP
613517	Isolated Microphthalmia 6	PRSS56
613704	Isolated Microphthalmia 7	GDF3
615113	Isolated Microphthalmia 8	ALDH1A3
120330	Renal Coloboma Syndrome	PAX2
206920	Waardenburg anophthalmia syndrome	SMOC1
616335	Autosomal Recessive Microcephaly And Chorioretinopathy 3	TUBGCP4
110100	Blepharophimosis Syndrome	FOXL2
143200	VCAN-Related Vitreoretinopathy	VCAN
303100	Choroideremia	CHM
604356	Duane retraction syndrome 2	CHN1
617041	Duane retraction syndrome 3 with or without deafness	MAFB
180500	Axenfeld-Rieger syndrome, type 1	PITX2
602482	Axenfeld-Rieger Syndrome, Type 3	FOXC1
267750	Knobloch Syndrome Type I	COL18A1
216900	Achromatopsia 2	CNGA3
262300	Achromatopsia 3	CNGB3
613856	Achromatopsia 4	GNAT2
616517	Achromatopsia 7	ATF6
180100	Retinitis pigmentosa 1	RP1
312600	Retinitis pigmentosa 2	RP2
300029	Retinitis pigmentosa 3	RPGR
613731	Retinitis pigmentosa 4	RHO
608133	Retinitis pigmentosa 7	PRPH2, ROM1
180105	Retinitis pigmentosa 10	IMPDH1
600138	Retinitis pigmentosa 11	PRPF31
600105	Retinitis pigmentosa 12	CRB1
600059	Retinitis pigmentosa 13	PRPF8
600132	Retinitis pigmentosa 14	TULP1
600852	Retinitis pigmentosa 17	CA4
601414	Retinitis pigmentosa 18	PRPF3
601718	Retinitis pigmentosa 19	ABCA4
613794	Retinitis pigmentosa 20	RPE65
300424	Retinitis pigmentosa 23	OFD1
602772	Retinitis pigmentosa 25	EYS
608380	Retinitis pigmentosa 26	CERKL
613750	Retinitis pigmentosa 27	NRL
606068	Retinitis pigmentosa 28	FAM161A
607921	Retinitis pigmentosa 30	FSCN2
609923	Retinitis pigmentosa 31	TOPORS
610359	Retinitis pigmentosa 33	SNRNP200
610282	Retinitis pigmentosa 35	SEMA4A
610599	Retinitis pigmentosa 36	PRCD
611131	Retinitis pigmentosa 37	NR2E3
613862	Retinitis pigmentosa 38	MERTK
613809	Retinitis pigmentosa 39	USH2A
613801	Retinitis pigmentosa 40	PDE6B
612095	Retinitis pigmentosa 41	PROM1
612943	Retinitis pigmentosa 42	KLHL7
613810	Retinitis pigmentosa 43	PDE6A
613769	Retinitis pigmentosa 44	RGR
613767	Retinitis pigmentosa 45	CNGB1
612572	Retinitis pigmentosa 46	IDH3B
613758	Retinitis pigmentosa 47	SAG
613827	Retinitis pigmentosa 48	GUCA1B
613756	Retinitis pigmentosa 49	CNGA1
613194	Retinitis pigmentosa 50	BEST1
613464	Retinitis pigmentosa 51	TTC8
613428	Retinitis pigmentosa 54	C2orf71
613575	Retinitis pigmentosa 55	ARL6
613581	Retinitis pigmentosa 56	IMPG2
613582	Retinitis pigmentosa 57	PDE6G
613617	Retinitis pigmentosa 58	ZNF513
613861	Retinitis pigmentosa 59	DHDDS
613983	Retinitis pigmentosa 60	PRPF6
614180	Retinitis pigmentosa 61	CLRN1
614181	Retinitis pigmentosa 62	MAK
614500	Cone-Rod Dystrophy 16	C8orf37
615233	Retinitis pigmentosa 66	RBP3
615565	Retinitis pigmentosa 67	NEK2
615725	Retinitis pigmentosa 68	SLC7A14
615780	Retinitis pigmentosa 69	KIZ
615922	Retinitis pigmentosa 70	PRPF4
616394	Retinitis pigmentosa 71	IFT172
616469	Retinitis pigmentosa 72	ZNF408
616562	Retinitis pigmentosa 74	BBS2
617023	Retinitis pigmentosa 75	AGBL5
617123	Retinitis pigmentosa 76	POMGNT1
617460	Retinitis pigmentosa 79	HK1
616959	Retinitis Pigmentosa And Erythrocytic Microcytosis	TRNT1
310600	Norrie Disease	NDP
615434	Retinitis pigmentosa with or without situs inversus	ARL2BP
120970	Cone-Rod Dystrophy 2	CRX
204000	Leber Congenital Amaurosis 1	GUCY2D
610612	Leber Congenital Amaurosis 12	RD3
204100	Leber Congenital Amaurosis 2	RPE65
604232	Leber Congenital Amaurosis 3	SPATA7
604393	Leber Congenital Amaurosis 4	AIPL1
604537	Leber Congenital Amaurosis 5	LCA5
613826	Leber Congenital Amaurosis 6	RPGRIP1
613829	Leber Congenital Amaurosis 7	CRX
613835	Leber Congenital Amaurosis 8	CRB1
608553	Leber Congenital Amaurosis 9	NMNAT1
611755	Leber Congenital Amaurosis 10	CEP290
613837	Leber Congenital Amaurosis 11	IMPDH1
612712	Leber Congenital Amaurosis 13	RDH12
613341	Leber congenital amaurosis 14	LRAT
613843	Leber congenital amaurosis 15	TULP1
614186	Leber congenital amaurosis 16	KCNJ13
615360	Leber congenital amaurosis 17	GDF6
165500	Optic Atrophy Type 1	OPA1
165300	Optic Atrophy 3	OPA3
612989	Optic Atrophy Type 7	TMEM126A
616289	Optic Atrophy Type 9	ACO2
616732	Optic Atrophy 10 With Or Without Ataxia, Mental Retardation, And Seizures	RTN4IP1
106210	Aniridia 1	PAX6
617141	Aniridia 2	ELP4
312700	X-Linked Juvenile Retinoschisis	RS1
248200	Stargardt Disease 1	ABCA4, CNGB3
600110	Stargardt Disease 3	ELOVL4
603786	Stargardt Disease 4	PROM1
229200	Brittle Cornea Syndrome 1	ZNF469
614170	Brittle Cornea Syndrome 2	PRDM5
610445	Congenital Stationary Night Blindness 1	RHO
310500	Congenital Stationary Night Blindness, Type 1A	NYX
613216	Congenital Stationary Night Blindness, Type 1C	TRPM1
613830	Congenital Stationary Night Blindness, Type 1D	SLC24A1
614565	Congenital Stationary Night Blindness, Type 1E	GPR179
615058	Congenital Stationary Night Blindness, Type 1F	LRIT3
616389	Congenital Stationary Night Blindness, Type 1G	GNAT1
617024	Congenital Stationary Night Blindness, Type 1H	GNB3
163500	Congenital Stationary Night Blindness,Type 2	PDE6B
300071	Congenital Stationary Night Blindness, Type 2A	CACNA1F
610427	Congenital Stationary Night Blindness, Type 2B	CABP4
610444	Congenital Stationary Night Blindness, Type 3	GNAT1
258100	Congenital Stationary Night Blindness Oguchi type 1	SAG
613411	Congenital Stationary Night Blindness Oguchi type 2	GRK1
116200	Cataract 1, multiple types	GJA8
604307	Cataract 2, multiple types	CRYGC
601547	Cataract 3, multiple types	CRYBB2
115700	Cataract 4, multiple types	CRYGD
116800	Cataract 5, multiple types	HSF4
116600	Cataract 6, multiple types	EPHA2
604219	Cataract 9, multiple types	CRYAA
600881	Cataract 10, multiple types	CRYBA1
610623	Cataract 11, multiple types	PITX3
611597	Cataract 12, multiple types	BFSP2
116700	Cataract 13	GCNT2
601885	Cataract 14, multiple types	GJA3
615274	Cataract 15, multiple types	MIP
613763	Cataract 16, multiple types	CRYAB
611544	Cataract 17, multiple types	CRYBB1
610019	Cataract 18	FYCO1
615277	Cataract 19, multiple types	LIM2
116100	Cataract 20, multiple types	CRYGS
609741	Cataract 22	CRYBB3
610425	Cataract 23	CRYBA4
116300	Cataract 30, pulverulent	VIM
605387	Cataract 31, multiple types	CHMP4B
613887	Cataract 36	TDRD7
614691	Cataract 38	AGK
615188	Cataract 39	CRYGB
116400	Cataract 41	WFS1
115900	Cataract 42	CRYBA2
616279	Cataract 43	UNC45B
616509	Cataract 44	LSS
616851	Cataract 45	SIPA1L3
212500	Cataract 46	LEMD2
600886	Hyperferritinemia Cataract Syndrome	FTL
136520	Foveal Hypoplasia and Presenile Cataract Syndrome	PAX6
612540	Compton-North Congenital Myopathy	CNTN1
604116	Cone-Rod Dystrophy 3	ABCA4
613093	Cone-Rod Dystrophy 4	PDE6C
600977	Cone-Rod Dystrophy 5	PITPNM3
601777	Cone-Rod Dystrophy 6	GUCY2D
603649	Cone-Rod Dystrophy 7	RIMS1
612775	Cone-Rod Dystrophy 9	ADAM9
610283	Cone-Rod Dystrophy 10	SEMA4A
610381	Cone-Rod Dystrophy 11	RAX2
612657	Cone-Rod Dystrophy 12	PROM1
608194	Cone-Rod Dystrophy 13	RPGRIP1
602093	Cone-Rod Dystrophy 14	GUCA1A
613660	Cone-Rod Dystrophy 15	CDHR1
615374	Cone-Rod Dystrophy 18	RAB28
615860	Cone-Rod Dystrophy 19	TTLL5
615973	Cone-Rod Dystrophy 20	POC1B
616502	Cone-Rod Dystrophy 21	DRAM2
304020	X-linked Cone-Rod Dystrophy 1	RPGR
300476	X-linked Cone-Rod Dystrophy 3	CACNA1F
613105	Choriodal dystrophy, central areolar 2	PRPH2
608470	Corneal Dystrophy of Bowman Layer, Type 1	TGFBI
121820	Corneal Dystrophy, Epithelial Basement Membrane	TGFBI
204870	Corneal Dystrophy, Gelatinous Drop-Like	TACSTD2
122100	Corneal Dystrophy, Meesmann	KRT3, KRT12
217700	Corneal Endothelial Dystrophy	SLC4A11
217400	Corneal Dystrophy And Perceptive Deafness	SLC4A11
136800	Fuchs Endothelial Corneal Dystrophy 1	COL8A2
613268	Fuchs Endothelial Corneal Dystrophy 4	SLC4A11
613270	Fuchs Endothelial Corneal Dystrophy 6	ZEB1
615523	Fuchs Endothelial Corneal Dystrophy 8	AGBL1
602082	Corneal dystrophy, Thiel-Behnke type	TGFBI
121900	Corneal Dystrophy, Groenouw Type I	TGFBI
609140	Posterior Polymorphous Corneal Dystrophy 2	COL8A2
609141	Posterior Polymorphous Corneal Dystrophy 3	ZEB1
217800	Macular Corneal Dystrophy	CHST6
122200	Lattice Corneal Dystrophy Type I	TGFBI
608471	Lattice Corneal Dystrophy Type IIIA	TGFBI
121850	Corneal Fleck Dystrophy	PIKFYVE
607541	Avellino Corneal Dystrophy	TGFBI
610048	Congenital Stromal Corneal Dystrophy	DCN
137750	Primary Open Angle Glaucoma 1A	MYOC
137760	Primary Open Angle Glaucoma 1E	OPTN
613100	Primary Open Angle Glaucoma 1O	NTF4
609887	Primary Open Angle Glaucoma 1G	WDR36
231300	Primary Open Angle Glaucoma 3A	CYP1B1
613086	Primary Open Angle Glaucoma 3D	LTBP2
603383	Open Angle Glaucoma 1F	ASB10
604229	Peters Anomaly	CYP1B1, PITX2, PAX6
251750	Microspherophakia and/or megalocornea, with ectopia lentis and with or without secondary glaucoma	LTBP2
126600	Doyne Honeycomb Retinal Dystrophy	EFEMP1
225200	Ectopia Lentis et pupillae	ADAMTSL4
129600	Isolated Ectopia Lentis 1	FBN1
225100	Isolated Ectopia Lentis 2	ADAMTSL4
268100	Goldmann-Favre syndrome	NR2E3
107250	Anterior Segment Mesenchymal Dysgenesis	FOXE3, PITX3
601631	Iridogoniodysgenesis, Type 1	FOXC1
137600	Iridogoniodysgenesis, Type 2	PITX2
148300	Keratoconus 1	VSX1
605670	Late-Onset Retinal Degeneration	C1QTNF5
309300	Megalocornea	CHRDL1
607476	Newfoundland Rod-Cone Dystrophy	RLBP1
172870	Pigmented Paravenous Chorioretinal Atrophy	CRB1
136880	Fundus albipunctatus	RHO, PRPH2, RDH5, RLBP1
610024	Retinal Cone Dystrophy 3A	PDE6H
610356	Retinal Cone Dystrophy 3B	KCNV2
610478	Retinal Cone Dystrophy 4	CACNA2D4
180550	Ring Dermoid of Cornea	PITX2
193220	Vitreoretinochoroidopathy	BEST1
210370	Bietti Crystalline Dystrophy	CYP4V2
615722	Bosch-Boonstra-Schaaf Optic Atrophy Syndrome	NR2F1
300834	Macular Degeneration, X-Linked Atrophic	RPGR
616170	Macular Dystrophy With Central Cone Involvement	MFSD8
616151	Macular Dystrophy, Vitelliform, 2	IMPG1
608161	Macular Dystrophy, Vitelliform, 3	PRPH2
153700	Macular Dystrophy, Vitelliform, 4	BEST1
616152	Macular Dystrophy, Vitelliform, 5	IMPG2
613587	Occult Macular Dystrophy	RP1L1
608051	Macular Dystrophy, Retinal, 2	PROM1
607475	Bothnia retinal dystrophy	RLBP1
615458	Microcornea, Myopic Chorioretinal Atrophy, And Telecanthus	ADAMTS18
103100	Adie Pupil	MPZ
300600	Aland Island Eye Disease	CACNA1F
611809	Autosomal Recessive Bestrophinopathy	BEST1
120430	Coloboma Of Optic Nerve	PAX6
120200	Coloboma, Ocular, Autosomal Dominant	PAX6
216820	Coloboma, Ocular, Autosomal Recessive	SALL2
120433	Coloboma, Ocular, With Or Without Hearing Impairment, Cleft Lip/Palate,And/Or Mental Retardation	YAP1
217300	Cornea Plana 2	KERA
615225	Corneal Intraepithelial Dyskeratosis And Ectodermal Dysplasia	NLRP1
269400	Corneal Opacification With Other Ocular Anomalies	PXDN
177650	Exfoliation Syndrome	LOXL1
228980	Benign Familial Fleck Retina	PLA2G5
136900	Pseudoinflammatory Fundus Dystrophy Of Sorsby	TIMP3
148190	Hereditary Keratitis	PAX6
149700	Lacrimal Duct Defect	IGSF3
608908	Myopia 6	SCO2
614167	Autosomal Dominant Myopia 21	ZNF644
615420	Autosomal Dominant Myopia 22	CCDC111
615431	Autosomal Recessive Myopia 23	LRPAP1
615946	Autosomal Dominant Myopia 24	SLC39A5
614292	Myopia, High, With Cataract And Vitreoretinal Degeneration	P3H2
609549	Nanophthalmos 2	MFRP
615972	Nanophthalmos 4	TMEM98
125250	Optic Atrophy With Or Without Deafness, Ophthalmoplegia, Myopathy, Ataxia, and Neuropathy	OPA1
212550	Optic disc anomalies with retinal and/or macular dystrophy	SIX6
165550	Bilateral Optic Nerve Hypoplasia	PAX6
608415	Prolonged electroretinal response suppression	RGS9, RGS9BP
616188	Retinal Dystrophy And Obesity	TUB
616079	Retinal Dystrophy With Inner Retinal Dysfunction And Ganglion Cellabnormalities	ITM2B
615147	Retinal Dystrophy, Iris Coloboma, And Comedogenic Acne Syndrome	RBP4
616108	Retinal Dystrophy, Juvenile Cataracts, And Short Stature Syndrome	RDH11
300455	Retinitis Pigmentosa, X-Linked, And Sinorespiratory Infections, Withor Without Deafness	RPGR
121800	Schnyder Corneal Dystrophy	UBIAD1
108985	Sveinsson Chorioretinal Atrophy	TEAD1
190330	Trichomegaly	FGF5
190900	Tritanopia	OPN1SW
193230	Snowflake Type Vitreoretinal Degeneration	KCNJ13
193235	Neovascular Inflammatory Vitreoretinopathy	CAPN5
180000	Retinal arteries, tortuosity of	COL4A1
611543	Cavitary Optic Disc Anomalies	MMP19
169150	Patterned Macular Dystrophy 1	PRPH2
608970	Patterned Macular Dystrophy 2	CTNNA1
617272	Glaucoma 3, primary congenital, E	TEK
NA028	PAX6-Related Anophthalmia	PAX6
NA033	Type II Collagenopathies	COL2A1
NA041	UNC119-Related late-onset Cone-Rod Dystrophy	UNC119
617315	Anterior segement dysgenesis 6, multiple subtypes	CYP1B1
617547	Retinal dystrophy with or without macular staphyloma	C21orf2
612018	Juvenile cataract with microcornea and glucosuria	SLC16A12
115150	Cardiofaciocutaneous Syndrome 1	BRAF
615278	Cardiofaciocutaneous Syndrome 2	KRAS
615279	Cardiofaciocutaneous Syndrome 3	MAP2K1
615280	Cardiofaciocutaneous Syndrome 4	MAP2K2
300523	Allan-Herndon-Dudley syndrome	SLC16A2
216360	COACH syndrome	TMEM67, CC2D2A, RPGRIP1L
616033	Microcephaly, Short Stature, And Impaired Glucose Metabolism 1	TRMT10A
616817	Microcephaly, Short Stature, And Impaired Glucose Metabolism 2	PPP1R15B
300749	Mental retardation and microcephaly with pontine and cerebellar hypoplasia	CASK
300423	X-Linked Mental Retardation with Epilepsy	ATP6AP2
300643	Rolandic Epilepsy, Mental Retardation, and Speech Dyspraxia	SRPX2
305450	FG Syndrome Type 1	MED12
300321	FG Syndrome Type 2	FLNA
300422	FG Syndrome Type 4	CASK
300895	OHDO syndrome, Maat-Kievit-Brunner type	MED12
248800	Marinesco-Sjogren Syndrome	SIL1
300243	X-Linked Syndromic Mental Retardation, Christianson type	SLC9A6
224050	Cerebellar ataxia, mental retardation, and dysequilibrium syndrome 1	VLDLR
610185	Cerebellar ataxia, mental retardation, and dysequilibrium syndrome 2	WDR81
613227	Cerebellar ataxia, mental retardation, and dysequilibrium syndrome 3	CA8
615268	Cerebellar ataxia, mental retardation, and dysequilibrium syndrome 4	ATP8A2
614113	Autosomal Dominant Mental Retardation 2	DOCK8
612580	Autosomal Dominant Mental Retardation 3	CDH15
612581	Autosomal Dominant Mental Retardation 4	KIRREL3
612621	Autosomal Dominant Mental Retardation 5	SYNGAP1
613970	Autosomal Dominant Mental Retardation 6	GRIN2B
614104	Autosomal Dominant Mental Retardation 7	DYRK1A
614254	Autosomal Dominant Mental Retardation 8	GRIN1
614255	Autosomal Dominant Mental Retardation 9	KIF1A
614256	Autosomal Dominant Mental Retardation 10	CACNG2
614257	Autosomal Dominant Mental Retardation 11	EPB41L1
135900	Autosomal Dominant Mental Retardation 12	ARID1B
614563	Autosomal Dominant Mental Retardation 13	DYNC1H1
614607	Autosomal Dominant Mental Retardation 14	ARID1A
614608	Autosomal Dominant Mental Retardation 15	SMARCB1
614609	Autosomal Dominant Mental Retardation 16	SMARCA4
615009	Autosomal Dominant Mental Retardation 17	PACS1
615074	Autosomal Dominant Mental Retardation 18	GATAD2B
615075	Autosomal Dominant Mental Retardation 19	CTNNB1
615502	Autosomal Dominant Mental Retardation 21	CTCF
615761	Autosomal Dominant Mental Retardation 23	SETD5
615828	Autosomal Dominant Mental Retardation 24	DEAF1
615829	Autosomal Dominant Mental Retardation 25	AHDC1
615834	Autosomal Dominant Mental Retardation 26	AUTS2
615866	Autosomal Dominant Mental Retardation 27	SOX11
615873	Autosomal Dominant Mental Retardation 28	ADNP
616078	Autosomal Dominant Mental Retardation 29	SETBP1
616083	Autosomal Dominant Mental Retardation 30	ZMYND11
616158	Autosomal Dominant Mental Retardation 31	PURA
616268	Autosomal Dominant Mental Retardation 32	KAT6A
616311	Autosomal Dominant Mental Retardation 33	DPP6
616351	Autosomal Dominant Mental Retardation 34	COL4A3BP
616355	Autosomal Dominant Mental Retardation 35	PPP2R5D
616362	Autosomal Dominant Mental Retardation 36	PPP2R1A
616364	Autosomal Dominant Mental Retardation 37	POGZ
616393	Autosomal Dominant Mental Retardation 38	EEF1A2
616579	Autosomal Dominant Mental Retardation 40	CHAMP1
616944	Autosomal Dominant Mental Retardation 41	TBL1XR1
616973	Autosomal Dominant Mental Retardation 42	GNB1
249500	Autosomal Recessive Mental Retardation 1	PRSS12
607417	Autosomal Recessive Mental Retardation 2	CRBN
608443	Autosomal Recessive Mental Retardation 3	CC2D1A
611091	Autosomal Recessive Mental Retardation 5	NSUN2
611092	Autosomal Recessive Mental Retardation 6	GRIK2
611093	Autosomal Recessive Mental Retardation 7	TUSC3
611090	Autosomal Recessive Mental Retardation 12	ST3GAL3
613192	Autosomal Recessive Mental Retardation 13	TRAPPC9
614020	Autosomal Recessive Mental Retardation 14	TECR
614202	Autosomal Recessive Mental Retardation 15	MAN1B1
614249	Autosomal Recessive Mental Retardation 18	MED23
614340	Autosomal Recessive Mental Retardation 27	LINS
614499	Autosomal Recessive Mental Retardation 34	CRADD
615286	Autosomal Recessive Mental Retardation 36	ADAT3
615493	Autosomal Recessive Mental Retardation 37	ANK3
615516	Autosomal Recessive Mental Retardation 38	HERC2
615541	Autosomal Recessive Mental Retardation 39	TTI2
615599	Autosomal Recessive Mental Retardation 40	TAF2
615637	Autosomal Recessive Mental Retardation 41	KPTN
615802	Autosomal Recessive Mental Retardation 42	PGAP1
615817	Autosomal Recessive Mental Retardation 43	KIAA1033
615942	Autosomal Recessive Mental Retardation 44	METTL23
616116	Autosomal Recessive Mental Retardation 46	NDST1
616193	Autosomal Recessive Mental Retardation 47	FMN2
616269	Autosomal Recessive Mental Retardation 48	SLC6A17
616281	Autosomal Recessive Mental Retardation 49	GPT2
616460	Autosomal Recessive Mental Retardation 50	EDC3
616739	Autosomal Recessive Mental Retardation 51	HNMT
616887	Autosomal Recessive Mental Retardation 52	LMAN2L
616917	Autosomal Recessive Mental Retardation 53	PIGG
309530	X-Linked Mental Retardation 1	IQSEC2
309549	X-Linked Mental Retardation 9	FTSJ1
300957	X-Linked Mental Retardation 12	THOC2
300844	X-Linked Mental Retardation 19	RPS6KA3
300143	X-Linked Mental Retardation 21	IL1RAPL1
300558	X-Linked Mental Retardation 30	PAK3
300849	X-Linked Mental Retardation 41	GDI1
300498	X-Linked Mental Retardation 45	ZNF81
300436	X-Linked Mental Retardation 46	ARHGEF6
300210	X-Linked Mental Retardation 58	TSPAN7
300387	X-Linked Mental Retardation 63	ACSL4
300271	X-Linked Mental Retardation 72	RAB39B
300852	X-Linked Mental Retardation 88	AGTR2
300848	X-Linked Mental Retardation 89	ZNF41
300850	X-Linked Mental Retardation 90	DLG3
300577	X-Linked Mental Retardation 91	ZDHHC15
300659	X-Linked Mental Retardation 93	BRWD3
300699	X-Linked Mental Retardation 94	GRIA3
300802	X-Linked Mental Retardation 96	SYP
300803	X-Linked Mental Retardation 97	ZNF711
300912	X-Linked Mental Retardation 98	KIAA2022
300919	X-Linked Mental Retardation 99	USP9X
300923	X-Linked Mental Retardation 100	KIF4A
300928	X-Linked Mental Retardation 101	MID2
300958	X-Linked Mental Retardation 102	DDX3X
304340	X-Linked Syndromic Mental Retardation 5	AP1S2
300438	X-Linked Syndromic Mental Retardation 10	HSD17B10
300238	X-Linked Syndromic Mental Retardation 11	RBMX
300055	X-Linked Syndromic Mental Retardation 13	MECP2
300676	X-Linked Syndromic Mental Retardation 14	UPF3B
300354	X-Linked Syndromic Mental Retardation 15 (Cabezas type)	CUL4B
300886	X-Linked Syndromic Mental Retardation 32	CLIC2
300966	X-Linked Syndromic Mental Retardation 33	TAF1
300967	X-Linked Syndromic Mental Retardation 34	NONO
300998	X-Linked Syndromic Mental Retardation 35	RPL10
300968	X-Linked Syndromic Mental Retardation 99, Female-Restricted	USP9X
300987	X-Linked Syndromic Mental Retardation, Borck type	EIF2S3
616789	Mental Retardation And Distinctive Facial Features With Or Without Cardiac Defects	MED13L
613671	Mental Retardation, Anterior Maxillary Protrusion, And Strabismus	SOBP
610156	Mental Retardation, Truncal Obesity, Retinal Dystrophy, And Micropenis syndrome	INPP5E
309580	X-Linked Mental Retardation-Hypotonic Facies Syndrome 1	ATRX
301900	Borjeson-Forssman-Lehmann syndrome	PHF6
300860	X-Linked Syndromic Mental Retardation, Nascimento-type	UBE2A
300799	X-Linked Syndromic Mental Retardation, Raymond type	ZDHHC9
309583	Snyder-Robinson mental retardation syndrome	SMS
300534	X-Linked Syndromic Mental Retardation, Claes-Jensen type	KDM5C
300706	X-Linked Syndromic Mental Retardation, Turner type	HUWE1
300263	X-linked Mental retardation syndrome, Siderius type	PHF8
300519	X-Linked Syndromic Mental Retardation, Martin-Probst Type	RAB40AL
309548	X-linked Mental retardation, FRAXE type	AFF2
300486	X-Linked Mental Retardation with Cerebellar Hypoplasia and Distinctive Facial Appearance	OPHN1
613670	Mental Retardation with Language Impairment and Autistic Features	FOXP1
300472	Corpus callosum, agenesis of, with mental retardation, ocular coloboma and micrognathia	IGBP1
309500	Renpenning syndrome	PQBP1
612292	Birk-Barel Mental Retardation Dysmorphism Syndrome	KCNK9
206700	Aniridia, Cerebellar Ataxia, And Mental Retardation	PAX6
280000	Coloboma, Congenital Heart Disease, Ichthyosiform Dermatosis, Mental Retardation, and Ear Anomalies Syndrome	PIGL
300434	Stocco dos Santos Mental Retardation Syndrome	SHROOM4
300495	NLGN4X-Related Mental Retardation	NLGN4X
239300	Hyperphosphatasia with Mental Retardation Syndrome 1	PIGV
614749	Hyperphosphatasia with Mental Retardation Syndrome 2	PIGO
614207	Hyperphosphatasia with Mental Retardation Syndrome 3	PGAP2
615716	Hyperphosphatasia with Mental Retardation Syndrome 4	PGAP3
616025	Hyperphosphatasia with Mental Retardation Syndrome 5	PIGW
616809	Hyperphosphatasia with Mental Retardation Syndrome 6	PIGY
300615	Brunner Syndrome	MAOA
212720	Martsolf Syndrome	RAB3GAP2
609313	MEDNIK Syndrome	AP1S1
139210	Myhre Syndrome	SMAD4
601358	Nicolaides-Baraitser Syndrome	SMARCA2
211750	Opitz Trigonocephaly Syndrome	CD96
614325	Pitt-Hopkins-Like Syndrome 2	NRXN1
610954	Pitt-Hopkins syndrome	TCF4
210600	Seckel Syndrome Type 1	ATR
606744	Seckel Syndrome Type 2	RBBP8
613676	Seckel Syndrome Type 4	CENPJ
613823	Seckel Syndrome Type 5	CEP152
614728	Seckel Syndrome Type 6	CEP63
614851	Seckel Syndrome Type 7	NIN
615807	Seckel Syndrome Type 8	DNA2
616777	Seckel Syndrome Type 9	TRAIP
311510	Waisman Syndrome	RAB39B
600118	Warburg Micro Syndrome 1	RAB3GAP1
614225	Warburg Micro Syndrome 2	RAB3GAP2
614222	Warburg Micro Syndrome 3	RAB18
615663	Warburg Micro Syndrome 4	TBC1D20
193520	Watson Syndrome	NF1
603736	OHDO syndrome	KAT6B
615510	Alacrima, Achalasia, And Mental Retardation Syndrome	GMPPA
244450	Blepharophimosis-Ptosis-Intellectual Disability Syndrome	UBE3B
300831	CK Syndrome	NSDHL
213980	Craniofacial Dysmorphism, Skeletal Anomalies, And Mental Retardation syndrome	TMCO1
251300	Galloway-Mowat Syndrome	WDR73, ZNF592
259050	Ossified Ear Cartilages With Mental Deficiency, Muscle Wasting, And bony Changes	ZBTB20
609579	Scaphocephaly, Maxillary Retrusion, And Mental Retardation	FGFR2
615328	Shaheen Syndrome	COG6
612447	Skeletal Defects, Genital Hypoplasia, And Mental Retardation	ZBTB16
615879	Tatton-Brown-Rahman Syndrome	DNMT3A
611816	Temple-Baraitser Syndrome	KCNH1
605282	Temtamy Preaxial Brachydactyly Syndrome	CHSY1
616260	Tenorio Syndrome	RNF125
601390	Van Maldergem Syndrome 1	DCHS1
615546	Van Maldergem Syndrome 2	FAT4
616418	Hypomagnesemia, seizures, and mental retardation	CNNM2
616449	Basel-Vanagait-Smirin-Yosef syndrome	MED25
616938	Coffin-Siris Syndrome 5	SMARCE1
616580	Au-Kline Syndrome	HNRNPK
616638	Smith-Kingsmore Syndrome	MTOR
616737	Takenouchi-Kosaki Syndrome	CDC42
NA038	ZNF674-Related X-linked Mental Retardation	ZNF674
617450	Intellectual developmental disorder with gastrointestinal difficulties and high pain threshold	PPM1D

**Table 2:** List of 148 chromosomal abnormalities that can be detected by the designed chip.

Disease	Chromosome	Start	End
Osteogenesis Imperfecta type XVI	11	43400000	48800000
Holoprosencephaly 10	1	214400000	236400000
Periventricular nodular heterotopia 5	5	83500000	98900000
Kleefstra Syndrome	9	137618963	137870016
Alpha-Thalassemia/Mental Retardation Syndrome, Chromosome 16-Related	16	97457	7800000
Sotos Syndrome 1	5	177100001	180076555
Autosomal Dominant Spinocerebellar ataxia 20	11	55800000	63600000
Autosomal Dominant Deafness 51	9	65000000	69300000
Mental Retardation, Autosomal Dominant 1	2	147900001	149000000
Mental Retardation, Autosomal Dominant 20	5	83500001	93000000
Mental Retardation, Autosomal Dominant 39	2	46285	4400000
2q37 Microdeletion Syndrome	2	230100000	242193529
Thrombocytopenia Absent Radius Syndrome	1	143200001	147500000
Chromosome Xp21 Deletion Syndrome	X	24900000	37800000
Open Angle Glaucoma 1P	12	57700000	67300000
46,XY Sex Reversal 4	9	14840	2200000
46,XY Sex Reversal 10	17	64600000	72900000
46,XX Sex Reversal 2	17	69100000	76800000
46,XX Sex Reversal 3	X	134500000	138900000
Phelan-McDermid Syndrome	22	49100001	50818468
Schizophrenia 16	7	155200000	158937659
Smith-Magenis Syndrome	17	16100001	21323189
Y-linked Spermatogenic Failure 1	Y	21867871	26600000
Sensorineural deafness and male infertility	15	43300000	44500000
Split-Hand/Foot Malformation 3	10	95300000	104000000
Williams Syndrome	7	72700000	77900000
22q11.2 Deletion Syndrome	22	17565972	21700000
Angelman Syndrome	15	20743536	25500000
Wilms Tumor-Aniridia-Genital Anomalies-Retardation Syndrome	11	31000000	36400000
Chromosome 3q13.31 Deletion Syndrome	3	113700000	117600000
Amme Complex	X	104500000	109400000
Choroideremia, Deafness, And Mental Retardation	X	76800000	99100000
Chromosome 10q23 Deletion Syndrome	10	80300000	95300000
Chromosome 10q26 Deletion Syndrome	10	117300000	133797422
Chromosome 13q14 Deletion Syndrome	13	39500000	54700000
Chromosome 14q11-q22 Deletion Syndrome	14	20937674	57600000
Chromosome 15q11.2 Deletion Syndrome	15	20743536	25500000
Chromosome 15q11-q13 Duplication Syndrome	15	20743536	25500000
Chromosome 15q13.3 Deletion Syndrome	15	30900000	33400000
Chromosome 15q24 Deletion Syndrome	15	74900001	76300000
Chromosome 15q25 Deletion Syndrome	15	78000000	88500000
Chromosome 15q26-Qter Deletion Syndrome	15	88500000	101991189
Chromosome 16p11.2 Deletion Syndrome, 220-Kb	16	28500000	35300000
Chromosome 16p11.2 Deletion Syndrome, 593-Kb	16	28500000	35300000
Chromosome 16p11.2 Duplication Syndrome	16	28500000	35300000
Chromosome 16p12.1 Deletion Syndrome, 520-Kb	16	16700000	28500000
Chromosome 16p12.2-p11.2 Deletion Syndrome, 7.1- To 8.7-Mb	16	21200000	35300000
Chromosome 16p13.3 Deletion Syndrome, Proximal	16	97457	7800000
Chromosome 16p13.3 Duplication Syndrome	16	97457	7800000
Chromosome 16q22 Deletion Syndrome	16	66600000	74100000
Chromosome 17p13.1 Deletion Syndrome	17	6500000	10800000
Chromosome 17p13.3, Centromeric, Duplication Syndrome	17	11232	3400000
Chromosome 17p13.3, Telomeric, Duplication Syndrome	17	11232	10800000
Chromosome 17q11.2 Deletion Syndrome, 1.4-Mb	17	27400000	33500000
Chromosome 17q12 Deletion Syndrome	17	33500000	39800000
Chromosome 17q12 Duplication Syndrome	17	33500000	39800000
Chromosome 17q21.31 Duplication Syndrome	17	42800000	46800000
Chromosome 17q23.1-q23.2 Deletion Syndrome	17	59500000	63100000
Chromosome 17q23.1-q23.2 Duplication Syndrome	17	59500000	63100000
Chromosome 18p Deletion Syndrome	18	2656065	14852479
Chromosome 18q Deletion Syndrome	18	19321535	77918489
Chromosome 19p13.13 Deletion Syndrome	19	12600000	13800000
Chromosome 19q13.11 Deletion Syndrome	19	31900000	35100000
Chromosome 1p32-p31 Deletion Syndrome	1	60800001	68500000
Chromosome 1p36 Deletion Syndrome	1	948944	27600000
Chromosome 1q21.1 Deletion Syndrome, 1.35-Mb	1	143200000	147500000
Chromosome 1q21.1 Duplication Syndrome	1	143200000	147500000
Chromosome 22q11.2 Deletion Syndrome, Distal	22	17565972	25500000
Chromosome 22q11.2 Duplication Syndrome	22	17565972	25500000
Chromosome 22q13 Duplication Syndrome	22	37200000	50818468
Chromosome 2p12-p11.2 Deletion Syndrome	2	74800000	91800000
Chromosome 2p16.1-p15 Deletion Syndrome	2	54700000	63900000
Chromosome 2p16.3 Deletion Syndrome	2	47500001	52600000
Chromosome 2q31.1 Duplication Syndrome	2	168900000	177100000
Chromosome 2q31.2 Deletion Syndrome	2	177100000	179700000
Chromosome 2q35 Duplication Syndrome	2	208200000	230100000
Chromosome 3pter-p25 Deletion Syndrome	3	238269	16300000
Chromosome 3q29 Deletion Syndrome	3	192600000	198295559
Chromosome 3q29 Duplication Syndrome	3	192600000	198295559
Chromosome 4q21 Deletion Syndrome	4	75300000	87100000
Chromosome 4q32.1-q32.2 Triplication Syndrome	4	154600000	163600000
Chromosome 5p13 Duplication Syndrome	5	28900000	42500000
Chromosome 5q Deletion Syndrome	5	150400001	153300000
Chromosome 5q12 Deletion Syndrome	5	59600000	67400000
Chromosome 6pter-p24 Deletion Syndrome	6	393143	13400000
Chromosome 6q11-q14 Deletion Syndrome	6	59800000	87300000
Chromosome 6q24-q25 Deletion Syndrome	6	138300000	160600000
Chromosome 7q11.23 Deletion Syndrome, Distal, 1.2-Mb	7	72700000	77900000
Chromosome 8q12.1-q21.2 Deletion Syndrome	8	60600000	85900000
Chromosome 8q21.11 Deletion Syndrome	8	72000000	74600000
Chromosome 8q22.1 Duplication Syndrome	8	92300000	97900000
Chromosome 9p Deletion Syndrome	9	14840	43000000
Chromosome Xp11.22 Duplication Syndrome	X	50100000	54800000
Chromosome Xp11.23-p11.22 Duplication Syndrome	X	47600000	54800000
Chromosome Xp11.3 Deletion Syndrome	X	42500000	47600000
Chromosome Xp22 Deletion Syndrome	X	21900001	24900000
Chromosome Xq26.3 Duplication Syndrome	X	134500000	138900000
Chromosome Xq27.3-q28 Duplication Syndrome	X	143000000	154774947
Chromosome Xq28 Duplication Syndrome	X	148000000	154774947
Digeorge Syndrome	22	17565972	21700000
Frias Syndrome	14	50400000	57600000
Glass Syndrome	2	196600001	202500000
Homozygous 11p15-P14 Deletion Syndrome	11	193811	31000000
Hypertrichosis, Congenital Generalized	X	138900000	141200000
Hypertrichosis, Congenital Generalized, With Or Without Gingival Hyperplasia	17	66200000	72900000
Hypotonia-Cystinuria Syndrome	2	41500000	47500000
Jacobsen Syndrome	11	110600000	121300000
Mesomelia-Synostoses Syndrome	8	65100000	72000000
Miller-Dieker Lissencephaly Syndrome	17	11232	3400000
Monosomy 7 Of Bone Marrow	7	60100000	159345973
Moyamoya Disease 4 With Short Stature, Hypergonadotropic Hypogonadism, And Facial Dysmorphism	X	148000000	154774947
Nablus Mask-Like Facial Syndrome	8	92300000	97900000
Omphalocele, Autosomal	1	60800000	68500000
Opitz GBBB Syndrome, Type II	22	23100001	25500000
Otodental Dysplasia	11	63600000	77400000
Pigmented Nodular Adrenocortical Disease, Primary, 4	19	852319	19900000
Polycystic Kidney Disease, Infantile Severe, With Tuberous Sclerosis	16	97457	7800000
Polyposis Syndrome, Hereditary Mixed, 1	15	43300000	59000000
Potocki-Lupski Syndrome	17	16100000	22700000
Potocki-Shaffer Syndrome	11	43400000	48800000
Prader-Willi Syndrome	15	20743536	25500000
Split-Hand/Foot Malformation 1	7	91500000	98400000
Thrombocytopenia, Paris-Trousseau Type	11	110600000	121300000
Trichorhinophalangeal Syndrome, Type II	8	116700000	126300000
Verheij Syndrome	8	138900001	145138636
Williams-Beuren Region Duplication Syndrome	7	72700000	77900000
WAGRO Syndrome	11	31000000	43400000
Wolf-Hirschhorn Syndrome	4	331763	4500000
Congenital Microcoria	13	94400000	101100000
Chromosome 11p13 Deletion Syndrome, Distal	11	31000000	36400000
Chromosome 14q32 Duplication Syndrome, 700-Kb	14	89300000	106322333
Chromosome 15q14 Deletion Syndrome	15	33400000	39800000
Chromosome 16p13.2 deletion syndrome	16	7800000	10400000
Desanto-Shinawi Syndrome	10	24300001	29300000
Yuan-Harel-Lupski Syndrome	17	10800000	22700000
2q33.1 deletion syndrome	2	196600001	202500000
8p23.1 deletion syndrome	8	6300001	12800000
8p23.1 duplication syndrome	8	10167881	10943836
12p13.33 Microdeletion Syndrome	12	1080000	1346471
12q14 microdeletion syndrome	12	65071919	68645525
16p13.11 recurrent microdeletion (neurocognitive disorder susceptibility locus)	16	14986684	16486684
16p13.11 recurrent microduplication (neurocognitive disorder susceptibility locus)	16	14986684	16486684
16p11.2-p12.2 microduplication syndrome	16	21475060	29284077
16p11.2-p12.2 microdeletion syndrome	16	21512062	30199854
Xq28 Microduplication	X	153287263	153363188
Xq28 Microduplication	X	153624563	153881853
Leri-Weill dyschondrostosis (LWD) - SHOX deletion	X	215902	3261874
Leri-Weill dyschondrostosis (LWD) - SHOX deletion	Y	2655020	5605993

## Materials and methods

### Sample information

A total of 100 samples were gathered for this study. Because we designed the chip to capture almost all disease-causing genes (Table 1), the samples are collected based on the patients who would like to participant in this study in the hospital and are essentially unbiased. In order to assess the stability of the chip, we selected two samples, S77 and S78, for inter-batch and intra-batch stability evaluation. In addition, samples S79, S80, S81, and S82 were selected to evaluate the coverage and depth of the target area under the BGISEQ-500 platform. 86 patients were selected from the clinical cases. Among them, 52 cases were diagnosed, whereas 34 cases were not. In addition, 12 samples that have been tested for CNVseq were selected, and the results were in accordance with the known positive samples in the disease area shown in Table 2. The ability of the chip to detect chromosomal abnormalities was evaluated. All adult participants and parents of minors registered in the study have obtained written informed consent. The project and research programs involving human tissues were approved by the BGI Ethics Committee (BGI-IRB 16098).

### Chip design

In this study, a chip was designed to detect not only SNP, INDEL and large intragenic deletion, but also 148 chromosomal abnormalities from DECIPHER and OMIM databases by adding capture fragments in specific regions. The design steps of the capture region are as follows: (I) Design of capture region for single-gene diseases: concerning that genes usually correspond to multiple transcripts, first, we select the most common or the longest transcript for each gene as the transcript representing the gene. Then we select all the coding sequence (CDS) regions with each CDS region extending 10 base pairs (bps) on both sides to detect splicing variation^7^. The untranslated region (UTR) is large, and most of the region cannot be annotated. The chip does not capture the UTR. The transcripts selected according to the above principles may not include all the functional regions of the other transcripts of the gene, and therefore may not harbor all of the functional regions; (II) Design of chromosomal abnormality detection capture region: firstly, the variant region of each chromosomal abnormality and all genes in the region are determined according to the database, and then the most common or the longest transcript is selected for each gene. All functional regions of the major pathogenic genes of the mutated region are all intended to be contained within the chip. The capture regions of other non-major pathogenic genes are based on the following principles: (i) For variant regions containing less than or equal to 15 genes, we randomly select 100 bps on CDS of each gene for capturing; (ii) For variant regions containing more than 15 genes and less than 40 genes, we randomly select 100 bps on CDS of each gene from two out of every three genes for capturing; (iii) For variant regions containing more than or equal to 40 genes, we randomly select 100 bp on CDS of every other gene for capturing. The coverage region of the probes is about 10 million bps, and an estimated 0.33% of the human reference genome is captured per sequence run.

### Experiments and sequencing

In this experiment, genomic DNA was first extracted from whole blood, and qualified DNA was subjected to library preparation^6^. The library was prepared by disrupting 1 μg of genomic DNA into a small fragment of 200–300 bps of DNA. The fragment selection product was quantified using Qubit. The initial amount of DNA was adjusted to 50 ng according to the measured concentration, then TE buffer was added to make up the total volume to 40 μL. The end repair is then performed and the base “A” is ligated at the 3’ end so that the DNA fragment can be ligated to Barcode. The library constructed by Pre-PCR was used to enrich the target region with the probe designed in this study. Pooling and mixing were performed according to 1 µg sample amount per chip. Then hybridization and elution were performed according to the manual (Roche NimbleGen, USA) followed by the PCR amplification. After the purification with AMPure XP Beads (Beckman Coulter, USA), 330 ng DNA library was subjected to cyclization, and then DNA nanospheres were synthesized. After purification, Qubit (Thermo Fisher Scientific, USA) was used to quantify the purified PCR product. A final yield of single-stranded loops ranging from 33 to 132 ng was considered qualified. Sequencing was performed using the BGISEQ-500 platform, and data analysis and interpretation of the results were performed based on the sequencing data^11,12^. The library preparation was separately performed for each replicate of each sample. Concerning the inter-batch and intra-batch stability of each sample, the inter-batch assessment used three different chips for capturing three technical replicates respectively; while the intra-batch assessment used the same chip for capturing three technical replicates with specific barcodes simultaneously. The data that support the findings of this study have been deposited in the CNSA (https://db.cngb.org/cnsa/) of CNGBdb with accession code CNP0000378.

### Bioinformatics analysis and variant identification

The process of bioinformatics analysis includes data filtering, alignment, variant detection, and result annotation. The raw data were first evaluated for quality to remove low-quality and adapter contaminated reads. The valid data was then mapped to the human reference genome (HG19) using Burrows Wheeler Aligner (BWA)^13^. The PCR-induced duplication was eliminated using Picard software. SNVs and Indels were tested using the Haplotypecaller module in Genomic Analysis Toolkit (GATK)^14,15^. Intra-gene deletions and duplications were identified by comparing the average depth between samples in the same batch. The variants were then annotated, using databases including dblocal (a database of variant frequencies for 100 normal human samples)^6^, dbSNP (http://www.ncbi.nlm.nih.gov/SNP/), HapMap (http: //hapmap.ncbi.nlm.nih.gov/), dbNSFP (http://varianttools.sourceforge.net/Annotation/DbNSFP), and 1000 Genomes (http://www.1000genomes.org/). The criteria for detecting variations in this study: 1. Select known high-frequency pathogenic variants; 2. Filter variations by population frequency, usually <0.01; 3. Refer to databases such as HGMD and clinvar to screen for loci with reported pathogenicity; 4. Clarify the pathogenic mechanism of the gene using the clingen database and identify deleterious mutations; 5. Select sites with high pathogenicity scores based on the results of SIFT, polyphen, varseak, and other prediction software; 6. Combine the patient’s phenotypic and the inheritance pattern of the disease, discovering loci that could ultimately explain the clinical symptoms of the patient. In addition, we used CNVkit to detect chromosomal abnormalities^16^. In this study, CNVkit was mainly used to detect the deletion and duplication of large fragments. We kept using the default parameters, and the cases and controls were used in the pipeline. Finally, suspicious variants were screened, interpreted, and validated to generate the final data. The bioinformatic analysis method of CNVseq refers to the previous report^17^. The clinical evaluation of the CNVseq results was based on guidelines prepared by the American College of Medical Genetics (ACMG)^18^. Variants were named in reference to the International Cytogenetic Nomenclature International System (ISCN) standard. Description of the stability evaluation method within and between batches: we sequenced the samples in 3 technical replicates (batches) and the same batch separately, and ensured that the sample concentration was consistent before chip capture, after hybridization and elution, and in the final sequencing experiment, thereby reducing fluctuations in sequencing depth accordingly. We used the same method to apply data filtering, comparison, deduplication mark, local weight comparison, Indel region weight comparison, base quality value recalibration, and variant detection for each sample based on the GATK best practices pipeline (https://www.broadinstitute.org/gatk/guide/best-practices.php). For variant detection, we used the parameter -out_mode EMIT_ALL_SITES to output all the site information in our capture area and removed the sites that were not covered by a single sample three times on the machine, which is why our total number of sites will be slightly smaller than the size of our capture area. Then we counted whether the genotypes of each site in each sample were exactly the same, or the same twice, or completely different.

### Homology model construction and protein stability prediction

To investigate the effects of disease-associated variants on protein structure and function, we performed protein modeling analysis. This study used the rapid modeling module of yasara (version 17.1.28) software to automatically implement multi-template search and comparison to complete hybrid modeling. The protein model was repaired using the foldx plug-in, and the mutated protein model was constructed to obtain a high-confidence structural model. The foldx plug-in was used to calculate the difference between the energy of the wild-type protein and mutant protein (ΔΔG = Δgmut-Δgwt), and a value of ΔΔG greater than 1.6 kcal/mol was considered to have a significant effect on protein stability^19^. All structural analysis and image rendering were performed using PyMOL (version 2.2.0).

## Results

### Clinical sample depth determination

**Fig. 1:**
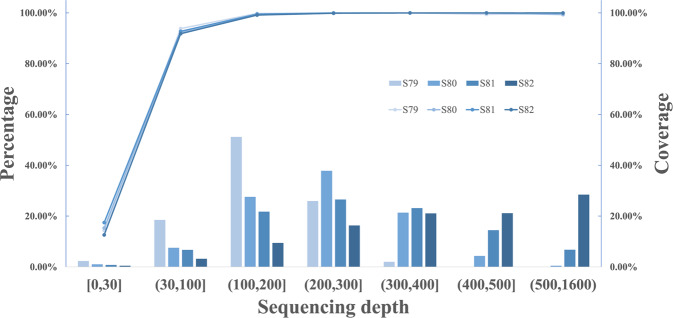
Relationship between Sequencing depth and coverage in CDS region. The columns indicate the proportional distributions of CDS regions with different sequencing depths for sample S79 (157× average), sample S80 (231× average), sample S81 (277× average), and sample S82(380x average), respectively (refer to the left coordinate). The solid dots (circles) represent the average coverage in CDS regions with different sequencing depths (refer to the right coordinate).

In this study, four samples S79, S80, S81 and S82 were selected. The original depths of the samples were 281.46×, 413.23×, 569.57×, and 714.32×, respectively. The average sequencing depths after removing the duplication were 157×, 231×, 277×, and 380×, respectively. All CDS regions were quantified by parameters with sequencing depth and coverage. The proportion and coverage of CDS regions of four samples with sequencing depths between 0 and 30x were 2.29% CDS regions of S79 with 14.33% coverage, 1% CDS regions of S80 with 15.33% coverage, 0.76% CDS regions of S81 with 17.45% coverage, and 0.48% CDS regions of S82 with 12.59% coverage, respectively (Fig. 1). When the sequencing depth reached 30× or more, the coverage was greatly improved. Between 30× and 100× sequencing depths, the coverage of CDS regions of S79, S80, S81, and S82 were 93.84%, 92.29%, 92.75% and 91.81%, respectively. When the depth was greater than 100×, the coverage of all samples can reach more than 99%. A total of 45,527 CDS regions were analyzed, of which 43,192 areas were able to obtain 100% coverage; a total of 2335 areas did not achieve 100% coverage, but it can be seen that, as the depth increases, the coverage increases. In the 45,527 captured regions, 154 CDSs had zero coverage regardless of the read depth. Among these 154 CDSs, CDS1, the first coding DNA sequence, accounts for 87%. We know that the GC content from 5′ untranslated regions to 3′ untranslated regions along human genes gradually decrease^20^. The CDS1 area is next to the 5′ UTR area, possibly because the higher GC content of 5′ UTR affected the capture of the CDS1 area. Based on the above results, it is recommended that on the BGISEQ500 sequencing platform, the average depth of sequencing of the samples using the customized chip of this study should preferably reach 100 X or more after the removal of the duplication.

### Inter-batch and intra-batch stability assessment

**Fig. 2:**
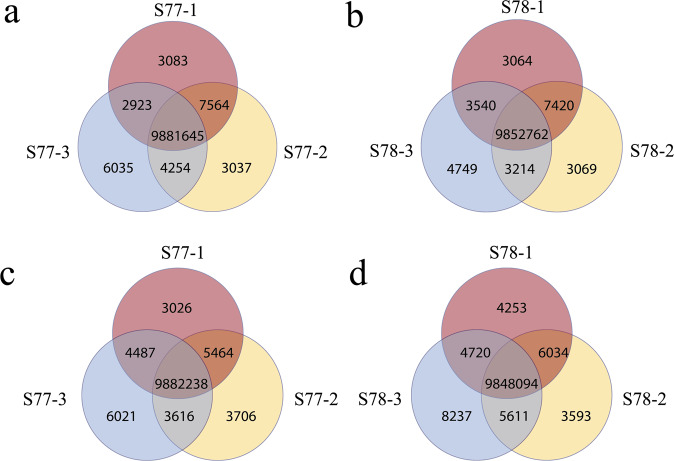
Evaluation of the stability of our method. Venn diagram of S77 (**a**) and S78 (**b**) sequenced three times in the same batch. Venn diagram of S77 (**c**) and S78 (**d**) sequenced three times in three batches.

In this project, sample S77 and sample S78 were sequenced in three batches to evaluate the stability among batches; each sample was sequenced three times to evaluate the stability within the batch. We used the parameter—out_mode EMIT_ALL_SITES to output all the locus detection information in the capture region. Genotypic consistency of loci in different batches of the same sample and the same batch of repeated samples was analyzed. For batch-to-batch stability, the total number of loci was 9,903,792 for sample S77, the intersection of three different batches was 9,881,645, the stability was 99.78% (Fig. 2a); total number of loci was 9,874,160 for sample S78, and 9,852,762 for the intersection of three separate batches with 99.78% for stability (Fig. 2b). In this experiment, we defined stability as the ratio of sites identified in all three technical replicates. For intra-batch stability, the total number of loci in sample S77 was 9,904,450, and the number of intersection loci of three samples in the same batch was 9,882,238, with 99.78% stability (Fig. 2c); for sample S78, the total number of loci was 9,877,841, and the number of intersection loci of three samples in the same batch was 9857 175, the stability was 99.79% (Fig. 2d). From the above data, it is confirmed that the stability of the customized chip is quite good among batches and within batches on the BGISEQ500 sequencing platform. To evaluate the accuracy of this technique, we compared the SNPs of YH cell line samples tested using targeted NGS with the genotyping results obtained using Illumina’s Human Zhonghua-8 bead Chips (SNP Array). We selected the common locus between the SNP array and the chip designed in this experiment for accuracy analysis. A total of 3664 SNPs were detected in YH cell line, and 99.54% (3647/3664) of the genotypes at the selected loci were consistent with the results of SNP Array, demonstrating the high accuracy of this method.

### Variant information in clinical samples

Using targeted next-generation sequencing (NGS), we obtained high-quality sequences of 86 samples. Variant-related information was obtained after the completion of the reference sequence alignment and variant detection. In this study, 67 disease-related variants were identified in 52 patients, including 49 missense variants, 8 frameshift variants, 5 splicing variants, 3 intra-gene deletion and duplication, and 2 whole gene deletions. Of the 67 variants, 36 have been reported and 31 have been reported for the first time. Table S1 summarizes the disease-related variant information for 52 samples.

**Table 3:** Details of CNV detection results at different sequencing depths.

ID	CNVseq results	Region size	Original depth test result	Region size	10^7^ reads test results	Region size	2 × 10^7^ reads test results	Region size
S64	46,XX,del(22q13.33).seq[GRCh37/hg19](49,453,028-51,181,061)×1	1.7Mb	chr22:49411085-51244066*1	1.83Mb	chr22:49411085-51244066*1	1.83Mb	chr22:49411085-51244066*1	1.83Mb
S65	46,XY,del(17p11.2).seq[GRCh37/hg19](16,670,884-20,419,201)×1	3.74Mb	chr17:16842851-20502607*1	3.66Mb	chr17:16842851-20502607*1	3.66Mb	chr17:16842851-20502607*1	3.66Mb
S66	46,XY,del(17p11.2).seq[GRCh37/hg19](16,606,723-20,391,194)×1	3.78Mb	chr17:16670468-20347331*1	3.68Mb	chr17:16842851-20502607*1	3.66Mb	chr17:16842851-20502607*1	3.66Mb
S67	46,XX,del(22q11.21).seq[GRCh37/hg19](18,741,659-21,657,982)×1	2.92Mb	chr22:18614413-20508931*1	1.89Mb				
			chr22:20609931-21731017*1	1.12Mb	chr22:20609931-21731017*1	1.12Mb	chr22:20609931-21731017*1	1.12Mb
S68	46,XX,del(15q11.2q13.1).seq[GRCh37/hg19](23,263,992-28,754,447)×1	5.49Mb	chr15:23565353-28863016*1	5.30Mb	chr15:23565353-28863016*1	5.3Mb	chr15:23565353-28863016*1	5.30Mb
S69	46,XX,del(10q26.13q26.3).seq[GRCh37/hg19](127,004,799-135,404,771)×1	8.40Mb	chr10:127003564-135524247*1	8.5Mb	chr10:127003564-135524247*1	8.52Mb	chr10:127003564-135524247*1	8.52Mb
	46,XX,dup(Xq28).seq[GRCh37/hg19](147,992,184-154,873,016)×3	6.88Mb	chrX:147986298-155260060*3	7.27Mb	chrX:147986298-155260060*3	7.27Mb	chrX:147986298-155260060*3	7.27Mb
			chr7:2962258-3301347*3	339Kb			chr7:2962258-3301347*3	339Kb
S70	46,XY,del(16p11.2).seq[GRCh37/hg19](29,594,664-30,194,495)×1	599.83Kb	chr16:29530680-30199907*1	669Kb			chr16:29530680-30199907*1	669Kb
	46,XY,dup(17q12).seq[GRCh37/hg19](34,819,853-36,390,721)×3	1.57Mb	chr17:34726348-36481838*3	1.76Mb	chr17:34726348-36481838*3	1.76Mb	chr17:34726348-36481838*3	1.76Mb
S71	46,XX,del(11q24.1q25).seq[GRCh37/hg19](122,983,948-134,892,605)×1	11.91Mb	chr11:123065611-134946016*1	11.88Mb	chr11:122969170-134946016*1	11.98Mb	chr11:122969170-134946016*1	11.98Mb
			chr1:151319306-151403327*3	84Kb	chr1:151319306-151403327*3	84Kb	chr1:16370978-16383421*3	12Kb
							chr1:151319306-151403327*3	84Kb
S72	46,XX,del(8q13.2q13.3).seq[GRCh37/hg19](69,890,529-72,583,628)×1	2.69Mb	chr8:69870092-72601140*1	2.73Mb	chr8:69870092-72601140*1	2.73Mb	chr8:69870092-72601140*1	2.73Mb
S73	46,XY,del(17p13.3).seq[GRCh37/hg19](1-2,175,329)×1	2.18Mb	chr17:500-2075128*1	2.07Mb	chr17:500-2075128*1	2.07Mb	chr17:500-2203958*1	2.2Mb
	46,XY,del(7q11.22).seq[GRCh37/hg19](69,783,279-69,952,448)×1	169.17Kb						
S74	46,XY,del(15q11.2q13.1).seq[GRCh37/hg19](23,247,632-28,962,765)×1	5.72Mb	chr15:20044632-29010979*1	8.97Mb	chr15:20044632-29010979*1	8.97Mb	chr15:20044632-29010979*1	8.97Mb
S75	46,XY,dup(7q11.23).seq[GRCh37/hg19](72,470,639-74,438,633)×3	1.97Mb	chr7:72437188-74133270*3	1.70Mb	chr7:72437188-74202442*3	1.77Mb	chr7:72437188-74202442*3	1.77Mb
			chr19:41349691-41356341*1	6KB	chr19:41245252-41356341*1	111Kb	chr19:41245252-41356341*1	111Kb

### Chromosome abnormality detection

This study used CNVkit software to detect chromosomal abnormalities. The software detects CNV based on the read depth method. Therefore, in addition to the original depth, 10,000,000 reads and 20,000,000 reads are randomly extracted, simulating different sequencing depths for CNV copy number and breakpoint position detection. When the data showed that the original depth was 613×, there was one area that remained undetected. This area was chr7: 69, 783, 279–69, 952, 448, with the segment length of 169.17 Kb, and the area is not detected at three different depths, namely 613 × (original depth), 140 × (20,000,000 reads) and 70 × (10,000,000 reads). Therefore, it is speculated that the detection accuracy of the customized chip is insufficient to detect a deletion or a duplication of about 200 kb. In addition, the recommended detection accuracy of CNVkit software is 1 M, and it was found that all the deletions and repetitions above 1 M were detected. CNVkit software detects chromosome deletions and duplications based on the depth of reads. The results also confirmed that as the depth decreases, the number of missed detection areas increases, so it is recommended to ensure a certain amount of depth to help reduce the rate of missed detection. Table 3 shows details of the CNV results information for samples.

**Table 4:** Structural analysis of four mutant proteins.

Gene	Transcription	Location	Nucleotide sequence change	Amino acid sequence change	Modeling Fragment	ΔΔG	Functional Domain
*ANLN*	NM_018685	chr7:36483455	c.3062A>T	p.D1021V	970-1123	−0.138577	Anillin Pleckstrin homology (PH) domain
*CNGB1*	NM_001297	chr16:57935311	c.2921T>G	p.M974R	502-1095	4.07063	effector domain of the CAP family of transcription factors
*UMOD*	NM_003361	chr16:20348705	c.1648G>A	p.V550I	1-620	4.01864	Zona pellucida (ZP) domain
*DSTYK*	NM_015375	chr1:205131207	c.1775G>A	p.R592Q	302-923	0.215561	
*UNC45B*	NM_173167	chr17:33507673	c.2357G>A	p.C786Y	123-931	3.16846	
*COL4A3*	NM_000091	chr2:228173943	c.4664C>T	p.A1555V	1432-1670	2.46126	

### Protein structure prediction and stability results

We performed protein modeling analysis on all genes defined as uncertain significance, of which only six genes were modeled completely and included mutant amino acids in their sequence (Table 4). The six genes were: *ANLN*, *CNGB1*, *UMOD*, *DSTYK*, *UNC45B*, and *COL4A3*. In the structure of ANLN, Asp1021 is located at the carboxy terminus of the Anillin protein and belongs to the PH (Pleckstrin homology) domain, which is necessary for all targeted events^21^. The PH domain is a 120 amino acid protein module that is thought to interact with lipids to mediate protein recruitment to the plasma membrane, and studies have shown that the PH domain is electrostatically polarized^22^. To examine how the p.D1021V variant would affect protein structure, we compared the structure of the wild-type and the mutant, and found that the conformation was basically unchanged. In addition, Gibson’s free energy calculated by foldx also indicates that the variant does not affect the stability of the protein.

The CNGB1 variant p.M974R, UMOD variant p.V550I, and COL4A3 variant p.A1555V were calculated by foldx, with the change in ΔG Gibbs free energy of 4.07063 kcal/mol, 4.01864 kcal/mol, and 2.46126 kcal/mol, respectively. This indicates that these variants affect the stability of the protein.

## Discussion

The study of monogenic hereditary diseases belongs to the field of typical precision medicine. The complex clinical symptoms of monogenic diseases lead to a difficult diagnosis, and most of the pathogenic mechanisms are not clear. Due to the lack of effective treatments, the disease is often fatal, disabling or teratogenic. Diseases such as intellectual disability and growth retardation are often caused by chromosomal abnormalities in addition to the single-gene variants, which are also responsible for monogenic genetic diseases. Therefore, we urgently need an effective detection method that can detect both monogenic genetic variants and chromosome aberrations to facilitate clinical diagnosis and prevention of birth defects. This study designed a chip that can detect up to 4013 single-gene diseases. Compared with previous panel designs^6,7^, we have included more genes related to mendelian diseases when designing the chip to improve our diagnosis rate. In addition, this study also identified 148 common chromosomal disorders by targeting the key genes as well as the random, non-critical genes in chromosomal abnormal regions. In this study, we use MGIEasy Exome Capture V5 Probe to bridge the cost gap between the panel and WES. When their average depth is 200 X, the cost of the panel is approximately 1700 RMB, while the cost of WES is approximately 2300 RMB. The primary reason for the disparity in trial costs between the two is the expense of sequencing. Due to the modest amount of data generated by the panel, the time and personnel costs associated with bioinformatics processing and interpretation will further contribute to the cost differential between the two tests, which we did not specify in this study. Because the amount of data created by the panel is reduced over time, the cost of data storage is reduced. When the sample size hits a particular threshold, it can become rather costly. This project uses the strategy of BGISEQ500 sequencing platform and chip combination. Due to its low cost, the evaluation results indicate that this combination has potential for clinical testing and carrier screening applications.

Sequencing analysis is effective for the diagnosis of rare genetic diseases, but the relationship between effectiveness and cost-effectiveness for the use of comprehensive analyses such as whole genome sequencing and whole exome sequencing remains controversial. Target capture analysis enriches genes or regions of interest and is an analytical method that balances cost and effectiveness. The chip designed in this study encompasses the majority of currently known disease-causing genes that can cause genetic diseases, and can be considered a clinical-grade whole exome. The panel can more effectively target disease-related regions of the human genome and, more importantly, achieve higher sequencing coverage when targeting a group of genes associated with a particular disease phenotype. In this study, for the analysis of CDS coverage, sample coverage reached 99.66% when sequencing depth exceeded 100*, and coverage increased as sequencing depth increased.

Nevertheless, a high-resolution assessment of various WES datasets reveals unequal coverage along the length of exons^23^. Studies reveal that regions with inadequate WES coverage account for around 10% of all CDS regions^24^. We also analyzed the coverage of genes recommended by the American College of Medical Genetics and Genomics (ACMG) for pathogenic variant detection and clinical reporting^25^. Among the 59 genes analyzed, *APOB* CDS1, *DSC2* CDS1, *PRKAG2* CDS5, *RET* CDS1, and *TGFBR1* CDS1 were identified. Regardless of how much the sequencing depth is increased, there is no coverage(Table S1). Six genes, including *KCNH2*, *KCNQ1*, *SDHD*, *TNNI3*, *VHL*, and *WT1*, have been identified inside low-coverage regions in one or more samples, according to additional research^26^. These results imply that low-coverage regions inside functionally significant genes may influence variant detection and subsequent clinical diagnosis.

Moreover, with the same amount of detection data, the chip can obtain higher depth sequencing data than WES, which is advantageous for detecting structural variation at the exon level, and we know that certain diseases, particularly neurological diseases like DMD, can cause by structural variation at the exon level. The clinical application of WGS is still limited at this time for two reasons: first, the interpretation of non-coding regions is extremely limited and relies on scientific research, and second, the cost is prohibitive for the subject. Taking into account the potency ratio, this chip containing nearly all genes with distinct molecular mechanisms continue to be an excellent option. Diseases such as McCune-Albright syndrome are caused by variants in early embryonic somatic cells. Conventional WES analysis, particularly in the clinical setting, may not detect somatic variants. However, this chip has some remaining limitations. In fact, in the era of clinical genomics, where reverse phenotyping has become commonplace^27^, WES can provide early diagnosis and drive treatment options. WES was selected to expedite potential diagnoses and reduce costs associated with multiple tests. Overall, the panel lacks the advantages of a larger number of candidate genes and the ability to reevaluate data on a regular basis, which are offered by WES.

For 86 clinical cases, we first found candidate pathogenic genes in the list of 4,013 diseases based on clinical diagnosis and used the targeted NGS to find pathogenic variants in the candidate genes. If the variant is indeterminate based on the results of the information analysis and database annotations, we will plot the reads and align the reference sequences of the variant sites with a single base resolution. If the variant is still unrecognized, Sanger sequencing or real-time PCR will be performed. However, the pathogenic variants in some cases are still not in the candidate gene. We will find candidate variants in other genes in the target region and to infer the disease in reverse.

In this study, we performed homology modeling on some proteins, hoping to be able to explain the changes in protein structure from variants. Sample S32, 7 years old, shows clinical manifestations of hematuria and C3 glomerulopathy. Missense variation c.3062A>T (p.D1021V) was detected in the *ANLN* (NM_018685.4) gene coding region of the sample as a heterozygote. ANLN gene variant can cause focal segmental glomerulosclerosis type 8 (OMIM#: 616032), which is autosomal dominant, and the main clinical manifestation of glomerular segmental sclerosis, proteinuria, decreased glomerular filtration rate and progressive decline in renal function. Both SIFT and PolyPhe-2 predictions are deleterious variants. The frequency information of c.3062A>T was not found in the dbSNP database, Hapmap database, thousand-person database, or the local database, and there is no documented pathogenicity. In the structure of ANLN, the variant p.D1021V is located in the PH (Pleckstrin homology) domain. Anillin is an actin-binding protein involved in cytokinesis. It interacts with GTP-bound Rho proteins and results in the inhibition of their GTPase activity. The PH domain has multiple functions, but generally involves targeting the protein to an appropriate cellular location or interacting with a binding partner. The PH domain is in electrostatic polarity, because aspartic acid is charged and polar and is often involved in the formation of protein active sites or binding sites, while proline is a non-polar amino acid. Comparing the wild-type and mutant conformations, no changes were found, but there were some differences in the hydrophobic surface. We speculated that the variant affected the electrostatic polarity of the PH domain, resulting in a change in protein function. Therefore, it is speculated that the *ANLN* gene c.3062A>T is a disease-causing variant in the subject.

CNV is widely distributed in human genome and is one of the important pathogenic factors of human diseases. Pathogenic CNV can cause intellectual disability, growth retardation, autism, various birth defects, leukemias, and tumors. Determining the copy number and breakpoint position of the variant region are two crucial aspects of CNV detection. With the advancement of technology, more and more technical means have emerged for CNV detection, but different technology platforms and their corresponding computing strategies have great differences in the accuracy of detected CNV copy number and breakpoint position. The CNVseq method uses genome-wide data, and this study utilizes genomic target region data. Although two methods for detecting CNV are based on the circular binary segmentation algorithm, there are still differences in data correction and comparison. Based on the above reasons, the position of the breakpoints obtained by the two methods is not very consistent, actually the breakpoint positions identified by the two different methods in our study all vary at the kilo bps resolution level. This study uses CNVkit software, which detects CNV based on the read depth method. Therefore, in addition to using the original data, we also simulated different sequencing depths for CNV copy number and breakpoint position detection. As the depth decreases, the number of missed detection areas increases, and a certain number of read lengths help to reduce the rate of missed detection. At breakpoint locations, different depths have no significant effect on the detection of breakpoint locations. Based on a similar capture sequencing technology, the difference between exome sequencing and target capture sequencing during experiments and bio-information analysis is still usually significant. Factors such as the GC content of the probes, the initial DNA concentration, and even the temperature of the chip hybridization in the experiment may affect the number of reads captured by each probe and make a difference in capture efficiency, depth, and coverage. Indeed WESs can accurately detect CNVs above 1 M, but our research based on a specific panel to detect these common chromosomal CNVs is extremely cost-effective.

## Conclusion

In summary, we provide a diagnostic detection tool that combines capture arrays and NGS to capture the coding region of 3043 genes associated with 4013 diseases and detects 148 chromosomal abnormalities by targeting specific regions. The results of the evaluation suggest that our method has high accuracy and stability. Compared with traditional genetic testing methods, it integrates known data about single-gene diseases and frequent chromosomal abnormalities to achieve a “one-step” solution to genetic variants. In our study, perhaps due to high GC content, missing enrichment probes, and other reasons, there are still 154 CDSs regions that cannot be covered at all. The incomplete coverage of regions may be improved by using a high concentration of capture probes that cover difficult-to-enrich regions^28,29^. This technology can be potentially utilized in diagnostic testing to provide an effective basis for clinical diagnosis and genetic counseling and improve the detection rate of diseases.

### Supplementary information

Supplementary Table S1

Supplementary Table S2

Supplementary Information
